# Fast quantum interferometry at the nanometer and attosecond scales with energy-entangled photons

**DOI:** 10.1126/sciadv.adw4938

**Published:** 2025-05-21

**Authors:** Colin P. Lualdi, Spencer J. Johnson, Michael Vayninger, Kristina A. Meier, Swetapadma Sahoo, Simeon I. Bogdanov, Paul G. Kwiat

**Affiliations:** ^1^ Department of Physics, The Grainger College of Engineering, University of Illinois Urbana-Champaign, Urbana, IL, USA.; ^2^ Illinois Quantum Information Science and Technology Center, The Grainger College of Engineering, University of Illinois Urbana-Champaign, Urbana, IL, USA.; ^3^ Department of Electrical and Computer Engineering, The Grainger College of Engineering, University of Illinois Urbana-Champaign, Urbana, IL, USA.; ^4^ Holonyak Micro and Nanotechnology Lab, The Grainger College of Engineering, University of Illinois Urbana-Champaign, Urbana, IL, USA.

## Abstract

In classical optical interferometry, loss and background complicate achieving fast nanometer-resolution measurements with illumination at low light levels. Conversely, quantum two-photon interference is unaffected by loss and background, but nanometer-scale resolution is physically difficult to realize. As a solution, we enhance two-photon interference with highly nondegenerate energy entanglement featuring photon frequencies separated by 177 THz. We observe measurement resolution at the nanometer (attosecond) scale with only *O*(10^4^) photon pairs, despite the presence of background and loss. Our nondestructive thickness measurement of a metallic thin film agrees with atomic force microscopy, which often achieves better resolution via destructive means. With contactless, nondestructive measurements in seconds or faster, our instrument enables metrological studies in optically challenging contexts where background, loss, or photosensitivity are factors.

## Teaser

Quantum entanglement enables contactless measurements at the nanometer scale that tolerate optical loss and background.

## INTRODUCTION

Optical interferometry is an effective technique for high-resolution measurement and imaging; diverse applications include gravitational-wave detection (*1*), long-baseline astronomy (*2*), and optical coherence tomography (*3*). Most of these applications use classical interference, in which an electromagnetic wave travels in a superposition of two paths and either constructively or destructively interferes with itself on a balanced beamsplitter depending on the relative phase between the two paths. While this technique can easily detect relative delays at the nanometer (or, equivalently, attosecond) scale, it is ill suited for contexts with imbalanced path loss and optical background, which reduce the interference visibility, and hence the attainable resolution. In contrast, quantum interference involves two photons incident on the two inputs of a balanced beamsplitter; when the photons are indistinguishable, including in their time of arrival, their bosonic nature induces them to always exit the beamsplitter in the same port (*4*). The visibility of two-photon interference is inherently robust against imbalanced path loss and optical background, motivating its use in quantum optical coherence tomography (*5*, *6*), quantum microscopy (*7*), clock synchronization (*8*, *9*), and other metrological applications. However, the measurement utility can be limited as achieving high resolution typically requires long measurements or ultrabroadband photons. For the former, nanometer-scale resolution has been demonstrated with hours-long measurements (*10*), and for the latter, resolutions range from the submicron (*11*, *12*) to nanometer (*13*) scales, depending on the measurement technique.

Introducing energy entanglement between the two interfering photons reveals an alternative path toward improved resolution with quantum interference (*14*). Conventional two-photon interference features a dip in the probability of two photons exiting the beamsplitter in separate ports, as a function of the relative temporal delay between the two paths incident on the beamsplitter. The attainable measurement resolution is determined by the dip width, inversely related to the photons’ bandwidth. When the photons are energy entangled, the dip is modulated sinusoidally with a period varying inversely with the difference (or “beat note”) between the frequencies of the entangled photons (*15*, *16*), resulting in interference fringes of similar form as those obtained via classical interference. The measurement information acquired per photon pair is therefore greatly increased. This measurement scheme saturates the quantum Cramér-Rao bound, which sets the maximum attainable measurement precision, given a quantum probe state (*14*, *17*, *18*).

In this work, we fully realize the potential of this technique with highly nondegenerate, narrowband entangled photons, performing measurements at the nanometer and attosecond scales in seconds, even in the presence of substantial imbalanced path loss and optical background. While the features of loss and background insensitivity do not require entanglement, but only two-photon interference, the fact that such features persist in the presence of the energy entanglement needed to achieve high resolution makes this methodology superior to classical interference techniques.

## RESULTS

### Theoretical description

Consider the energy-entangled two-photon state

$$|\psi \rangle =\frac{1}{\sqrt{2}}\left({|\omega_{1}\rangle }_{a}{|\omega_{2}\rangle }_{b}+{|\omega_{2}\rangle }_{a}{|\omega_{1}\rangle }_{b}\right)$$ (1)

where $\omega_{i}$ denotes the photon’s angular frequency, proportional to its energy, and *a* and *b* denote the two inputs of a balanced beamsplitter. When these two otherwise identical photons impinge on a beamsplitter, the probability the photons exit in separate beamsplitter outputs and result in a coincidence detection between detectors placed at each output is given by

$$P_{C}=\frac{1}{2}\left\{1-\text{cos}\left[\left(\Delta \omega \right)\tau \right]e^{-2\sigma^{2}\tau^{2}}\right\}$$ (2)

where $\Delta \omega \equiv \omega_{1}-\omega_{2}$ is the angular frequency detuning of the entangled photons, τ is the relative temporal delay between the beamsplitter input paths, and σ is the photons’ angular frequency half bandwidth (see Supplementary Text). In conventional two-photon interference, $\omega_{1}=\omega_{2}$ such that the cosine factor reduces to unity and Eq. 2 reduces to the functional form of the so-called Hong-Ou-Mandel dip. As in (*10*), we set τ to maximize ${\mathrm{dP}}_{C}/d\tau$ such that the interferometer yields the maximum Fisher information when measuring the change in $P_{C}$ induced by a small change in the relative delay δτ. Via the quantum Cramér-Rao bound, the quantum Fisher information provides a lower bound on the SD $\sigma_{\tau }$ of an estimation of δτ, which defines the interferometer resolution. In the ideal case, we have

$$\sigma_{\tau }\geq \frac{1}{\sqrt{N}\sqrt{Q}}=\frac{1}{\sqrt{N}\sqrt{{\left(\Delta \omega \right)}^{2}+4\sigma^{2}}}$$ (3)

with *N* the number of measurements and *Q* the quantum Fisher information. As noted above, increasing *N* or σ has been demonstrated to yield a smaller $\sigma_{\tau }$ (*10*–*13*), but this introduces practical challenges. Instead, with nondegenerate energy entanglement, a large detuning Δω can achieve a comparable $\sigma_{\tau }$, but with narrow-bandwidth photons and far fewer measurements.

### Experimental setup

**Fig. 1.**
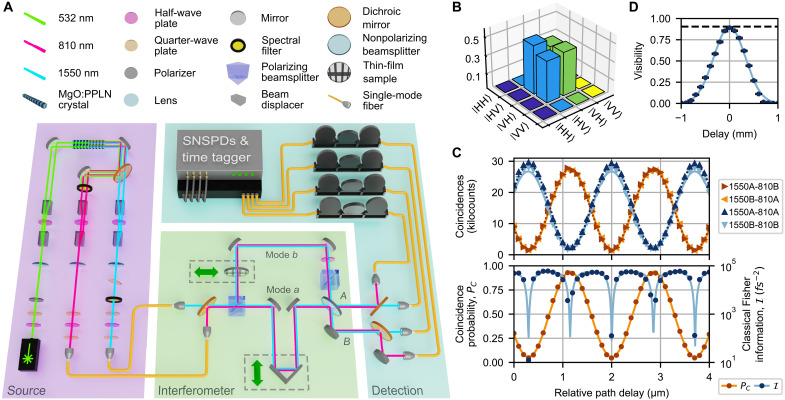
Highly nondegenerate energy-entangled two-photon interferometer. (**A**) The experimental apparatus. In the source module (purple), beam displacers separate a diagonally polarized 532-nm continuous-wave laser beam into horizontally and vertically polarized components. Each component pumps its own magnesium-oxide–doped periodically poled lithium niobate (MgO:PPLN) crystal to generate nondegenerate photon pairs at 810 and 1550 nm via SPDC. One of the two crystals is rotated 90° around the optical propagation axis such that photon pairs from the two crystals are orthogonally polarized. A dichroic mirror separates the two down-converted wavelengths into individual spatial modes, where beam displacers spatially recombine the two polarization components. The resulting polarization-entangled photons are collected into single-mode fibers and transferred to the interferometer module (green), where polarization entanglement is converted into energy entanglement. Energy-entangled photon pairs then interfere on a 50:50 beamsplitter; a tunable path delay controls the relative temporal delay between modes *a* and *b*. The detection module (blue) sorts the exiting photons by wavelength for detection by superconducting nanowire single-photon detectors. (**B**) Density matrix of the nondegenerate polarization-entangled state incident on the PBS in the interferometer module, before conversion into energy entanglement. (**C**) Top panel: Energy-entangled two-photon interference fringes as a function of relative path delay for each coincidence and anticoincidence channel, with sinusoidal fits (4-μm snippet of a 20-μm scan shown; the fits use the full scan). Accidentals are negligible with a mean coincidence to accidental ratio of 1100(200) per measurement for the four channels combined. Bottom panel: The normalized coincidence probability fringe with a sinusoidal fit (solid curve). The corresponding classical Fisher information I is also shown; the solid curve is the theoretical prediction based on the sinusoidal fringe fits. (**D**) Fringe visibility as a function of relative path delay. The exponential fit yields a maximum visibility of 90.5(1.0)% (dashed line).

Our apparatus consists of source, interferometer, and detection modules (Fig. 1A). To generate the required energy-entangled state (Eq. 1), we first generate nondegenerate polarization-entangled photon pairs (*19*). Our source uses a revised version of the beam-displacer geometry described in (*20*) to coherently drive two highly nondegenerate spontaneous parametric down-conversion (SPDC) processes, producing the state

$$\begin{array}{c} |\;\varphi_{\text{SPDC}}\;\rangle =\frac{1}{\sqrt{2}}\left({|\;H\rangle }_{1550}{|\;H\rangle }_{810}+e^{\mathrm{i\varphi}}{|\;V\rangle }_{1550}{|\;V\rangle }_{810}\right) \end{array}$$ (4)

where *H* and *V* are the horizontal and vertical polarization states, the subscripts denote the 1550- and 810-nm photon wavelengths, and φ is an arbitrary relative phase. Type-0 phase matching enables a high source brightness: >10^5^ detected pairs per second per milliwatt.

Photons at each wavelength are separated into individual spatial modes and coupled into their own single-mode fiber for transfer between the source and interferometer modules. The photons are then collimated into free space and multiplexed (via a dichroic mirror) into a common spatial mode incident on the interferometer input. A polarization state tomography at the interferometer input yields a state with a high purity, concurrence, and entangled singlet fraction of 90.3(2), 89.6(2), and 94.8(1)%, respectively (Fig. 1B).

A pair of half-wave and quarter-wave plates at the input of each source collection fiber are set such that the waveplate-fiber system performs the operation $I_{1550}X_{810}$ on $|\;\varphi_{\text{SPDC}}\;\rangle$, making the two wavelengths orthogonally polarized. At the interferometer input, the bit-flipped state passes through a polarizing beamsplitter (PBS) that transmits *H* photons into spatial mode *a* and reflects *V* into *b*. Since the state’s two wavelengths correspond to orthogonal polarizations, it transforms into a superposition of a 1550-nm (810-nm) photon in mode *a* (*b*) and vice versa. After using a half-wave plate to rotate the polarization of the mode *b* photons from *V* to *H*, we obtain the energy-entangled state Eq. 1. With 1550- and 810-nm photons, our detuning $\Delta \omega \equiv \omega_{1}-\omega_{2}=2\pi \;177\;\text{THz}$ is nearly an order of magnitude larger than the previous best result, 2π30.1THz (*21*).

The energy-entangled photons then impinge on a balanced beamsplitter and undergo interference. The relative temporal delay between the beamsplitter input paths (τ) is adjusted by tuning the relative optical path lengths for modes *a* and *b* via an optical trombone in path *a*. The trombone position is controlled by both a piezoelectric nanopositioning stage and a servo actuator, enabling nanometer resolution with centimeters of travel.

Upon interference, the photons exit the beamsplitter in either port *A* or *B*. At each output port, a dichroic mirror separates 1550- and 810-nm photons for coupling into single-mode fiber leading to superconducting nanowire single-photon detectors. Four pairs of coincident detections (1550*A*-810*B*, 1550*B*-810*A*, 1550*A*-810*A*, and 1550*B*-810*B*) are monitored via a time tagger, with the first two corresponding to “coincidence” events (coincident detections in the opposite ports) and the last two “anticoincidence” events (coincident detections in the same port).

With these four detection pairs, we directly measure the normalized coincidence probability $P_{C}$ (Eq. 2) as $N_{C}/\left(N_{C}+N_{A}\right)$ where $N_{C}$ and $N_{A}$ are the total number of coincidence and anticoincidence events, respectively. Directly detecting all interfering photons simplifies previous methods, which relied on extensive characterization of system losses (*14*) or probabilistic detector trees (*21*) to extract $P_{C}$ from a given measurement of $N_{C}$.

Figure 1C shows the interference fringes observed in our experiment. As the relative path delay between modes *a* and *b* is scanned, $P_{C}$ oscillates sinusoidally with a fitted period of 1705.9(2) nm, close to the 1701.87(1) nm period expected from the photon center wavelengths of 810.504(1) nm (measured) and 1547.484(5) nm (inferred via energy conservation). The given errors are based on fitting errors; the slight discrepancy between the fringe and spectral measurements is likely due to systematic errors, e.g., we observe a small drift in the interferometer relative phase (~1 degree per minute; see fig. S6), which would be sufficient to account for the inferred wavelength mismatch. The fitted fringe visibility of 88.9(2)% is close to the ~87.4% expected given our entangled state purity, PBS extinction ratio, and beamsplitter splitting ratio. Last, while the fitted visibilities of the four coincident detection fringes range between 87.7(4) and 89.2(5)%, the individual-detector fringes have visibilities below 1%, indicating that two-photon, not single-photon, interference dominates.

From the four coincident detection fringe data and fits, we extract the corresponding classical Fisher information and estimate the maximum attainable measurement resolution. With 1-mW source pumping power and a 1-s integration time, we observe a mean of 59,000(1000) total coincident detections per measurement. The corresponding resolution is 1.26 nm (1.26 nm/*c* = 4.2 as, where *c* is the speed of light), an 88% saturation of the Cramér-Rao bound.

Figure 1D illustrates a key advantage of introducing energy entanglement to two-photon interference. To achieve nanometer-scale resolution, conventional two-photon interference would require ultrabroadband photons (~177 THz), corresponding to a narrow dip width of ~317 nm full width at half maximum (FWHM), making the dip difficult to locate. In addition, optical systems that support such a large spectral spread are difficult to realize, as is such a broadband SPDC source (*11*–*13*, *22*). In contrast, our experiment uses narrowband photons (e.g., $\sigma_{810}=0.495\left(2\right)$ nm FWHM) such that the modulated interference dip envelope is much wider: 0.76(1) mm FWHM. Nonzero interference visibility over such a large window greatly simplifies initially calibrating the relative path lengths and can enable a very large dynamic range, e.g., by counting fringes.

### Entanglement-enhanced quantum metrology

Our interferometer can achieve nanometer (attosecond) resolution with only *O*(10^4^) detected photon pairs; with a detected pair rate of at least 150,000 per second (enabled by tuning the source pumping power), we can attain nanometer resolution in a timescale of seconds (or less).

**Fig. 2.**
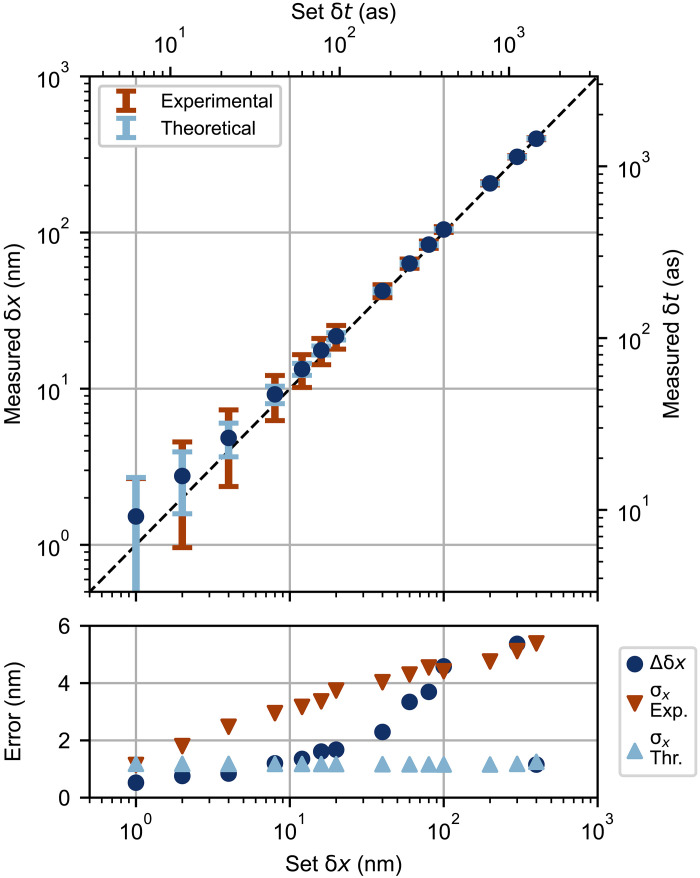
Demonstrating nanometer-scale resolution. Experimental data illustrating measurements at the nanometer (attosecond) scale. Displacements were introduced by a nanopositioning stage displacing a retroreflector; a displacement of δ*x* in the optical path length is therefore realized by translating the nanopositioning stage by δ*x*/2. The bottom panel shows the “error” in terms of both accuracy (Δδx) and precision ($\sigma_{x}$).

To validate the resolution estimated from the fringes in Fig. 1C, we displace the trombone retroreflector by a set displacement δ*x*/2, measure the resulting change in the coincidence probability $P_{C}$ (with a 1-s integration time) and extract the corresponding δ*x*. The top panel of Fig. 2 shows the mean measured δ*x* and its SD $\sigma_{x}$ (for 100 trials) for multiple values of δ*x*. The dynamic range is set by our standard measurement scheme, which is restricted to displacements on one rising or falling fringe, i.e., half of the interference period. As the Fisher information is high near the fringe center (where $P_{C}\approx 0.5$), we typically operate within this region, which spans a few hundred nanometers. The bottom panel shows the corresponding measurement error $\left(\Delta \mathrm{\delta x}\equiv |\;\delta x_{\text{measured}}-\delta x_{\text{set}}\;|\;\right)$ as well as the experimental and theoretical $\sigma_{x}$. We observe nanometer-scale accuracy and precision. We attribute the increase in error and decrease in resolution as δ*x* increases to interferometer drift; all 14 set displacements were measured sequentially from a common zero-displacement point.

**Fig. 3.**
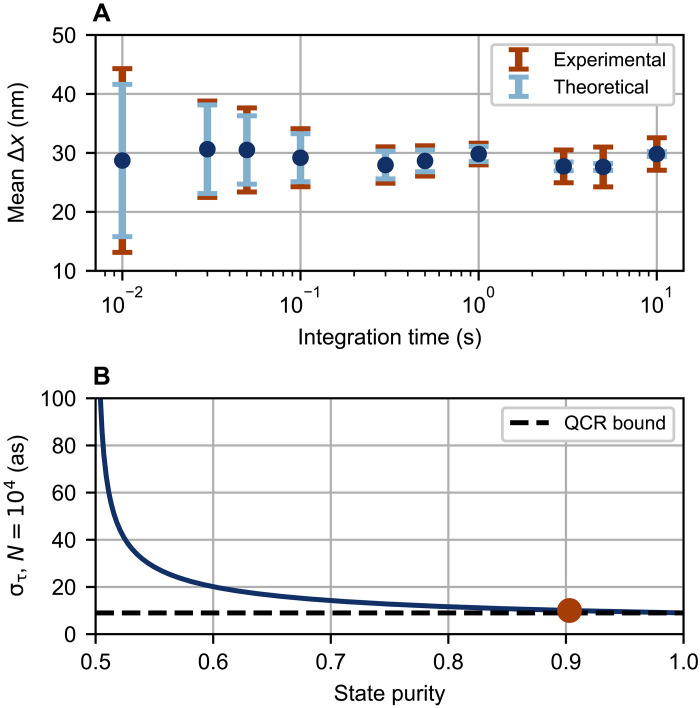
Effects of integration time and state purity on measurement resolution. (**A**) Experimental data illustrating trade-offs between resolution and measurement time. The interferometer displacement Δx is the path-length change introduced by moving from the initial sample position (uncoated region) to the final position (coated region). The “interferometer displacement” is extracted by assuming that the fringe period (in terms of path length) is unchanged by the indices of refraction of the sapphire wafer and nickel coating. Using a sample with a fixed displacement instead of the optical trombone eliminates additional uncertainty from the trombone positioner. Each trial for each data point involves two $P_{C}$ measurements (both with identical integration times), one for each sample position. While the upper bound on the resolution is in principle set by the phase drift of our passively stabilized interferometer, one could perform multiple measurements with a short integration time (where drift is negligible) and attain higher resolution via the standard error of the mean. (**B**) The maximum theoretical measurement resolution $\sigma_{\tau }$ as a function of state purity for *N* = 10^4^, with our result highlighted.

Our measurement resolution is dominated by the frequency detuning Δω and the number of detected photon pairs *N* (Eq. 3). Our high detected photon pair rate therefore enables tuning the tradeoff between the measurement resolution and the integration time (Fig. 3A), which shows the average measured interferometer displacement (Δx) and $\sigma_{x}$ for a fixed set displacement (100 trials each). To introduce the displacement, we insert a sapphire wafer in one path of the interferometer and translate it between two transverse positions with respect to the optical beam; one position corresponds to an uncoated region and the other to a nickel-coated region with a nominal thickness of 5 nm. With a baseline detection rate of 128,000(3000) and 68,000(2000) pairs per second for the uncoated and coated regions, respectively, we achieve an interferometer resolution of $\sigma_{x}=1.8\;\text{nm}$ with a 1-s integration time. As expected, a shorter 0.1-s integration time yields a slightly reduced resolution of 4.9 nm. Longer integration times introduce measurement error from interferometer drift, as our apparatus currently has no active stabilization. For example, increasing the integration time to 10 s yields a resolution of 2.7 nm instead of the 0.4 nm predicted by theory. However, since nanometer-scale resolution is still achievable with shorter integration times, measurements may be made much faster relative to the interferometer drift such that active stabilization is not strictly necessary. Overall, our measurements approach the fundamental resolution limit given our probe state, as dictated by the quantum Cramér-Rao bound, despite an imperfect entangled state purity of 90.3(2)% (Fig. 3B).

**Fig. 4.**
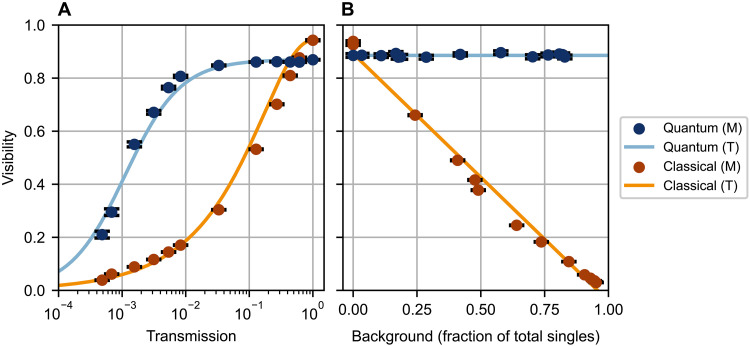
Robustness against imbalanced path loss and optical background. (**A**) Measured (M) interference visibility as a function of transmission in mode *b* of the interferometer for both quantum and classical interference. Solid lines are theoretical (T) predictions. The reduction in two-photon visibility beyond 10 dB of loss is attributed to noise events. Contributions to noise include both accidentals (coincident detections between uncorrelated photons) and a small ${|\;\omega_{1}\rangle }_{a}{|\;\omega_{2}\;\rangle }_{a}$ term in our state (Eq. 1) arising from the finite extinction ratio of our PBS and the imperfect application of $I_{1550}X_{810}$ between the source and interferometer modules. Present in mode *a*, these sources of noise become relevant only when the loss in mode *b* reduces the relative rate of genuine to noise coincident detections. (**B**) Observed interference visibility as a function of optical background, expressed as a fraction of total individual detector clicks (“singles”). Solid lines are fits to theoretical models. Even when nearly 100% of the detector clicks are background events, the quantum visibility is unaffected, whereas the classical visibility is severely degraded.

We also verify the robustness of energy-entangled two-photon interference against imbalanced path loss and optical background. Since quantum interference is a two-photon process, imbalanced path loss acts globally on the two-photon state to reduce the detection rate of two-photon states, with the interference visibility unaffected. Conversely, in classical interference imbalanced loss degrades the photon’s superposition state, diminishing both the photon rate and interference visibility. We introduce tunable optical loss to mode *b* of our interferometer by inserting a PBS after the half-wave plate; the PBS transmission is adjusted by rotating the half-wave plate. Measuring the interference visibility for increasing loss (up to ~33 dB), we observe that the two-photon interference visibility is largely unaffected up to ~10 dB of loss and only slightly affected up to ~20 dB of loss. In contrast, the classical interference visibility (using 1550-nm photons) decreases immediately and precipitously, dropping to approximately half its starting value with only ~10 dB of loss (Fig. 4A). The reduction in quantum visibility for >10 dB of loss is attributed to the presence of noise photons resulting from experimental imperfections; our noise-adjusted theoretical model closely tracks the experimental data.

Optical background can cause coincident detections uncorrelated with actual energy-entangled photon pairs. The resulting accidentals background reduces fringe visibility for the same reason classical interference visibility is degraded by detector background; in both cases, the reduced visibility leads to a decreased measurement resolution. However, unlike classical interference, two-photon interference is measured in coincidence; thus, any background photons would need to appear in the same coincidence window as the photons undergoing interference to register as an accidental event. By using low-jitter detectors and electronics (75-ps mean total FWHM jitter per coincident detection channel) and a tight coincidence window radius (±50 ps), we suppress the per-second background rate by 100 dB while maintaining a high coincidence rate. Since our photons are narrowband, an even greater suppression may be achieved with tight spectral filtering at the detectors; such filtering cannot be leveraged to the same extent for conventional two-photon interference with broadband photons. We experimentally introduce background by shining a halogen lamp onto our apparatus at varying intensities. Even with background photons approaching 100% of all individual detector clicks, we observe unchanged two-photon interference visibility (Fig. 4B). In contrast, repeating the same experiment with classical interference (using 1550-nm photons) results in a 97% visibility reduction.

**Fig. 5.**
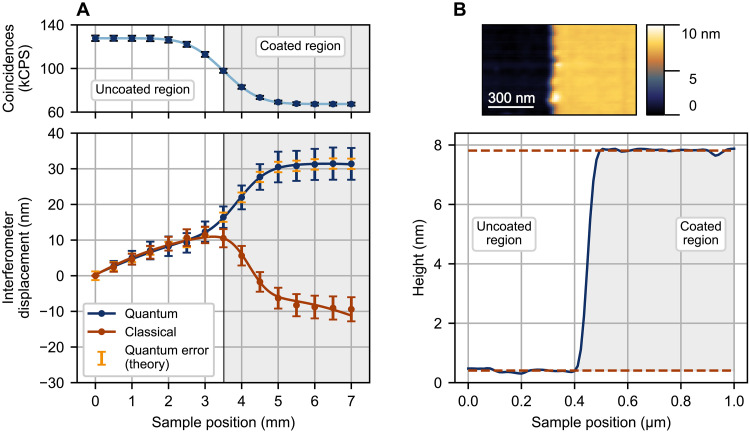
Measuring the nanometer-scale thickness of a lossy metallic thin film. (**A**) Top panel: The coincident detection rate (for quantum interference) as a function of sample position, showing a clear delineation between the uncoated and coated regions. The fit (solid curve) indicates a quantum probe transmission of 52.8(1)%. Bottom panel: The interferometer displacement as a function of sample position, for both quantum and classical interference; the film thickness is related to the interferometer displacement via the refractive index of the film material. The blue and red error bars show the SD for 100 trials and indicate the measurement resolution for the 1-s per-point integration time. The larger error bars for the coated region are due to both a loss-induced reduction in *N* and drift in the interferometer phase as the sample is scanned, as evidenced by the theoretical quantum error bars (yellow), which assume no drift. Solid curves are fits to a theoretical model (see Supplementary Materials). (**B**) Top panel: A false-color AFM image of the sample, showing uncoated (left) and coated (right) regions. Bottom panel: Profile view of top-panel image, showing the average from each horizontal scan line. The red dashed lines indicate the average height for the first and last 340 nm of the bottom and top regions; their mean and SD values yield a step height of 7.4(1) nm. Unlike the interferometric measurements, our AFM measurement was destructive: To address poor edge conditions that rendered nondestructive measurement inaccurate, we had to mechanically remove a small region of the film (using the AFM tip) to expose the substrate before measuring the height difference.

We demonstrate the metrological capabilities of our system by measuring the thickness of a thin metallic film (Ni) on a 3-inch sapphire wafer. We measure the following in transmission: The wafer is placed in the *b* mode of the interferometer and scanned transversely such that the optical beam [with a mean effective 1/*e*^2^ diameter of 1.21(4) mm] moves horizontally across the wafer, from an uncoated region to a coated region. By monitoring the coincidence probability $P_{C}$ as a function of the sample position, we can infer the optical delay introduced by the thin film. Using a calibrated effective refractive index acquired by measuring a calibration sample with a well-defined thickness (see Supplementary Text) and the known interference fringe period, we convert phase to film thickness. The average (100 trials) measured displacement as a function of sample position is shown in Fig. 5A, for both quantum and classical interference. The data fit well to a model describing a Gaussian optical probe scanning over an infinitely sharp step in height on a substrate with a linear wedge and quadratic curvature (see Supplementary Text). From the fit we extract a film thickness of 7(1) nm for the quantum measurement, in excellent agreement with the 7.4(1) nm obtained via atomic force microscopy (AFM), as shown in Fig. 5B. A scanning-stylus profilometry measurement yielded a similar thickness of 5.9(1) nm. In contrast, three-dimensional (3D) optical profilometry could only provide a semiquantitative thickness estimate of 12(1) nm because of measurement noise. The classical interference measurement is even worse, returning a film thickness of −9(1) nm; the deposited film instead appears as an etched substrate. We attribute this inaccurate result to degraded fringe quality due to the lossy film; observed degradations include decreased interference visibility [95.7(1)% → 82.7(2)%]. In contrast, the quantum fringes are largely unchanged [e.g., the quantum visibility is negligibly affected: 88.5(3)% → 88.2(4)%].

## DISCUSSION

Leveraging a bright source of highly nondegenerate energy entanglement, we realize fast, loss-tolerant nanometer-scale metrology at the single-photon level. With only *O*(10^4^) photons required for nanometer-scale (attosecond-scale) resolution, our performance surpasses the state of the art in conventional (nonultrabroadband) quantum interferometry, which requires *O*(10^11^) photons collected over hours to achieve comparable resolution (*10*). Our 177-THz frequency detuning also improves upon the tens of terahertz used in the state of the art, enabling a resolution improvement of two orders of magnitude (*14*, *21*). Our combination of high measurement resolution and robustness against imbalanced loss enabled an accurate thickness measurement of a lossy metallic thin film, an exercise that classical interferometry failed. Although classical interferometry may be made to work with lossy samples in certain situations by calibrating the visibility as a function of sample location, such calibration represents an unnecessary additional step with its own complications. Our method stands out for its contactless (unlike scanning-stylus profilometry), nondestructive nature (unlike AFM in some circumstances), single-photon level of illumination (unlike 3D optical profilometry), and a large scanning range of millimeters rather than micrometers. The AFM measurement was destructive in our case because of the need to remove film material to measure the relative height between the film and substrate for our sample.

Straightforward engineering improvements can enable improved lateral resolution via probe focusing and 2D imaging via raster scanning, both of which have been demonstrated for energy-entangled interferometry (*21*), along with measuring in reflection, as demonstrated in quantum optical coherence tomography experiments (*6*). Overall, our system enables nanometer-scale metrological studies, such as probing lossy, light-sensitive biological samples, for which existing classical techniques are ill suited.

In addition, as discussed in the Supplementary Text, our interferometer can be operated in two additional modes. The first uses nonentangled, nondegenerate photon pairs to observe beating between 1550- and 810-nm single-photon interference fringes. The second involves replacing ∣ψ〉 (Eq. 1) with $|\;\psi \prime \;\rangle \propto \left({|\;\omega_{1}\rangle }_{a}{|\;\omega_{2}\rangle }_{a}+{|\;\omega_{2}\rangle }_{b}{|\;\omega_{1}\rangle }_{b}\right)$, a frequency-dependent variant of the two-photon N00N state (*23*). With this state, the sum (rather than the difference) of the two frequencies determines the interference period (532 nm in our case). These modes enable additional metrological possibilities, such as probing samples opaque at 532 nm but transparent at 810 and 1550 nm.

## MATERIALS AND METHODS

### Theory

#### Quantum Fisher information

For a given measurement of some parameter τ using quantum probe state ∣ψ(τ)〉, the resolution of the measurement $\sigma_{\tau }$ is given by the quantum Cramér-Rao bound

$$\sigma_{\tau }\geq \sigma_{\tau ,\mathrm{QCR}}=\frac{1}{\sqrt{N}}\frac{1}{\sqrt{Q}}$$ (5)

where *N* is the total number of trials in the measurement, and

$$\begin{array}{c} Q=4\left(\langle \frac{\partial \psi \left(\tau \right)}{\partial \tau }\left|\frac{\partial \psi \left(\tau \right)}{\partial \tau }\right.\rangle -{\left|\langle \psi \left(\tau \right)\left|\frac{\partial \psi \left(\tau \right)}{\partial \tau }\right.\rangle \right|}^{2}\right) \end{array}$$ (6)

is the quantum Fisher information (*17*, *18*). Evaluating Eq. 6 with our probe state (see Supplementary Text), we obtain

$$Q={\left(\Delta \omega \right)}^{2}+4\sigma^{2}$$ (7)

which leads to Eq. 3.

#### Classical Fisher information

When performing measurements, it is often convenient to measure the classical Fisher information I rather than the quantum Fisher information *Q*. This is because the classical Fisher information can be directly calculated from the coincidence and anticoincidence probabilities associated with our interference fringes and provides additional information about imperfections in the measurement scheme. The classical Fisher information for a discrete probability distribution P(X;τ) is given by

$$\begin{array}{r} I=E\left[{\left\{\frac{\partial \text{log}\left[P\left(X;\tau \right)\right]}{\partial \tau }\right\}}^{2}\right]\\ \;=\sum_{x}{\left[\frac{\frac{\partial P\left(x;\tau \right)}{\partial \tau }}{P\left(x;\tau \right)}\right]}^{2}P\left(x;\tau \right)\\ \;=\sum_{x}\frac{{\left[\frac{\partial P\left(x;\tau \right)}{\partial \tau }\right]}^{2}}{P\left(x;\tau \right)} \end{array}$$ (8)

where $E\left[\;f\right]$ is the expectation value of f. Evaluating Eq. 8 for the case of ideal energy-entangled two-photon interference (see Supplementary Text), we obtain the single-event Fisher information

$$\begin{array}{c} I=\frac{{\left\{\left(\Delta \omega \right)\text{sin}\left[\left(\Delta \omega \right)\tau \right]+4\sigma^{2}\tau \text{cos}\left[\left(\Delta \omega \right)\tau \right]\right\}}^{2}}{e^{4\sigma^{2}\tau^{2}}-{\text{cos}}^{2}\left[\left(\Delta \omega \right)\tau \right]} \end{array}$$ (9)

In the limit of τ → 0, we recover

$$\underset{\tau \to 0}{\text{lim}}I={\left(\Delta \omega \right)}^{2}+4\sigma^{2}$$ (10)

identical to the quantum Fisher information *Q* (Eq. 7). Experimentally, I is calculated via Eq. 8, where the probabilities *P_AA_*, *P_AB_*, *P_BA_*, and *P_BB_* correspond to the four measured coincidence and anticoincidence probability fringes. We note that, for calculational convenience, we assume in our normalization of each probability fringe that the fringe is centered around *P* = ^1^/_4_, which is consistent with our observed fringes.

#### Experimental saturation of the Cramér-Rao bound

While the quantum Cramér-Rao bound describes the fundamental resolution limit a measurement scheme can achieve for a given probe state, in practice, experimental imperfections result in the observed resolution being below the bound. In our system, the primary contribution to resolution degradation is imperfect entanglement purity, which leads to reduced interferometer visibility and therefore resolution. A mixed energy-entangled state with purity $\left(1+\varepsilon^{2}\right)/2$ produces interference fringes

$$P_{C}\left(\tau \right)=\frac{1}{2}\left\{1-\varepsilon \text{cos}\left[\left(\Delta \omega \right)\tau \right]e^{-2\sigma^{2}\tau^{2}}\right\}$$ (11)

A full derivation is given in the Supplementary Text. These fringes have visibility ε, allowing this to also illustrate the effect of imperfect interference. By error propagation

$$\text{Var}\left[P_{C}\right]={\left(\frac{\partial P_{C}}{\partial \tau }\right)}^{2}{\sigma_{\tau }}^{2}$$ (12)

Solving for the single-measurement error $\sigma_{\tau }$ and evaluating the right-hand side of the equation (see Supplementary Text) yields

$$\begin{array}{c} \sigma_{\tau }=\frac{1}{\varepsilon }\frac{\sqrt{e^{4\sigma^{2}\tau^{2}}-\varepsilon^{2}{\text{cos}}^{2}\left[\left(\Delta \omega \right)\tau \right]}}{\left(\Delta \omega \right)\text{sin}\left[\left(\Delta \omega \right)\tau \right]+4\sigma^{2}\tau \text{cos}\left[\left(\Delta \omega \right)\tau \right]} \end{array}$$ (13)

In the limit of τ → 0, ε → 1, Eq. 13 becomes Eq. 3 with *N* = 1. We note that for ε < 1, the optimal Fisher information resides at τ=(π/2)/Δω rather than at τ = 0.

#### Experimental displacement extraction

To extract the corresponding displacement arising from a given interferometric measurement, we use maximum-likelihood estimation and a set of reference fringes. These reference fringes are measured by sweeping the interferometer through 4 μm of path-length difference (2 μm of trombone displacement) and recording the four coincidence and anticoincidence fringes. The measured fringes are normalized and fit to sinusoidal curves

$$P\left(x\right)=a+b\text{cos}\left(\frac{2\pi }{c}x-d\right)$$ (14)

We ignore the exponential envelope, which is wide compared to the period of an interference fringe. From this fitting function, we can extract the total power *a* + *b*, the fringe visibility |*b*/*a*|, the wavelength *c*, and the phase offset *d*. Unless otherwise noted, this fitting provides all the visibility values reported in this work, with the uncertainty obtained by propagating the fit errors for *a* and *b*.

Performing a fit for each combination of detector pairs, we produce a set of curves: $P_{\mathrm{AA}}\left(x\right)$, $P_{\mathrm{AB}}\left(x\right)$, $P_{\mathrm{BA}}\left(x\right)$, and $P_{\mathrm{BB}}\left(x\right)$. The use of four individual reference curves, as opposed to a single fringe based on the coincidence probability $P_{C}$, allows us to account for potential visibility and phase differences associated with experimental imperfections in the interferometer, improving extraction accuracy.

For a given displacement measurement, we measure four pairs of coincident detections: $N_{\mathrm{AA}}$, $N_{\mathrm{AB}}$, $N_{\mathrm{BA}}$, and $N_{\mathrm{BB}}$. From here, we construct the log-likelihood function

$$\begin{array}{c} \begin{array}{c} \ell \left(x,N_{\mathrm{AA}},N_{\mathrm{AB}},N_{\mathrm{BA}},N_{\mathrm{BB}}\right)\\ =-\left\{\frac{{\left[P_{AA}\left(x\right)-\frac{N_{AA}}{N_{tot}}\right]}^{2}}{P_{AA}\left(x\right)}+\cdots +\frac{{\left[P_{BB}\left(x\right)-\frac{N_{BB}}{N_{tot}}\right]}^{2}}{P_{BB}\left(x\right)}\right\}\\ N_{\mathrm{tot}}=N_{\mathrm{AA}}+N_{\mathrm{AB}}+N_{\mathrm{BA}}+N_{\mathrm{BB}} \end{array} \end{array}$$ (15)

This form assumes that each measurement is sampled from a Gaussian distribution, which is a good approximation for Poissonian statistics in the high-count regime. Last, the displacement $x^{*}$ is recovered by using Python to perform the numerical optimization

$$x^{*}={\text{ArgMax}}_{x}\ell \left(x,N_{\mathrm{AA}},N_{\mathrm{AB}},N_{\mathrm{BA}},N_{\mathrm{BB}}\right)$$ (16)

Theoretical error bars are produced via Eq. 8, with the four reference curves used to calculate the single-event Fisher information I. From here, the theoretical resolution is

$$\sigma_{x,\text{theory}}\equiv \frac{1}{\sqrt{N_{\text{tot}}}}\frac{1}{\sqrt{I}}$$ (17)

This calculation neglects the nonzero bandwidth of the photons due to the relative size of the frequency detuning used.

#### Modeling the effect of loss

For the energy-entangled state used in this study, loss in one arm of the interferometer (corresponding to a transmission η in, e.g., mode *b*; see Supplementary Text) is frequency dependent

$$\begin{array}{c} \frac{1}{\sqrt{2}}\left({|\omega_{1}\rangle }_{a}{|\omega_{2}\rangle }_{b}+{|\omega_{2}\rangle }_{a}{|\omega_{1}\rangle }_{b}\right)\\ \to \frac{1}{\sqrt{\eta_{\omega_{1}}+\eta_{\omega_{2}}}}\left(\sqrt{\eta_{\omega_{2}}}{|\omega_{1}\rangle }_{a}{|\omega_{2}\rangle }_{b}+\sqrt{\eta_{\omega_{1}}}{|\omega_{2}\rangle }_{a}{|\omega_{1}\rangle }_{b}\right) \end{array}$$ (18)

So long as $\eta_{\omega_{1}}=\eta_{\omega_{2}}=\eta >0$, the loss manifests as a global factor on the quantum state that reduces the coincident detection rate (see Supplementary Text); the visibility of the interference fringe is unaffected.

However, if the interferometer is subject to loss-independent optical noise, the visibility is affected. Let $C_{0}=C_{\mathrm{LD}}+C_{\mathrm{LI}}$ be the total number of coincident detections when η=1, with contributions from both loss-dependent detections $C_{\mathrm{LD}}$, which includes most accidentals, and loss-independent noise detections $C_{\mathrm{LI}}.$ The loss-dependent detections correspond to fringes with visibility $V_{\mathrm{LD}}$, and the loss-independent noise detections correspond to noise fringes with visibility $V_{\mathrm{LI}}$. We assume $V_{\mathrm{LI}}=0$ since the noise photons are independent of the interference process. We can rewrite the coincidence fringe $N_{c}$ as

$$N_{c}=C_{\mathrm{LD}}\left[\frac{1}{2}+\frac{1}{2}V_{LD}\text{cos}\left(\varphi \right)\right]+\frac{C_{\mathrm{LI}}}{2}$$ (19)

where φ is the interferometer phase. The net fringe visibility $V_{0}$ is

$$\begin{array}{cc} V_{0} & =\frac{\text{max}\left(N_{c}\right)-\text{min}\left(N_{c}\right)}{\text{max}\left(N_{c}\right)+\text{min}\left(N_{c}\right)}\\ & =\frac{C_{\mathrm{LD}}}{C_{\mathrm{LD}}+C_{\mathrm{LI}}}V_{\mathrm{LD}}\\ & =\frac{C_{\mathrm{LD}}}{C_{0}}V_{\mathrm{LD}} \end{array}$$ (20)

When η<1, only the number of non-noise detections is reduced: $C_{\mathrm{LD}}\to \left(C_{\mathrm{LD}}\right)\eta$. Eq. 20 therefore becomes

$$V\left(\eta \right)=\frac{\left(C_{\mathrm{LD}}\right)\eta }{\left(C_{\mathrm{LD}}\right)\eta +C_{\mathrm{LI}}}V_{\mathrm{LD}}$$ (21)

We can rewrite Eq. 21 in terms of the experimentally measured quantities $V_{0}$, $C_{0},$ η, and $C_{\mathrm{LI}}$ by substituting in $\left(C_{\mathrm{LD}}\right)V_{\mathrm{LD}}=\left(C_{0}\right)V_{0}$ (Eq. 20) and $C_{\mathrm{LD}}=C_{0}-C_{\mathrm{LI}}$

$$V\left(\eta \right)=\frac{\left(C_{0}\right)\eta }{\left(C_{0}-C_{\mathrm{LI}}\right)\eta +C_{\mathrm{LI}}}V_{0}$$ (22)

Fringe scans performed without imbalanced path loss yield $V_{0}$ and $C_{0}$. We directly measured η and $C_{\mathrm{LI}}$ when characterizing our apparatus (see Supplementary Text).

In contrast, loss in one arm of a classical interferometer will always affect the state

$$\frac{1}{\sqrt{2}}\left({|1\rangle }_{a}+{|1\rangle }_{b}\right)\to \frac{1}{\sqrt{1+\eta }}\left({|1\rangle }_{a}+\sqrt{\eta }{|1\rangle }_{b}\right)$$ (23)

assuming the loss occurs in mode *b*. This unbalanced state produces interference fringes with reduced visibility

$$V=\frac{2\sqrt{\eta }}{1+\eta }V_{0}$$ (24)

#### Modeling the effect of background

In a two-photon interferometer, optical background will introduce noise in the form of increased accidentals (beyond those resulting from SPDC and imperfect optics), assuming that the background light is continuously distributed and uncorrelated in time. Let $\langle S_{i}\rangle$ be the mean total single-detector events when our background source is set to the *i*th brightness setting and ΔT be the temporal width of the detector coincidence window. The total number of accidental coincident detections $A_{i}$ is then

$$A_{i}\approx {\langle S_{i}\rangle }^{2}\Delta T$$ (25)

If $A_{i}$ is the total number of accidental coincident detections corresponding to the *i*th brightness setting of the background source, the total measured coincidence fringe is

$$N_{c,i}=\frac{1}{2}\left\{C_{0}\left[1+V_{0}\text{cos}\left(\varphi \right)\right]+\left(A_{i}-A_{0}\right)\right\}$$ (26)

where $C_{0}$, $A_{0}$, and $V_{0}$ are the total number of coincident detections (with accidentals), accidentals, and interferometer visibility with no background, respectively, and φ is the interferometer phase. The corresponding interference visibility is then

$$\begin{array}{cc} V_{i} & =\frac{\text{max}\left(N_{c,i}\right)-\text{min}\left(N_{c,i}\right)}{\text{max}\left(N_{c,i}\right)+\text{min}\left(N_{c,i}\right)}\\ & =\frac{C_{0}}{C_{0}+\left(A_{i}-A_{0}\right)}V_{0} \end{array}$$ (27)

In a classical interferometer, the background will act linearly on the detected photon rate $N_{i}$

$$N_{i}=\frac{1}{2}\left\{\langle S_{0}\rangle \left[1\pm V_{0}\text{cos}\left(\varphi \right)\right]+\left(\langle S_{i}\rangle -\langle S_{0}\rangle \right)\right\}$$ (28)

where $\langle S_{0}\rangle$ is the mean total single-detector events in the presence of no background (i.e., the 0th brightness setting). The corresponding interference visibility is

$$V_{i}=\frac{\langle S_{0}\rangle }{\langle S_{i}\rangle }V_{0}$$ (29)

Equations 27 and 29 may be rewritten in terms of the background, quantified as the fraction of total singles (see Supplementary Text).

### Entanglement source

Our entanglement source features a double beam displacer configuration to avoid potential effects from birefringent focusing. Each wavelength involved in the SPDC process (532, 810, and 1550 nm) has its own beam displacer assembly featuring a half-wave plate placed between two identical calcite beam displacers. For the pump, the first beam displacer (BD1) laterally displaces the *H* component from the *V* component. The optic axis of the half-wave plate is rotated by 45° with respect to *H* such that the *H* and *V* components are swapped before transmission through the second beam displacer (BD2). BD2 is rotated 180° about the optical axis relative to BD1 such that the *H* (originally *V*) component is displaced laterally in the opposite direction of the displacement introduced by BD1. The calcite crystals are cut such that the final separation between the *H* and *V* components is 4.8 mm, distributed symmetrically about the initial beam (see Supplementary Text). The same process occurs in reverse for the 810 and 1550 nm wavelengths, where the spatially separated beams are recombined into a single spatial mode for each wavelength.

The two MgO:PPLN SPDC crystals are 20 mm long with a 7.4 μm poling period. These crystals are designed to down-convert ordinary-polarized 532-nm photons into a pair of collinear, ordinary-polarized photons at 810 and 1550 nm via Type-0 SPDC (o → o + o). With 1-mm square facets, they are mounted in a custom housing such that their centers are separated by 4.8 mm (see Supplementary Text), matching the beam displacers. An oven maintains the crystals’ temperature at ~130°C.

The 4-mm diameter beam from the 532-nm continuous-wave pump laser is focused via a 400-mm plano-convex lens for a ~65-μm waist at the SPDC crystal. The down-converted photons are recollimated with plano-convex lenses with focal lengths of 300 and 125 mm for the 810- and 1550-nm photons, respectively. The focal lengths of the fiber-coupling aspheric lenses (15.29 mm for 810 nm and 18.4 mm for 1550 nm) correspond to beam waists of ~82 and ~85 μm, respectively, at the down-conversion crystals. After down-conversion, a long-pass filter assembly removes residual 532-nm pump photons in the 810-nm arm, and a 12-nm bandwidth bandpass filter does the same in the 1550-nm arm.

After the 810- and 1550-nm recombination beam displacers, the generated photons are in the maximally entangled state Eq. 4. The phase φ is determined by the phase of the beam-displacer interferometer, and is inconsequential for the main interferometer experiment, corresponding to a static phase offset in the measured interference fringes. However, to produce a more ideal entangled state, we use a tiltable quarter-wave plate in the 1550-nm arm to minimize φ. To further adjust the state, we use a trio of waveplates—two quarter-wave plates and a half-wave plate—in each arm to rotate the polarization of the photons. These waveplates serve a dual purpose: to perform the rotations necessary for a quantum state tomography of the source and to provide correction for the unitary transformations applied by the collection fibers (see Supplementary Text). The Supplementary Text contains details regarding spectral, brightness, and heralding efficiency measurements for our source, as well as descriptions of our procedures for optimizing and characterizing the generated entanglement.

### Dual-wavelength interferometer

The PBSs and the 50:50 (nominally) non-PBS are custom cube beamsplitters coated for 810 and 1550 nm. For stability, these were epoxied directly to stainless steel 1-inch-diameter pedestal pillar posts. A detailed characterization of the beamsplitters used and their impact on the interference visibility is presented in the Supplementary Text. Aside from the achromatic half-wave plate, the remainder of the optics within the interferometer are gold-coated mirrors, a cost-effective option for obtaining relatively high reflectivity for both 1550 and 810 nm.

To reduce phase drift, the interferometer module is fully enclosed by plastic panels to minimize the effects of air flow in addition to being built atop a standard floating optics table. We also use commercially available thermally compensated stainless steel mounts for all kinematic tip-tilt mounts within the interferometer and the optics leading up to its input. These mounts are designed to have minimal thermally induced deflections; a temperature sensor within the enclosure measured an ambient temperature range of ~0.4°C during a typical 24-hour period. Elsewhere, stainless steel is used instead of aluminum where possible because of steel’s lower coefficient of thermal expansion, e.g., the kinematic mounts are mounted on 1-inch-diameter steel pedestal pillar posts clamped directly onto the optical table. A characterization of the interferometer drift is given in the Supplementary Text. The single-axis piezoelectric nanopositioning stage integrated into the optical trombone system (path *a*) is specified for 30-μm travel with a unidirectional repeatability of ±2 nm.

### Spatial and temporal mode overlap

We performed knife-edge scans to ensure that the 1550- and 810-nm spatial modes overlapped in the interferometer (see Supplementary Text) so that both wavelengths probe the same target. However, while the observed interference effect requires the detection of two photons, these photons do not need to be temporally overlapped, i.e., they need not arrive together. This is in stark contrast to conventional two-photon interference, in which, without a quantum eraser for temporal which-path information (*24*), the interference visibility scales with temporal mismatch as

$$V=e^{-2\sigma^{2}\tau^{2}}$$ (30)

In energy-entangled two-photon interference, the two photons technically interfere directly, but that effect is suppressed by many orders of magnitude because of the frequency difference between the photons; this is the exponential envelope in Eq. 2. The dominant effect is instead equivalent to two individual single-photon interferometers, each with a single photon interfering with itself. These interferometers are entangled with one another, which creates the observed beat note. The two interference processes only rely on the photon of each wavelength being coherent with itself; interference will be observed as long as the path-length difference of the interferometer is within a coherence length for each individual photon, determined by their individual bandwidths.

### End-to-end system calibration and performance

To prepare our apparatus for interferometric measurements, the source, interferometer, and detection modules undergo a six-step calibration protocol (see Supplementary Text) to optimize the entangled probe state and configure the interferometer for maximum measurement sensitivity. Typical system performance as a function of pumping power (after calibration) is reported in the Supplementary Text.

### Sample fabrication

We fabricated both the test and calibration samples using identical methodology. We used double-side polished C-plane sapphire wafers (76.2 mm diameter, 0.5 mm thickness) as the substrate (see Supplementary Text for wafer specifications). We prepared wafers with a basic solvent clean and applied parallel strips of Kapton tape (12.7 mm wide) across the wafer in one direction with a typical separation of ~12.7 mm. We applied force to the tape edges to maximize adhesion.

We then coated the prepared samples via electron-beam physical vapor deposition with a nickel target (99.995% purity). The deposition time for the nominal requested film thickness was determined automatically on the basis of precalibrated deposition rate data. After coating, we removed the Kapton tape, leaving strips of uncoated and coated regions with well-defined edges (see Supplementary Text).

We cleaved a small, coated piece from the test sample for inspection under an atomic force microscope to verify film smoothness and uniformity. We measured a film roughness of ~100 pm, and observed no gaps or island-like features.

### Sample mounting and alignment

We affix the sample under study to a kinematic tip and tilt mount and attach the stage to the sample positioning system (SPS) located in path *b* of the interferometer immediately after the PBS. The SPS features motorized horizontal translation with 25 mm of travel, a typical positioning accuracy of ±2.2 μm, and a maximum speed of 5 mm per second (manufacturer specifications). Vertical and longitudinal translations are achieved via manual micrometers with 25 mm of travel. The horizontal and vertical axes of the SPS are approximately orthogonal to the probe propagation direction. We then align the sample such that the sample surface is approximately normal to the probe by adjusting the tip and tilt of the sample mount while monitoring the signal from the sample targeting system. Once the sample is mounted and aligned, an automated protocol optimizes the interferometer phase for maximum measurement sensitivity. The Supplementary Text details the protocols for the sample alignment, interferometer phase optimization, and sample measurement.

## Supplementary Materials

### This PDF file includes:

Supplementary Text Figs. S1 to S18 Tables S1 to S6 References
