# Supplementary material for: Fast quantum interferometry at the nanometer and attosecond scales with energy-entangled photons
Source: Sci Adv. 2025 May 21;11(21):eadw4938. doi: 10.1126/sciadv.adw4938 (PMC13109947; doi:10.1126/sciadv.adw4938)
Supplement: Supplementary file 1 — Supplementary Text Figs. S1 to S18 Tables S1 to S6 References [file sciadv.adw4938_sm.pdf]

Science Advances
AAAS

Supplementary Materials for

Fast quantum interferometry at the nanometer and attosecond scales with energy-entangled photons

Colin P. Lualdi *et al.*

Corresponding author: Colin P. Lualdi, clualdi2@illinois.edu

Sci. Adv. **11**, eadw4938 (2025)
DOI: 10.1126/sciadv.adw4938

This PDF file includes:

Supplementary Text
Figs. S1 to S18
Tables S1 to S6
References

Supplementary text

Energy-entangled two-photon interference theory

Quantum theory

Treatments similar to the one provided in this section can be found in Refs. (*14*) and (*25*). In general, a photon which has some finite spectral bandwidth can be described as

$$|\psi\rangle = \int d\omega_1 f(\omega_1) a^\dagger(\omega_1)|0\rangle, \tag{S1}$$

where $f(\omega_1)$ describes the frequency spread of the photon. For a Gaussian spread,

$$f(\omega_1) = \frac{1}{(2\pi\sigma^2)^{1/4}} e^{-\frac{(\omega_1-\omega_1^0)^2}{4\sigma^2}}. \tag{S2}$$

Here, ω_1^0 is the central frequency of the photon, and the prefactor assures normalization of $|\psi\rangle$ (recalling that σ is the Gaussian half-bandwidth). A general pure two-photon state can be written as

$$|\psi\rangle = \int d\omega_1 d\omega_2 f(\omega_1,\omega_2) a^\dagger(\omega_1) a^\dagger(\omega_2)|0\rangle, \tag{S3}$$

where $f(\omega_1,\omega_2)$ now describes the spectral information of both photons, including any potential frequency entanglement. To see how this state propagates under two-photon interference, we begin with single photons of frequencies ω_1 and ω_2, respectively, in spatial modes 1 and 2 and follow the two creation operators $a_1^\dagger(\omega_1)$, $a_2^\dagger(\omega_2)$. The photons are incident on a 50:50 beamsplitter, such that

$$\begin{aligned} a_1^\dagger(\omega_1) &\rightarrow \frac{1}{\sqrt{2}}\left(a_1^\dagger(\omega_1) + i a_2^\dagger(\omega_1)\right) \\ a_2^\dagger(\omega_2) &\rightarrow \frac{1}{\sqrt{2}}\left(a_2^\dagger(\omega_2) + i a_1^\dagger(\omega_2)\right), \end{aligned} \tag{S4}$$

where we have utilized a convention in which the spatial modes after the beamsplitter correspond to the transmitted mode of the input state. Then,

$$\begin{aligned} a_1^\dagger(\omega_1) a_2^\dagger(\omega_2) &\rightarrow \frac{1}{2}\left(a_1^\dagger(\omega_1) + i a_2^\dagger(\omega_1)\right)\left(a_2^\dagger(\omega_2) + i a_1^\dagger(\omega_2)\right) \\ &= \frac{1}{2}\left[a_1^\dagger(\omega_1) a_2^\dagger(\omega_2) + i\left(a_1^\dagger(\omega_1) a_1^\dagger(\omega_2) + a_2^\dagger(\omega_1) a_2^\dagger(\omega_2)\right) - a_2^\dagger(\omega_1) a_1^\dagger(\omega_2)\right] \\ &= \frac{1}{2}\left[a_1^\dagger(\omega_1) a_2^\dagger(\omega_2) - a_1^\dagger(\omega_2) a_2^\dagger(\omega_1)\right], \end{aligned} \tag{S5}$$

where in the last line we have removed terms which will not contribute coincidences, and we have reordered the terms such that $a_1^\dagger$ is always first. Suppose we have a delay in spatial mode 1, such that

$$|\psi\rangle \to |\psi(\tau)\rangle = \int d\omega_1 d\omega_2 f(\omega_1,\omega_2) e^{i\omega_1\tau} a_1^\dagger(\omega_1) a_2^\dagger(\omega_2)|0\rangle. \tag{S6}$$

After the beamsplitter we are left with the state

$$|\psi(\tau)\rangle = \frac{1}{2}\int d\omega_1 d\omega_2 f(\omega_1,\omega_2) e^{i\omega_1\tau}\left[a_1^\dagger(\omega_1)a_2^\dagger(\omega_2) - a_1^\dagger(\omega_2)a_2^\dagger(\omega_1)\right]|0\rangle. \tag{S7}$$

The photons are assumed to be detected via a pair of detectors with flat frequency response:

$$\widehat{S} = \int d\omega d\omega' a_1^\dagger(\omega) a_2^\dagger(\omega')|0\rangle\langle 0| a_1(\omega) a_2(\omega'). \tag{S8}$$

This projects the photon in spatial mode 1 (2) onto the frequency ω (ω'). We note that this operator could include a frequency-dependent detector efficiency or a spectral filter by including some weighting function $\eta(\omega)$. Because of the properties of creation and annihilation operators,

$$\langle 0|a_1(\omega)a_2(\omega')a_1^\dagger(\omega_1)a_2^\dagger(\omega_2)|0\rangle = \delta(\omega-\omega_1)\delta(\omega'-\omega_2), \tag{S9}$$

and the probability of coincidence is then

$$\begin{aligned} P_C &= \langle\psi(\tau)|\hat{S}|\psi(\tau)\rangle \\ &= \frac{1}{4}\int d\omega_1 d\omega_2 d\omega_1^{@} d\omega_2^{@} d\omega d\omega' f(\omega_1,\omega_2) f^*\left(\omega_1^{@},\omega_2^{@}\right) e^{i\left(\omega_1-\omega_1^{@}\right)\tau} \\ &\times\left[\delta\left(\omega-\omega_1^{@}\right)\delta\left(\omega'-\omega_2^{@}\right)-\delta\left(\omega-\omega_2^{@}\right)\delta\left(\omega'-\omega_1^{@}\right)\right] \\ &\times\left[\delta(\omega-\omega_1)\delta(\omega'-\omega_2)-\delta(\omega-\omega_2)\delta(\omega'-\omega_1)\right], \end{aligned} \tag{S10}$$

where we have used $\omega^{@}$ to distinguish the frequencies of $\langle\psi(\tau)|$ and $|\psi(\tau)\rangle$. Distributing the two delta function products and computing the ω, ω' integrals, we are left with

$$\begin{aligned} P_C = \frac{1}{2}\int d\omega_1 d\omega_2 d\omega_1^{@} d\omega_2^{@} f(\omega_1,\omega_2) f^*\left(\omega_1^{@},\omega_2^{@}\right) e^{i\left(\omega_1-\omega_1^{@}\right)\tau} \\ \times\left[\delta\left(\omega_1-\omega_1^{@}\right)\delta\left(\omega_2-\omega_2^{@}\right)-\delta\left(\omega_1-\omega_2^{@}\right)\delta\left(\omega_2-\omega_1^{@}\right)\right]. \end{aligned} \tag{S11}$$

Doing the $\omega^{@}$ integrals,

$$P_C = \frac{1}{2}\int d\omega_1 d\omega_2 |f(\omega_1,\omega_2)|^2 - \frac{1}{2}\int d\omega_1 d\omega_2 f(\omega_1,\omega_2) f^*(\omega_2,\omega_1) e^{i(\omega_1-\omega_2)\tau}. \tag{S12}$$

Since for a normalized state we have

$$\int d\omega_1 d\omega_2 |f(\omega_1,\omega_2)|^2 = \langle\psi|\psi\rangle = 1, \tag{S13}$$

it then follows that

$$P_C = \frac{1}{2} - \frac{1}{2}\int d\omega_1 d\omega_2 f\left(\omega_1,\omega_2\right) f^*\left(\omega_2,\omega_1\right) e^{i(\omega_1-\omega_2)\tau}. \tag{S14}$$

For a pair of photons produced by continuous-wave SPDC with pump frequency ω_p, the perfect spectral correlations $\omega_1 + \omega_2 = \omega_p$ allow us to write the state $|\psi_{SPDC}\rangle$ as

$$\begin{aligned} |\psi_{SPDC}\rangle &= \frac{1}{\left(2\pi\sigma^2\right)^{1/4}} \int d\Omega\, e^{-\frac{\Omega^2}{4\sigma^2}} a_1^\dagger(\omega_1^0 + \Omega)\, a_2^\dagger(\omega_2^0 - \Omega)\left|0\right\rangle \\ &= \frac{1}{\left(2\pi\sigma^2\right)^{1/4}} \int d\omega_1\, d\omega_2\, e^{-\frac{(\omega_1-\omega_1^0)^2}{4\sigma^2}} a_1^\dagger(\omega_1)\, a_2^\dagger(\omega_2) \delta((\omega_1-\omega_1^0)+(\omega_2-\omega_2^0))\left|0\right\rangle, \end{aligned} \tag{S15}$$

where we have defined

$$\begin{aligned} \Omega &\equiv \omega_1 - \omega_1^0 \\ &= -(\omega_2 - \omega_2^0), \end{aligned} \tag{S16}$$

and the second form is used so that we may write the frequency spectrum as

$$f_{SPDC}(\omega_1,\omega_2) = \frac{1}{\left(2\pi\sigma^2\right)^{1/4}} e^{-\frac{(\omega_1-\omega_1^0)^2}{4\sigma^2}} \delta((\omega_1-\omega_1^0)+(\omega_2-\omega_2^0)). \tag{S17}$$

Plugging (S17) into (S14), we focus on the integral in the second term:

$$\begin{aligned} \int d\omega_1 d\omega_2 f_{SPDC}\left(\omega_1,\omega_2\right) f^*{}_{SPDC}\left(\omega_2,\omega_1\right) e^{i(\omega_1-\omega_2)\tau} &= \frac{1}{\left(2\pi\sigma^2\right)^{1/2}} \int d\omega_1 d\omega_2 e^{-\frac{\left(\omega_1-\omega_1^0\right)^2}{4\sigma^2}} \\ &\quad \times e^{-\frac{\left(\omega_2-\omega_1^0\right)^2}{4\sigma^2}} e^{i(\omega_1-\omega_2)\tau} \\ &\quad \times \delta((\omega_1-\omega_1^0)+(\omega_2-\omega_2^0)). \end{aligned} \tag{S18}$$

Utilizing the delta function to complete the ω_2 integral, we have

$$\frac{1}{\left(2\pi\sigma^2\right)^{1/2}} \int d\omega_1 e^{-\frac{\left(\omega_1-\omega_1^0\right)^2}{4\sigma^2}} e^{-\frac{\left(\omega_1-\omega_2^0\right)^2}{4\sigma^2}} e^{i\left(2\omega_1-\omega_1^0-\omega_2^0\right)\tau}. \tag{S19}$$

Focusing for a moment only on the exponents,

$$
\begin{aligned}
&\frac{-1}{4\sigma^2}\left[\left(\omega_1-\omega_1^0\right)^2+\left(\omega_1-\omega_2^0\right)^2\right]+i\left(2\omega_1-\omega_1^0-\omega_2^0\right)\tau \\
&=\frac{-1}{4\sigma^2}\left[2\left(\omega_1\right)^2-2\omega_1\omega_1^0+\left(\omega_1^0\right)^2-2\omega_1\omega_2^0+\left(\omega_2^0\right)^2\right]+i\left(2\omega_1-\omega_1^0-\omega_2^0\right)\tau \\
&=\frac{-1}{2\sigma^2}\left[\left(\omega_1\right)^2-\omega_1\left(\omega_1^0+\omega_2^0+4i\sigma^2\tau\right)\right]-\frac{1}{4\sigma^2}\left[\left(\omega_1^0\right)^2+\left(\omega_2^0\right)^2\right]-i\left(\omega_1^0+\omega_2^0\right)\tau \\
&=\frac{-1}{2\sigma^2}\left[\omega_1-\frac{1}{2}\left(\omega_1^0+\omega_2^0+4i\sigma^2\tau\right)\right]^2-\frac{1}{4\sigma^2}\left[\left(\omega_1^0\right)^2+\left(\omega_2^0\right)^2\right] \\
&+\frac{1}{8\sigma^2}\left(\omega_1^0+\omega_2^0+4i\sigma^2\tau\right)^2-i\left(\omega_2^0+\omega_1^0\right)\tau \\
&=\frac{-1}{2\sigma^2}\left[\omega_1-\frac{1}{2}\left(\omega_1^0+\omega_2^0+4i\sigma^2\tau\right)\right]^2-\frac{1}{8\sigma^2}\left[\omega_1^0-\omega_2^0\right]^2-2\sigma^2\tau^2.
\end{aligned}
\tag{S20}
$$

Defining $\Delta\omega \equiv \omega_1^0-\omega_2^0$, we finally have

$$
\begin{aligned}
\frac{1}{\left(2\pi\sigma^2\right)^{1/2}}\int d\omega_1 e^{-\frac{\left(\omega_1-\omega_1^0\right)^2}{4\sigma^2}} e^{-\frac{\left(\omega_1-\omega_2^0\right)^2}{4\sigma^2}} e^{i\left(2\omega_1-\omega_1^0-\omega_2^0\right)\tau} &= \frac{e^{-\frac{(\Delta\omega)^2}{8\sigma^2}} e^{-2\sigma^2\tau^2}}{\left(2\pi\sigma^2\right)^{1/2}}\int d\omega_1 e^{\frac{-1}{2\sigma^2}\left[\omega_1-\frac{1}{2}\left(\omega_1^0+\omega_2^0+4i\sigma^2\tau\right)\right]^2} \\
&= e^{-\frac{(\Delta\omega)^2}{8\sigma^2}} e^{-2\sigma^2\tau^2},
\end{aligned}
\tag{S21}
$$

where we have used the normalization of the spectral spread to replace the integral with $\left(2\pi\sigma^2\right)^{1/2}$. Thus,

$$
P_C=\frac{1}{2}\left[1-\beta e^{-2\sigma^2\tau^2}\right],
\tag{S22}
$$

where we have defined

$$
\beta \equiv e^{-\frac{(\Delta\omega)^2}{8\sigma^2}}.
\tag{S23}
$$

If the photons are energy entangled, we have, in terms of frequency,

$$
\begin{aligned}
f_{ent}\left(\omega_1,\omega_2\right) &= \frac{1}{\sqrt{1+\beta}}\frac{1}{\sqrt{2}}\left(f_{SPDC}\left(\omega_1,\omega_2\right)+f_{SPDC}\left(\omega_2,\omega_1\right)\right) \\
&= \frac{1}{\sqrt{1+\beta}}\frac{1}{\sqrt{2}}\frac{1}{\left(2\pi\sigma^2\right)^{1/4}}\left[e^{-\frac{\left(\omega_1-\omega_1^0\right)^2}{4\sigma^2}}+e^{-\frac{\left(\omega_2-\omega_1^0\right)^2}{4\sigma^2}}\right] \\
&\times\delta((\omega_1-\omega_1{}^0)+(\omega_2-\omega_2{}^0)).
\end{aligned}
\tag{S24}
$$

Going forward we will ignore the normalization factor $(1+\beta)^{-1}$, since $\beta \approx 0$ for experimental values of $\Delta\omega$, σ. Utilizing the facts that $f_{ent}(\omega_2,\omega_1) = f_{ent}(\omega_1,\omega_2)$ and $f_{ent}^{*}(\omega_1,\omega_2) = f_{ent}(\omega_1,\omega_2)$, we plug this into (S14), leaving us with the second-term integral

$$\begin{aligned}\int d\omega_1 d\omega_2 f_{ent}(\omega_1,\omega_2) f_{ent}^{*}(\omega_2,\omega_1) e^{i(\omega_1-\omega_2)\tau} &= \frac{1}{2}\int d\omega_1 d\omega_2 \\ &\times \left(f_{SPDC}(\omega_1,\omega_2) + f_{SPDC}(\omega_2,\omega_1)\right)^2 e^{i(\omega_1-\omega_2)\tau} \\ &= \frac{1}{2}\int d\omega_1 d\omega_2 \left(\left|f_{SPDC}(\omega_1,\omega_2)\right|^2\right. \\ &+ \left|f_{SPDC}(\omega_2,\omega_1)\right|^2 \\ &\left.+ 2 f_{SPDC}(\omega_1,\omega_2) f_{SPDC}(\omega_2,\omega_1)\right) e^{i(\omega_1-\omega_2)\tau}.\end{aligned} \tag{S25}$$

We can identify the integral $\int d\omega_1 d\omega_2 f_{SPDC}(\omega_1,\omega_2) f_{SPDC}(\omega_2,\omega_1) e^{i(\omega_1-\omega_2)\tau}$ as equivalent to the non-degenerate SPDC seen above in (S18), and so that term is equal to

$$\begin{aligned}&\beta e^{-2\sigma^2\tau^2}, \\ &\beta \equiv e^{-\frac{(\Delta\omega)^2}{8\sigma^2}}.\end{aligned} \tag{S26}$$

Looking at the first two terms of (S25), if we relabel ω_1 and ω_2 in the second term we see

$$\frac{1}{2}\int d\omega_1 d\omega_2 \left|f_{SPDC}(\omega_2,\omega_1)\right|^2 e^{i(\omega_1-\omega_2)\tau} \to \frac{1}{2}\int d\omega_1 d\omega_2 \left|f_{SPDC}(\omega_1,\omega_2)\right|^2 e^{-i(\omega_1-\omega_2)\tau}, \tag{S27}$$

such that we only need to compute

$$\begin{aligned}\frac{1}{2}\int d\omega_1 d\omega_2 \left|f_{SPDC}(\omega_1,\omega_2)\right|^2 e^{\pm i(\omega_1-\omega_2)\tau} &= \frac{1}{2}\frac{1}{\left(2\pi\sigma^2\right)^{1/2}}\int d\omega_1 d\omega_2 e^{-\frac{\left(\omega_1-\omega_1^0\right)^2}{4\sigma^2}} e^{-\frac{\left(\omega_2-\omega_2^0\right)^2}{4\sigma^2}} \\ &\times e^{\pm i(\omega_1-\omega_2)\tau}\delta((\omega_1-\omega_1^{0})+(\omega_2-\omega_2^{0})) \\ &= \frac{1}{2}\frac{1}{\left(2\pi\sigma^2\right)^{1/2}}\int d\omega_1 e^{-\frac{\left(\omega_1-\omega_1^0\right)^2}{2\sigma^2}} e^{\pm i\left(2\omega_1-\omega_1^0-\omega_2^0\right)\tau}.\end{aligned} \tag{S28}$$

Focusing on exponents as before, we have

$$-\frac{\left(\omega_1-\omega_1^0\right)^2}{2\sigma^2}\pm i\left(2\omega_1-\omega_1^0-\omega_2^0\right)\tau=-\frac{\left(\omega_1^2-2\omega_1\omega_1^0\mp 4i\omega_1\sigma^2\tau\right)}{2\sigma^2}-\frac{\left(\omega_1^0\right)^2}{2\sigma^2}\mp i\left(\omega_1^0+\omega_2^0\right)\tau$$
$$=-\frac{\left(\omega_1-\left(\omega_1^0\pm 2i\sigma^2\tau\right)\right)^2}{2\sigma^2}+\frac{1}{2\sigma^2}\left(\omega_1^0\pm 2i\sigma^2\tau\right)^2$$
$$-\frac{\left(\omega_1^0\right)^2}{2\sigma^2}\mp i\left(\omega_1^0+\omega_2^0\right)\tau \tag{S29}$$
$$=-\frac{\left(\omega_1-\left(\omega_1^0\pm 2i\sigma^2\tau\right)\right)^2}{2\sigma^2}-2\sigma^2\tau^2\pm i\left(\Delta\omega\right)\tau,$$

and so

$$\frac{1}{2}\frac{1}{\left(2\pi\sigma^2\right)^{1/2}}\int d\omega_1 e^{-\frac{\left(\omega_1-\omega_1^0\right)^2}{2\sigma^2}}e^{\pm i\left(2\omega_1-\omega_1^0-\omega_2^0\right)\tau}=\frac{1}{2}\frac{1}{\left(2\pi\sigma^2\right)^{1/2}}e^{-2\sigma^2\tau^2}e^{\pm i(\Delta\omega)\tau}$$
$$\times\int d\omega_1 e^{-\frac{\left(\omega_1-\left(\omega_1^0\pm 2i\sigma^2\tau\right)\right)^2}{2\sigma^2}} \tag{S30}$$
$$=\frac{1}{2}e^{-2\sigma^2\tau^2}e^{\pm i(\Delta\omega)\tau}.$$

We then have,

$$\frac{1}{2}\int d\omega_1 d\omega_2\left(\left|f_1\left(\omega_1,\omega_2\right)\right|^2+\left|f_1\left(\omega_2,\omega_1\right)\right|^2\right)e^{i\left(\omega_1-\omega_2\right)\tau}=\frac{e^{-2\sigma^2\tau^2}}{2}\left(e^{i(\Delta\omega)\tau}+e^{-i(\Delta\omega)\tau}\right)$$
$$=\cos\left(\left(\Delta\omega\right)\tau\right)e^{-2\sigma^2\tau^2}. \tag{S31}$$

Combining everything at the end,

$$P_C=\frac{1}{2}\left[1-\cos\left(\left(\Delta\omega\right)\tau\right)e^{-2\sigma^2\tau^2}-\beta e^{-2\sigma^2\tau^2}\right], \tag{S32}$$

where $\beta=e^{-\frac{(\Delta\omega)^2}{8\sigma^2}}$ as before. Finally, taking the limit $\beta\to 0$, we are left with Eq. 2 in the main text:

$$P_C=\frac{1}{2}\left[1-\cos\left(\left(\Delta\omega\right)\tau\right)e^{-2\sigma^2\tau^2}\right]. \tag{S33}$$

Quantum Fisher information

In evaluating the quantum Fisher information (Eq. 6) for the case of energy-entangled two-photon interference, the probe state $\left|\psi\left(\tau\right)\right\rangle$ (the state after acquiring the interferometric phase shift $\omega\tau$ but before reaching the final interfering beamsplitter) is (using the second form of (S15))

$$
\begin{aligned}
|\psi\rangle = \frac{1}{\sqrt{2(1+\beta)}} \int d\Omega f(\Omega) \Big[& e^{-i(\omega_1^0+\Omega)\tau} a_1^\dagger(\omega_1^0+\Omega) a_2^\dagger(\omega_2^0-\Omega) \\
& + e^{-i(\omega_2^0+\Omega)\tau} a_1^\dagger(\omega_2^0+\Omega) a_2^\dagger(\omega_1^0-\Omega) \Big] |0\rangle,
\end{aligned} \tag{S34}
$$

where the two photons have center frequencies ω_1^0, ω_2^0, $\beta = e^{-\frac{(\omega_1^0-\omega_2^0)^2}{8\sigma^2}}$ fixes the normalization of the entangled state, Ω is defined as in (S16), and

$$
f(\Omega) = e^{-\frac{\Omega^2}{4\sigma^2}} \tag{S35}
$$

is the normalized SPDC spectral amplitude. Note that we have used $f(-\Omega) = f(\Omega)$ to change the variable of integration $\Omega \to -\Omega$ in the second term. The first term in Eq. 6 requires

$$
\begin{aligned}
\left| \frac{\partial \psi(\tau)}{\partial \tau} \right\rangle = \frac{-i}{\sqrt{2(1+\beta)}} \int d\Omega f(\Omega) \Big[& (\omega_1^0+\Omega) e^{-i(\omega_1^0+\Omega)\tau} a_1^\dagger(\omega_1^0+\Omega) a_2^\dagger(\omega_2^0-\Omega) \\
& + (\omega_2^0+\Omega) e^{-i(\omega_2^0+\Omega)\tau} a_1^\dagger(\omega_2^0+\Omega) a_2^\dagger(\omega_1^0-\Omega) \Big],
\end{aligned} \tag{S36}
$$

and gives

$$
\begin{aligned}
\left\langle \frac{\partial \psi(\tau)}{\partial \tau} \middle| \frac{\partial \psi(\tau)}{\partial \tau} \right\rangle &= \frac{1}{2(1+\beta)} \int d\Omega d\Omega' f(\Omega) f(\Omega') [\kappa_1 + \kappa_2 + \kappa_3 + \kappa_4] \\
&= \frac{1}{2} \left((\omega_1^0)^2 + (\omega_2^0)^2 + 2\sigma^2 \right),
\end{aligned} \tag{S37}
$$

where

$$
\begin{aligned}
\kappa_1 \equiv & (\omega_1^0+\Omega')(\omega_1^0+\Omega) e^{i(\omega_1^0+\Omega')\tau} e^{-i(\omega_1^0+\Omega)\tau} \\
& \times a_1(\omega_1^0+\Omega') a_2(\omega_2^0-\Omega') a_1^\dagger(\omega_1^0+\Omega) a_2^\dagger(\omega_2^0-\Omega) \\
\kappa_2 \equiv & (\omega_1^0+\Omega')(\omega_2^0+\Omega) e^{i(\omega_1^0+\Omega')\tau} e^{-i(\omega_2^0+\Omega)\tau} \\
& \times a_1(\omega_1^0+\Omega') a_2(\omega_2^0-\Omega') a_1^\dagger(\omega_2^0+\Omega) a_2^\dagger(\omega_1^0-\Omega) \\
\kappa_3 \equiv & (\omega_2^0+\Omega')(\omega_1^0+\Omega) e^{i(\omega_2^0+\Omega')\tau} e^{-i(\omega_1^0+\Omega)\tau} \\
& \times a_1(\omega_2^0+\Omega') a_2(\omega_1^0-\Omega') a_1^\dagger(\omega_1^0+\Omega) a_2^\dagger(\omega_2^0-\Omega) \\
\kappa_4 \equiv & (\omega_2^0+\Omega')(\omega_2^0+\Omega) e^{i(\omega_2^0+\Omega')\tau} e^{-i(\omega_2^0+\Omega)\tau} \\
& \times a_1(\omega_2^0+\Omega') a_2(\omega_1^0-\Omega') a_1^\dagger(\omega_2^0+\Omega) a_2^\dagger(\omega_1^0-\Omega),
\end{aligned} \tag{S38}
$$

and we have used the facts that

$$\int d\Omega \left|f(\Omega)\right|^2 \Omega = 0$$
$$\int d\Omega \left|f(\Omega)\right|^2 \Omega^2 = \sigma^2. \tag{S39}$$

Looking at the second term in Eq. 6, we have

$$\begin{aligned}
\left\langle \psi(\tau) \middle| \frac{\partial \psi(\tau)}{\partial \tau} \right\rangle &= \frac{1}{2(1+\beta)} \int d\Omega d\Omega' f(\Omega) f(\Omega') \\
&\quad \Big[\left(\omega_1^0 + \Omega\right) e^{i\left(\omega_1^0 + \Omega'\right)\tau} e^{-i\left(\omega_1^0 + \Omega\right)\tau} \delta\left(\Omega - \Omega'\right) \\
&\quad + \left(\omega_2^0 + \Omega\right) e^{i\left(\omega_1^0 + \Omega'\right)\tau} e^{-i\left(\omega_2^0 + \Omega\right)\tau} \delta\left(\left(\omega_1^0 + \Omega'\right) - \left(\omega_2^0 + \Omega\right)\right) \\
&\quad + \left(\omega_1^0 + \Omega\right) e^{i\left(\omega_2^0 + \Omega'\right)\tau} e^{-i\left(\omega_1^0 + \Omega\right)\tau} \delta\left(\left(\omega_2^0 + \Omega'\right) - \left(\omega_1^0 + \Omega\right)\right) \\
&\quad + \left(\omega_2^0 + \Omega\right) e^{i\left(\omega_2^0 + \Omega'\right)\tau} e^{-i\left(\omega_2^0 + \Omega\right)\tau} \delta\left(\Omega - \Omega'\right) \Big] \\
&= \frac{1}{2}\left(\omega_1^0 + \omega_2^0\right),
\end{aligned} \tag{S40}$$

and it therefore follows that

$$\left| \left\langle \psi(\tau) \middle| \frac{\partial \psi(\tau)}{\partial \tau} \right\rangle \right|^2 = \frac{1}{4}\left(\left(\omega_1^0\right)^2 + 2\omega_1^0\omega_2^0 + \left(\omega_2^0\right)^2\right). \tag{S41}$$

The quantum Fisher information Q can then be calculated as

$$\begin{aligned}
\frac{Q}{4} &= \left\langle \frac{\partial \psi(\tau)}{\partial \tau} \middle| \frac{\partial \psi(\tau)}{\partial \tau} \right\rangle - \left| \left\langle \psi(\tau) \middle| \frac{\partial \psi(\tau)}{\partial \tau} \right\rangle \right|^2 \\
&= \frac{1}{2}\left(\left(\omega_1^0\right)^2 + \left(\omega_2^0\right)^2 + 2\sigma^2\right) - \frac{1}{4}\left(\left(\omega_1^0\right)^2 + \left(\omega_2^0\right)^2 + 2\omega_1^0\omega_2^0\right) \\
&= \frac{1}{4}\left[\left(\omega_1^0\right)^2 - 2\omega_1^0\omega_2^0 + \left(\omega_2^0\right)^2\right] + \sigma^2 \\
&= \frac{\left(\Delta\omega\right)^2}{4} + \sigma^2.
\end{aligned} \tag{S42}$$

Classical Fisher information

For ideal energy-entangled two-photon interference, Eq. 8 becomes

$$\begin{aligned}\mathcal{I} &= \frac{\left(\partial P_C / \partial \tau\right)^2}{P_C} + \frac{\left(\partial P_A / \partial \tau\right)^2}{P_A} \\ &= \frac{\left(\partial P_C / \partial \tau\right)^2}{P_C} + \frac{\left(\partial (1-P_C) / \partial \tau\right)^2}{1-P_C} \\ &= \frac{\left(\partial P_C / \partial \tau\right)^2}{P_C(1-P_C)}.\end{aligned} \tag{S43}$$

We then calculate the single-event Fisher information $\mathcal{I}$:

$$\begin{aligned}\frac{\partial P_C}{\partial \tau} &= \frac{1}{2} e^{-2\sigma^2\tau^2} \left((\Delta\omega)\sin\left((\Delta\omega)\tau\right) + 4\sigma^2\tau\cos\left((\Delta\omega)\tau\right)\right) \\ P_C(1-P_C) &= \frac{1}{4}\left(1-\cos^2\left((\Delta\omega)\tau\right)e^{-4\sigma^2\tau^2}\right),\end{aligned} \tag{S44}$$

and so

$$\begin{aligned}\mathcal{I} &= \frac{\left(e^{-2\tau^2\sigma^2}\left((\Delta\omega)\sin\left((\Delta\omega)\tau\right) + 4\sigma^2\tau\cos\left((\Delta\omega)\tau\right)\right)\right)^2}{1-\cos^2\left((\Delta\omega)\tau\right)e^{-4\sigma^2\tau^2}} \\ &= \frac{\left((\Delta\omega)\sin\left((\Delta\omega)\tau\right) + 4\sigma^2\tau\cos\left((\Delta\omega)\tau\right)\right)^2}{e^{4\sigma^2\tau^2} - \cos^2\left((\Delta\omega)\tau\right)}.\end{aligned} \tag{S45}$$

Experimental saturation of the Cramér-Rao bound

A mixed energy-entangled (EE) state

$$\begin{aligned}\rho_{EE,mixed}(\varepsilon) &= \frac{1+\varepsilon}{2}\left|\Psi^+\right\rangle\left\langle\Psi^+\right| + \frac{1-\varepsilon}{2}\left|\Psi^-\right\rangle\left\langle\Psi^-\right| \\ \left|\Psi^+\right\rangle &\equiv \frac{1}{\sqrt{2}}\left(|\,\omega_1\rangle_a\,|\,\omega_2\rangle_b + |\,\omega_2\rangle_a\,|\,\omega_1\rangle_b\right) \\ \left|\Psi^-\right\rangle &\equiv \frac{1}{\sqrt{2}}\left(|\,\omega_1\rangle_a\,|\,\omega_2\rangle_b - |\,\omega_2\rangle_a\,|\,\omega_1\rangle_b\right)\end{aligned} \tag{S46}$$

has purity $\left(1+\varepsilon^2\right)/2$ and produces interference fringes

$$P_C(\tau) = \frac{1+\varepsilon}{2} \times \frac{1}{2}\left(1 - \cos\left((\Delta\omega)\tau\right)e^{-2\sigma^2\tau^2}\right) + \frac{1-\varepsilon}{2} \times \frac{1}{2}\left(1 + \cos\left((\Delta\omega)\tau\right)e^{-2\sigma^2\tau^2}\right)$$
$$= \frac{1}{2}\left(1 - \varepsilon\cos\left((\Delta\omega)\tau\right)e^{-2\sigma^2\tau^2}\right). \quad \text{(S47)}$$

Using error propagation, the variance of P_C is equal to

$$\mathrm{Var}\left[P_C\right] = \left(\frac{\partial P_C}{\partial \tau}\right)^2 \sigma_\tau{}^2, \quad \text{(S48)}$$

and so the single-measurement error σ_τ is given by

$$\begin{aligned} \sigma_\tau &= \frac{\sqrt{\mathrm{Var}\left[P_C\right]}}{\left|\frac{\partial P_C}{\partial \tau}\right|} \\ &= \frac{\sqrt{E\left[P_C^2\right] - E\left[P_C\right]^2}}{\left|\frac{\partial P_C}{\partial \tau}\right|} \\ &= \frac{\sqrt{E\left[P_C\right] - E\left[P_C\right]^2}}{\left|\frac{\partial P_C}{\partial \tau}\right|}, \end{aligned} \quad \text{(S49)}$$

where $E\left[P_C\right]$ is the expectation value of P_C, and in the last line we have used the fact that $E\left[P_C\right] = E\left[P_C^2\right]$ since P_C is a projective measurement. We note that this form is similar to the classical Fisher information described above. In this case,

$$\begin{aligned} \mathrm{Var}\left[P_C\right] &= \frac{1}{4}\left(1 - \varepsilon^2\cos^2\left((\Delta\omega)\tau\right)e^{-4\sigma^2\tau^2}\right) \\ \left|\frac{\partial P_C}{\partial \tau}\right| &= \frac{\varepsilon}{2}e^{-2\sigma^2\tau^2}\left((\Delta\omega)\sin\left((\Delta\omega)\tau\right) + 4\sigma^2\tau\cos\left((\Delta\omega)\tau\right)\right) \end{aligned} \quad \text{(S50)}$$

and so

$$\sigma_\tau = \frac{\sqrt{\mathrm{Var}[P_C]}}{\left|\frac{\partial P_C}{\partial \tau}\right|}$$

$$= \frac{\frac{1}{2}\sqrt{1-\varepsilon^2 \cos^2\left((\Delta\omega)\tau\right) e^{-4\sigma^2\tau^2}}}{\frac{\varepsilon}{2} e^{-2\tau^2\sigma^2}\left((\Delta\omega)\sin\left((\Delta\omega)\tau\right) + 4\sigma^2\tau\cos\left((\Delta\omega)\tau\right)\right)} \tag{S51}$$

$$= \frac{1}{\varepsilon}\frac{\sqrt{e^{4\sigma^2\tau^2} - \varepsilon^2\cos^2\left((\Delta\omega)\tau\right)}}{(\Delta\omega)\sin\left((\Delta\omega)\tau\right) + 4\sigma^2\tau\cos\left((\Delta\omega)\tau\right)}.$$

Modeling the effect of loss

Fig. S1A shows the induced system transmission η tested in our experiment as a function of half-wave plate angle, indicating a minimum observed transmission of $\eta = 5(1)\times 10^{-4}$. The experimental transmission is obtained for each waveplate angle θ by calculating the relative coincidence rate $\eta(\theta) \equiv N_c(\theta)/N_c(0)$, where the coincidence rate $N_c(\theta)$ has been corrected to account for accidental coincident detections and erroneous events from leakage.

As noted in the main text, in the case of the two-photon interferometer, loss reduces the coincident detection rate. In some circumstances, a reduced detection rate may be compensated for by increasing the integration time. For example, when measuring the visibilities shown in Fig. 4A, we progressively increased the per-point integration time for the fringe scans above the default 1 s (up to 10 s) as loss increased to maintain sufficient counts. However, we still see a reduction in fit quality for the interference fringes, leading to unreliable visibility extraction. The quantum visibilities shown in Fig. 4A are therefore instead calculated according to

$$V_\eta \equiv \frac{\max_{\delta x}(P_c) - \min_{\delta x}(P_c)}{\max_{\delta x}(P_c) + \min_{\delta x}(P_c)}, \tag{S52}$$

where the extrema are taken from the P_C fringe scan performed at a given transmission η. Error bars are estimated via error propagation assuming Poissonian photon statistics.

Modeling the effect of background

The total number of accidental coincident detections A_i may be written in terms of R_i, the ratio of mean total single-detector events in the presence of no background, $\langle S_0 \rangle$, and the mean total single-detector events coming from the incident background, $\langle S_{BG,i} \rangle$:

$$
\begin{aligned}
A_i &\approx \langle S_i\rangle^2 \Delta T \\
&= \left(\langle S_0\rangle + \langle S_{BG,i}\rangle\right)^2 \Delta T \\
&= A_0 + \left(2\langle S_0\rangle\langle S_{BG,i}\rangle + \langle S_{BG,i}\rangle^2\right)\Delta T \\
&= A_0\left(1+2R_i+R_i^2\right), \\
R_i &\equiv \frac{\langle S_{BG,i}\rangle}{\langle S_0\rangle}.
\end{aligned} \tag{S53}
$$

For quantum interference measurements, the single-detector events are summed across four detectors, while for classical interference measurements they are summed across two detectors. Eq. 27 can then be rewritten as

$$
V_i = \frac{1}{1+(2R_i+R_i^2)\dfrac{A_0}{C_0}} V_0. \tag{S54}
$$

The background is quantified as the fraction of total singles, defined as

$$
\begin{aligned}
B_i &= \frac{\langle S_i\rangle - \langle S_0\rangle}{\langle S_i\rangle} = \frac{\langle S_{BG,i}\rangle}{\langle S_i\rangle} \\
\rightarrow R_i &= \frac{\langle S_i\rangle - \langle S_0\rangle}{\langle S_0\rangle} = \frac{B_i}{B_i-1},
\end{aligned} \tag{S55}
$$

where B_i is background corresponding to the background light source being set to the ith brightness setting. Substituting this into (S54),

$$
V_i = \frac{1}{1+\dfrac{(3B_i-2)B_i}{(B_i-1)^2}\dfrac{A_0}{C_0}} V_0. \tag{S56}
$$

The corresponding result for classical interference is

$$
V_i = (1-B_i)V_0. \tag{S57}
$$

The effect of the introduced background on single and coincident detection rates are shown in Fig. S1B.

Materials and methods

Source module: Design

Figure S2 shows a schematic of the entanglement source. The half-wave plate and quarter-wave plate immediately after the input coupler are calibrated to maximize transmission through a 532-nm polarizing beamsplitter such that the pump polarization is $|H\rangle_{532}$. Next, a

second HWP in a motorized rotation mount is used to rotate the pump polarization to approximately $\left|D\right\rangle_{532} = \left(\left|H\right\rangle_{532} + \left|V\right\rangle_{532}\right)/\sqrt{2}$, though the exact pump polarization is adjusted to balance the resulting entangled state (see the **Polarization-entangled state balancing** step in the **End-to-end system calibration protocol** section).

The underlying beam-displacer interferometer is detailed in Fig. S3A. The calcite beam displacers transmit vertically polarized light, and laterally displace horizontally polarized light by ~2.4 mm. The beam displacers are cut to different lengths for each wavelength to achieve the same lateral displacement: 21.6 mm for 532 nm, 22.5 mm for 810 nm, and 23.9 mm for 1550 nm.

Figure S3B shows the custom housing in which the MgO:PPLN SPDC crystals are mounted. The crystals are configured to produce the down-conversion

$$\begin{aligned} \left|H\right\rangle_{532} &\rightarrow \left|H\right\rangle_{810}\left|H\right\rangle_{1550}, \\ \left|V\right\rangle_{532} &\rightarrow \left|V\right\rangle_{810}\left|V\right\rangle_{1550}. \end{aligned} \tag{S58}$$

Source module: Characterization

Figure S4 shows the measured pump (532 nm) and signal (810 nm) spectra from our source. Gaussian fits yield center wavelengths of 531.9120(5) nm and 810.504(1) nm for the pump and signal spectra, respectively, as well as bandwidths of 0.167(1) nm and 0.495(2) nm. Energy conservation implies an idler wavelength of 1547.484(5) nm, which is close to the nominal wavelength of 1550 nm.

We observed a mean brightness of 91,649(1,429) and 85,980(1,298) detected pairs per second per mW for the $\left|H\right\rangle_{1550}\left|H\right\rangle_{810}$ and $\left|V\right\rangle_{1550}\left|V\right\rangle_{810}$ processes, respectively (the numbers in parentheses are the standard deviations). For this measurement, the overall pumping power was set to 1 mW and the polarization of the pump beam incident on the first beam displacer was set to $\left|D\right\rangle$. The pump beam for each process was blocked one at a time to observe the contribution of the other process to the overall detected pair rate. The fiber compensation half-wave and quarter-wave plates were set to arbitrary known angles (the zero angles of their rotation mounts) and the tomography quarter-wave plates were set to their calibrated zero angles (where the waveplate axes are aligned with the *HV* basis). Since the detectors have polarization-dependent efficiency and the two processes involve orthogonal polarizations, the pair detection rate was manually maximized for each process by adjusting the polarization control paddles attached to the fibers leading to the 1550 and 810-nm detectors. The same detectors from port *A* of the interferometer were used for this measurement, and were connected directly to the source output fibers, bypassing the interferometer module. We recorded 50 consecutive detector measurements with a 1-second integration time per measurement. While the precise brightness varies depending on the source configuration (i.e., the pumping power split between each process, the angle settings of the output waveplates, etc.), the sum of the per-process brightnesses indicates a detected source brightness of $>10^5$ detected pairs per second per mW.

During the same measurement, we also obtained the mean net Klyshko heralding efficiencies (*26*) for both wavelengths for both processes. For $\left|H\right\rangle_{1550}\left|H\right\rangle_{810}$, we observed 8.7(1)% and 19.9(2)% for 1550 and 810 nm, respectively. For $\left|V\right\rangle_{1550}\left|V\right\rangle_{810}$, we observed

9.2(1)% and 20.8(1) % for 1550 and 810 nm, respectively. The given uncertainties are the standard deviations. These net efficiencies do not include corrections for fiber-coupling losses, detector efficiency and background, or accidentals (detections of uncorrelated photon pairs). The detector efficiencies are summarized in Table S1, and the background and accidentals were negligible relative to the observed detection rates. Correcting for the fiber link transmissions and detector efficiencies (but not the fiber coupling loss), we obtain estimated lower bounds of ~10% and ~22% for 1550 and 810 nm, respectively, for $|H\rangle_{1550}|H\rangle_{810}$. The corresponding values for $|V\rangle_{1550}|V\rangle_{810}$ are ~10% and 23%.

The **End-to-end system calibration protocol** section describes the procedure for calibrating our source to optimize the generated entangled state, and the **State tomography** subsection details our procedure for characterizing the entanglement. The resulting state density matrix is shown in Fig. S5. We observed a purity, concurrence, and singlet fraction of 90.3(2)%, 89.6(2)%, and 94.8(1)%, respectively.

Interferometer module: General details

Table S2 summarizes the end-to-end free-space transmission of the interferometer module.

Figure S6 shows a typical drift in P_C over 100 seconds. Also shown is the normalized noise, defined as the variance in P_C over Poissonian variance λ, with a 10-s rolling window. Over 100 seconds our interferometer exhibits a mean 10-second normalized noise of 1.3(6) with passive stabilization and no thermal isolation. This result illustrates how our basic passive measures are largely sufficient, given our ability to perform measurements with nanometer-scale resolution on a timescale much faster than the interferometer drift.

For details on how we reconfigured our interferometer to obtain the classical interference data discussed in this work, please refer to the **Classical frequency beating** section.

Interferometer module: Interference visibility

The interference visibility achievable with our interferometer is largely determined by the purity of the polarization entangled state, the extinction ratio of both output ports of the polarizing beamsplitter (PBS) at the interferometer input, and the splitting ratio of the non-polarizing beamsplitter (NPBS) at the interferometer output.

The state purity is discussed in the main text. We characterized the PBS extinction ratio in situ with classical alignment lasers nominally at 1550 and 810 nm and power meters. A calibrated polarizer was used to set the incident polarization to either horizontal or vertical, and the transmission for both output ports was measured for both input polarizations (correcting for background on the power meters). All measurements were performed in free space. From these we obtain T_p:T_s and R_s:R_p of ~7,100 and ~500 for 1550 nm, respectively. The corresponding values for 810 nm are ~5,600 and ~500.

Similarly, we characterized the transmission and reflectance of the NPBS with classical alignment lasers, for both the transmitted (i.e., through the PBS) and reflected (i.e., off the PBS) paths of the interferometer (modes *a* and *b*, respectively). The polarization of the light in the reflected path was rotated to match that of the transmitted path by an achromatic half-wave plate (775-1550 nm). All measurements were performed in free space. For the transmitted path, we

observed ~62% and ~36% transmission and reflectance, respectively, for 1550 nm. The corresponding values for 810 nm are ~50% and ~50%. For the reflected path, we observed ~61% and ~36% for 1550 nm, and ~50% and ~49% for 810 nm.

In Fig. S7 we plot the effects of imperfect PBS extinction ratio and NPBS splitting efficiency on the final fringe visibility using a simplified numerical model. The effect of an imperfect PBS, shown in Fig. S7A, is modeled by taking the PBS as a polarization-dependent beamsplitter with transmission and reflection coefficients given by

$$\begin{aligned} T_p &= \frac{ER_T\left(1-ER_R\right)}{1-ER_T ER_R} \quad ; \quad R_p = \frac{1-ER_T}{1-ER_T ER_R} \\ T_s &= \frac{1-ER_R}{1-ER_T ER_R} \quad ; \quad R_s = \frac{ER_R\left(1-ER_R\right)}{1-ER_T ER_R}, \end{aligned} \tag{S59}$$

where ER_T (ER_R) is the extinction ratio (ER) of the transmitted (reflected) port of the PBS. For a perfect PBS, ER_T , $ER_R \to \infty$, such that $T_p, R_s \to 1$, $T_s, R_p \to 0$, as expected. In contrast, for a completely non-polarizing beamsplitter, ER_T , $ER_R \to 1$, such that $T_s, T_p, R_s, R_p \to 0.5$. The effect of an imperfect PBS is to probabilistically route the polarization-entangled state into the incorrect interferometer mode, leading to “leakage” events in which both photons are routed into the same path. This can be seen by observing the state of the $\left|H\right\rangle_{1550}\left|V\right\rangle_{810}$ process after the (first) PBS and half-wave plate in the interferometer,

$$\begin{aligned} \left|H\right\rangle_{1550}\left|V\right\rangle_{810} \to & \sqrt{T_{p,1550}T_{s,810}}\left|H,a\right\rangle_{1550}\left|V,a\right\rangle_{810} \\ & + e^{-i\omega_{810}\tau}\sqrt{T_{p,1550}R_{s,810}}\left|H,a\right\rangle_{1550}\left|H,b\right\rangle_{810} \\ & + e^{-i\omega_{1550}\tau}\sqrt{R_{p,1550}T_{s,810}}\left|V,b\right\rangle_{1550}\left|V,a\right\rangle_{810} \\ & + e^{-i(\omega_{1550}+\omega_{810})\tau}\sqrt{R_{p,1550}R_{s,810}}\left|V,b\right\rangle_{1550}\left|H,b\right\rangle_{810}, \end{aligned} \tag{S60}$$

with a similar equation holding for the $\left|V\right\rangle_{1550}\left|H\right\rangle_{810}$ process; here relative phase is introduced in mode *b*. We end up with four distinguishable interference processes corresponding to the four combinations of photon polarization. The $|H\rangle|H\rangle$ and $|V\rangle|V\rangle$ terms (terms 2 and 3 in Eq. (S60), respectively) will lead to interference at the beat-note frequency $\Delta\omega$, with their visibilities dependent on the PBS extinction ratio. Similarly, the $|H\rangle|V\rangle$ and $|V\rangle|H\rangle$ terms (terms 1 and 4 in (S60), respectively) will lead to interference at the sum frequency $\omega_1+\omega_2$.

The net fringe can then be used to calculate the resulting interference visibility, shown in Fig. S7A. We have fixed the transmission port extinction ratio (ER) T_p:T_s at 10,000, and varied the reflected port ER R_s:R_p, for both the case where a single PBS is used, or, as in our experiment, where a second PBS is used in the reflected port to provide additional filtering. In both cases, we see that for ERs > 100, the visibility loss is negligible. In our experiment, where we both have a second filtering PBS and a reflected port ER of ~500, this effect can be safely ignored.

Fig. S7B plots the fringe visibility as a function of NPBS splitting ratio for a single wavelength, assuming the other is fixed at 50:50. To model this effect, we replace the two beamsplitters in (S4) with unbalanced transmission and reflection probabilities

$$
\begin{aligned}
a_1^\dagger(\omega_1) &\to \frac{1}{\sqrt{T_{\omega_1}+R_{\omega_1}}}\left(\sqrt{T_{\omega_1}}\,a_1^\dagger(\omega_1)+i\sqrt{R_{\omega_1}}\,a_2^\dagger(\omega_1)\right) \\
a_2^\dagger(\omega_2) &\to \frac{1}{\sqrt{T_{\omega_2}+R_{\omega_2}}}\left(\sqrt{T_{\omega_2}}\,a_2^\dagger(\omega_2)+i\sqrt{R_{\omega_2}}\,a_1^\dagger(\omega_2)\right).
\end{aligned} \tag{S61}
$$

This changes the coincidence fringes to

$$
P_c = \frac{1}{\left(T_{\omega_1}+R_{\omega_1}\right)\left(T_{\omega_2}+R_{\omega_2}\right)}\left(T_{\omega_1}T_{\omega_2}+R_{\omega_1}R_{\omega_2}-2\sqrt{T_{\omega_1}T_{\omega_2}R_{\omega_1}R_{\omega_2}}\cos\left((\Delta\omega)\tau\right)e^{-2\sigma^2\tau^2}\right). \tag{S62}
$$

We note that when $T_{\omega_1}=R_{\omega_1}=T_{\omega_2}=R_{\omega_2}\to 0.5$, $P_c\to$ (S33), as expected. The visibility can then be calculated from the resulting fringes, substituting experimental values for the beamsplitter ratios. With a splitting ratio of ~63:37 – corresponding to the ~61% transmission and ~36% reflection probabilities associated with the 1550-nm NPBS – we predict a fringe visibility of ~97%.

Relatedly, the four coincident detection fringes have fitted visibilities that vary slightly with respect to each other: 88.2(4)%, 89.2(5)%, 87.7(4)%, and 89.2(5)% for 1550*A*-810*B*, 1550*B*-810*A*, 1550*A*-810*A*, and 1550*B*-810*B*, respectively. We suspect this variation rises from small differences in optical alignment between the four output modes of the interferometer. The four single-detection fringes all have fitted visibilities less than 1%: 0.9(2)%, 0.6(2)%, 0.8(2)%, and 0.9(2)% for 1550*A*, 1550*B*, 810*A*, and 810*B*, respectively.

Detection module

The four fiber couplers in the detection module couple photons exiting the interferometer into single-mode fibers (SMF-28 for 1550 nm and 780HP for 810 nm) with anti-reflection-coated tips. The coupling efficiencies are summarized in Table S3.

Each 2-m collection fiber is mated to a 20-m transfer fiber that connects to the fiber input of a superconducting nanowire single-photon detector (SNSPD). Each transfer fiber is equipped with a three-paddle polarization controller to optimize the polarization of light incident on the detectors, which have polarization-dependent efficiencies. Key detector specifications are summarized in Table S1.

Table S4 summarizes the combined timing jitter for coincident detections. The observed jitter led us to select 50 ps as the radius for the coincident detection window. Unless otherwise noted, the default integration time for all measurements is 1 second.

Spatial mode overlap

At the interferometer input, the 1550-nm and 810-nm light are launched from fiber into free space and multiplexed into a common spatial mode via a dichroic mirror. We verify the spatial mode overlap by performing knife-edge scans with classical light from nominally 1550-nm and 810-nm alignment lasers. A dichroic mirror downstream of the knife edge directs each wavelength into its own power sensor for power measurement. Knife-edge scans were performed transversely to the common mode in both the x (horizontal) and y (vertical) directions at two longitudinal points ($z = 0$ mm and $z = 45$ mm). We assume Gaussian beams and fit the resulting normalized power versus knife-edge displacement curves to the function

$$P(\delta)=\frac{P_0}{2}\left[1\pm\mathrm{Erf}\left(\frac{\sqrt{2}\,(\delta-\delta_0)}{w}\right)\right], \tag{S63}$$

where P is the measured normalized power, δ is the position displacement of the knife edge in the scan direction, and P_0 is the normalized power incident on the knife edge. δ_0 and w are the centroid and $1/e^2$ radius of the beam's transverse intensity profile, respectively. The sign of the error function is determined by the scan direction relative to the beam.

From the fit parameters we obtain $\Delta \equiv \delta_0^{810}-\delta_0^{1550}$. As shown in Fig. S8, the observed Δ values all are zero within fitting error, indicating that the 1550-nm and 810-nm beams are well-overlapped and parallel to each other. Averaging the w values for the four scans per wavelength gives a mean w of 1.47(1) mm for 1550 nm and 1.59(1) mm for 810 nm, indicating that the beams are *x-y* symmetric and well-collimated. The values in parentheses are the standard deviations.

End-to-end system calibration protocol

To prepare our experiment for nanometer-scale measurements, we perform an end-to-end system calibration to achieve optimal performance. The six-step procedure is as follows:

1. **Transfer fiber compensation:** Stress-induced birefringence in the two single-mode fibers linking the source and interferometer modules apply an arbitrary two-qubit polarization rotation on the state generated by the source module (Eq. 4). To facilitate subsequent operations, we use compensation half-wave and quarter-wave plates to correct for the fiber transformation such that the waveplates-fiber system for each wavelength acts as the identity. Our procedure is as follows:
    a. The pump driving the $|H\rangle_{1550}|H\rangle_{810}$ process in the source ("Path *A*") is blocked such that the source produces only photon pairs in the state $|V\rangle_{1550}|V\rangle_{810}$.
    b. The reflected path of the interferometer (mode *b*) is blocked such that only horizontally polarized photons that transmit through the first polarizing beamsplitter are detected in the detection module.
    c. The half-wave and quarter-wave plates inserted in front of both the 1550-nm and 810-nm output fiber couplers in the source module are rotated to minimize the number of photon detection events on all four detectors. A search algorithm based on the principles of gradient descent is utilized, and each wavelength is optimized separately. Once the counts are minimized, the combined waveplates-fiber system for each wavelength acts as the identity, transforming the input state $|V\rangle$ to the output state $|V\rangle$, which reflect off the polarizing beamsplitter into the blocked reflected path of the interferometer, and are therefore not detected.

    Finally, to verify the compensation, both half-wave plates are rotated by 45° to maximize detector counts. The ratio of the observed maximum and minimum counts is then taken to obtain the extinction ratio. Then, all blocked paths are unblocked while the waveplates are left at their optimized angles, all of which are redefined as 0° to simplify subsequent calibration steps.

The described process is fully automated via motorized rotation mounts. The runtime depends on the initial conditions. A typical calibration ran for 14 minutes and yielded background-corrected extinction ratios of 1,845(102) for 1550 nm and 900(13) for 810 nm. The given uncertainties assume Poissonian statistics for detector counts.

2. **Polarization-entangled state balancing:** We realize the optimal polarization-entangled state when the $|H\rangle_{1550}|H\rangle_{810}$ and $|V\rangle_{1550}|V\rangle_{810}$ terms in Eq. 4 have equal amplitudes upon incidence on the polarizing beamsplitter at interferometer input. The amplitudes may be unequal because of unbalanced pumping powers or heralding efficiencies for each of the two SPDC processes in the source. To equalize the amplitudes, we perform the following procedure:
   a. The reflected path of the interferometer (mode *b*) is blocked such that only horizontally polarized photons that transmit through the input polarizing beamsplitter are detected in the detection module.
   b. The half-wave plates for each transfer fiber are rotated by 45° to project the entangled state onto $|V\rangle_{1550}|V\rangle_{810}$ at the first polarizing beamsplitter in the interferometer.
   c. The half-wave plates are then rotated back to 0° to project the state onto $|H\rangle_{1550}|H\rangle_{810}$.
   d. We compute the difference in the observed photon pair detection rates from the two projections in steps *b* and *c*. If the difference is less than 2% of the rate for $|H\rangle_{1550}|H\rangle_{810}$, the two terms are considered balanced, and the optimization ends.
   e. If the difference is equal to or greater than 2%, the optimization continues. The half-wave plate in the source that controls the polarization of the 532-nm pump incident on the first beam displacer is rotated by 0.2°, with the rotation direction dependent on the sign of the difference. Rotating this half-wave plate changes the relative pair production rate for the $|H\rangle_{1550}|H\rangle_{810}$ and $|V\rangle_{1550}|V\rangle_{810}$ processes in the source, and thereby their relative amplitudes in the entangled state.
   f. Steps *b*, *c*, and *d* are then repeated. If the difference falls below the 2% threshold, the optimization ends.
   g. Steps *e* and *f* are repeated however many times are necessary to achieve the 2% threshold.

The described process is fully automated via motorized rotation mounts. The runtime depends on the initial state of the source. A typical calibration ran for 7 minutes and returned 20.5° as the optimized angle, with 89,773 counts for $|H\rangle_{1550}|H\rangle_{810}$ and 90,017 counts for $|V\rangle_{1550}|V\rangle_{810}$ (5-second integration time). The optimized angle for the pump half-wave plate is typically a few degrees away from the nominal 22.5° angle (where both processes are pumped equally). For example, when the half-wave plate is at 20.5°, the pumping power is split 57:43 between the $|H\rangle_{1550}|H\rangle_{810}$ and $|V\rangle_{1550}|V\rangle_{810}$ processes, respectively. Pumping one process harder than the other compensates for asymmetric loss between the processes such that after all losses are factored in the state amplitudes become balanced.

3. **State tomography:** The next step is to verify the entangled state via a two-qubit state tomography. With the transfer fibers calibrated to act as the identity (Step 1), we form the state projectors for the tomography by rotating the compensation half-wave plate and a second quarter-wave plate immediately preceding the compensation waveplates. The compensation quarter-wave plate remains fixed at its optimized angle from Step 1. After traveling through the transfer fibers, photons from the source are projected onto $|H\rangle$ by transmitting through the first polarizing beamsplitter at the interferometer input (with the reflected port blocked). These photons then continue to the detection module for detection.

   To form the state projectors a second quarter-wave plate is necessary since the two state rotations realized with a half-wave and a quarter-wave plate (one equatorial and one azimuthal on the Poincaré sphere) can only transform an arbitrary polarization state to a linear state or vice versa. To transform an arbitrary polarization state to another arbitrary polarization state, it is necessary to add a second azimuthal rotation with a second quarter-wave plate. We perform three rotations (one azimuthal, one equatorial, and another azimuthal) using a quarter-wave plate, a half-wave plate, and a quarter-wave plate, in that order. With the transfer fibers between the projecting beamsplitter and the source, the beamsplitter may be interpreted (from the source side) as projecting the source photons into an arbitrary polarization basis state. With three waveplates on the source side, we can therefore transform this arbitrary projection to another arbitrary projection, namely, onto each of the six "canonical" polarization basis states required for a complete state tomography:

   1. Horizontal: $|H\rangle$
   2. Vertical: $|V\rangle$
   3. Diagonal: $|D\rangle$
   4. Anti-diagonal: $|A\rangle$
   5. Left circular: $|L\rangle$
   6. Right circular: $|R\rangle$

   Prior to installation, the first quarter-wave plate is calibrated against the polarizing beamsplitter (without intervening transfer fibers) to identify the angle where its fast axis is parallel to $|H\rangle$ as defined by the beamsplitter. This angle and the optimized angles for the half-wave plate and second quarter-wave plate from Step 1 are taken as the new "zero" angles for the tomography.

   Table S5 summarizes the angles corresponding to projection onto each basis state for each waveplate, relative to their zero angles. These angles transform the projector to $|H\rangle$ since our physical projector is a polarizing beamsplitter in the *HV* basis, e.g., $|D\rangle$ at the source is transformed into $|H\rangle$ at the polarizing beamsplitter.

   A tomography is taken by projecting the entangled state produced by the source onto all 36 possible two-qubit permutations of the six polarization basis states above. For

each projection, the total number of coincident detections across all four coincident detection channels and the corresponding accidental counts are recorded. The coincident counts are corrected by subtracting the accidentals counts, with an enforced lower bound of zero counts. The resulting counts are then processed by a maximum likelihood estimation analysis (*27*) to recover the entangled state density matrix ρ most likely to give rise to these measurement outcomes (Fig. S5). From this matrix we compute the state purity

$$\gamma \equiv tr\left(\rho^2\right), \tag{S64}$$

and the concurrence

$$\mathcal{C} \equiv \max\left(0, \lambda_1 - \lambda_2 - \lambda_3 - \lambda_4\right), \tag{S65}$$

where $\lambda_{(1,2,3,4)}$ are the eigenvalues of the matrix

$$\sqrt{\sqrt{\rho}\tilde{\rho}\sqrt{\rho}}, \tag{S66}$$

where $\tilde{\rho}$ is defined as

$$\tilde{\rho} \equiv \left(\sigma_y \otimes \sigma_y\right)\rho^*\left(\sigma_y \otimes \sigma_y\right), \tag{S67}$$

where σ_y is the Pauli-Y matrix. We also compute the singlet fraction

$$SF \equiv \max_U \left\langle \Psi^- \left| U^\dagger \rho U \right| \Psi^- \right\rangle \tag{S68}$$

for the unitary transformation *U*. The singlet fraction can be interpreted either as the fidelity between ρ and the nearest maximally entangled state, or as the maximal fidelity between ρ and the polarization Bell state $\left|\Psi^-\right\rangle = \left(\left|HV\right\rangle - \left|VH\right\rangle\right)/\sqrt{2}$, assuming we have local unitary transformation *U* to correct ρ. Fig. S5 shows the density matrix ρ_{SF} produced by this second interpretation, though optimized to achieve the equivalent state $\left|\Psi^+\right\rangle = \left(\left|HV\right\rangle + \left|VH\right\rangle\right)/\sqrt{2}$ for visual clarity.

The tomography-measurement process is fully automated via motorized rotation mounts and takes 6 minutes to perform. The process is performed 10 times and the mean purity, concurrence, and singlet fraction are calculated, with the standard deviation taken as the uncertainty. The density matrices (Figs. 1B and S5) are generated by taking the mean of the real and imaginary elements of the 10 individual density matrices obtained from the tomographies.

4. **Bit flip:** To convert the polarization entangled state generated by our source, $\left|\varphi_{SPDC}\right\rangle$ (Eq. 4), to the desired energy entangled state (Eq. 1), the state incident on the first polarizing beamsplitter in the interferometer must be of the form

$$I_{1550}X_{810}\left|\varphi_{SPDC}\right\rangle = \frac{1}{\sqrt{2}}\left(\left|H\right\rangle_{1550}\left|V\right\rangle_{810} + e^{i\varphi}\left|V\right\rangle_{1550}\left|H\right\rangle_{810}\right), \tag{S69}$$

where I and X are the identity and Pauli-X gates, respectively. We perform a bit flip on the 810-nm photon by rotating the compensation half-wave plate in the 810-nm side of the source by 45° (relative to the calibrated angle from Step 1).

5. **Interferometer path-length balancing:** To maximize interference visibility, the effective lengths of the two optical paths of the interferometer must be equal (assuming other degrees of freedom are indistinguishable). During initial interferometer construction, the path lengths were made as equal as possible with the use of a ruler. This was followed by sending 810-nm laser pulses with $O(10^1)$-ps FWHM pulse duration through the interferometer (with roughly equal powers in both paths) and monitoring the optical power in free space at one of the interferometer outputs while scanning the relative path lengths with the optical trombone. With the laser pulses having a short coherence length, high-visibility classical interference fringes are observed only when the path lengths are close to being equalized.

   Fine optimization takes place during the system end-to-end calibration. The trombone scans ±1 mm around the initial position in 0.2 mm steps, using the servo motor. At each step, the sinusoidal quantum interference fringe is measured by scanning the nano-positioning stage over 1.9 µm in 100-nm steps and recording the resulting coincident detection fringes. After extracting the fitted visibility from each fringe scan, the visibility versus trombone servo motor position dataset is then fitted to

$$V(x) = V_0 e^{-2\sigma^2\left(\frac{x-x_0}{c}\right)^2}, \tag{S70}$$

   where V_0 is the maximum quantum interference visibility, σ is the photons' half bandwidth (angular frequency), x_0 is the trombone servo motor position corresponding to V_0, and c is the speed of light. The trombone is then moved to position x_0 and the visibility re-measured to verify consistency with V_0. At this point, the interferometer is balanced up to the nano-positioner position; the trombone servo motor has a guaranteed bi-directional repeatability of ±1 µm, well below the 30-µm travel of the nano-positioner. The fully automated procedure takes 7 minutes. A typical optimization returned a balanced visibility of 89.7(6)%, in agreement with the 90(1)% predicted from the fit.

   This balancing procedure was used to obtain the data shown in Fig. 1D, but with a different step size for the servo motor, and the position data redefined from the trombone motor position to the relative change in optical path length. The given uncertainty is from the fitting error. A selection of the interference fringes from this measurement is shown in Fig. S9. We observe interference fringes over millimeters of relative delay with the fringe visibility decreasing when moving away from the zero-delay position, consistent with Eq. 2.

6. **Detector balancing:** The four superconducting nanowire single-photon detectors in the detection module have polarization-dependent detection efficiencies, in addition to small variations in their intrinsic efficiencies as well as fiber coupling and transmission losses.

   While imbalanced detector efficiencies will not change the visibility of any given fringe (assuming the counts remain well above the background), the maximum-likelihood estimation phase extraction can be affected if the four interference fringes N_{AA}, N_{AB},

N_{BA}, and N_{BB} have different efficiencies that are not properly accounted for. This can be done by measuring the relative efficiency of each fringe for incorporation into the interference model, or by equalizing the system detection efficiency.

We equalize the four detectors by leveraging their polarization-dependent efficiencies. First, the trombone position is moved a few millimeters away from the optimized position (from Step 5) such that the count rates for all detectors are unaffected by interference. Then, the count rates of the two 1550-nm detectors are equalized and maximized by manually adjusting the three-paddle fiber polarization controllers attached to the detectors' input fibers. The same procedure is performed for the two 810-nm detectors. Upon completion, the trombone position is returned to the optimized position.

With the conclusion of Step 6, the system is now fully calibrated and ready for measurements.

System performance

Figure S10 illustrates the performance of the calibrated system as a function of the source pumping power. While the individual detector count rates scale linearly with the pump power, the total coincident detection rate saturates slightly above 3 mW of pumping power. We attribute this to our tight coincidence window (±50 ps). Accordingly, for most measurements we set 3 mW as the upper bound for the pumping power. We observe a coincidence to accidental ratio (CAR) of 1044(153) at 1 mW of pumping power, which falls to 310(16) at 3 mW. We define the CAR as

$$CAR = \frac{C - A}{A}, \tag{S71}$$

where C is the number of coincident detections and A is the number of accidentals. We measure accidentals by recording the number of coincident detections at an arbitrary time delay between individual channels and leaving unchanged the coincident detection window.

Accidentals are uncorrelated photon pairs. They can arise from the fact that entangled photon sources based on spontaneous parametric down-conversion have some probability of generating multiple entangled pairs in the same temporal mode. If the opposite members of each pair are lost, a coincident detection may still occur with the two remaining photons; however, these photons are not entangled with each other and contribute to unwanted background noise. In our case, the CAR is an important consideration as coincidences scale linearly with pumping power while accidentals scale quadratically. Consequently, for certain measurements where the probe experiences high loss, increasing the pumping power to compensate for a reduced coincident detection rate may not be the optimal strategy, as the accidentals may start to dominate and degrade the interference visibility; increasing the integration time instead may be more effective.

Lastly, we observe that the interference visibility is largely independent of the pumping power, indicating that increasing the pumping power does not significantly affect the entangled state produced by the source for up to at least 4 mW. We also observe that the measurement resolution (estimated from the interference fringes) scales as the inverse square root of the pumping power, as expected given that the number of coincident detections N scales linearly with the pumping power (at least through 3 mW) and Poissonian uncertainty scales as $1/\sqrt{N}$.

Note on error propagation

In this work, the uncertainties for some derived quantities are estimated via error propagation and are indicated as such. For a derived quantity $f(x, y, \cdots)$ based on the values of $x, y, \cdots$ and their corresponding errors $\sigma_x, \sigma_y, \cdots$, we obtain σ_f (the resulting error for f) via the following formula:

$$\sigma_f = \sqrt{\left(\frac{\partial f}{\partial x}\right)^2 (\sigma_x)^2 + \left(\frac{\partial f}{\partial y}\right)^2 (\sigma_y)^2 + \cdots}. \tag{S72}$$

In doing so, we assume that the variables $x, y, \cdots$ are uncorrelated.

Sample measurements

Sample measurement protocol

After the sample is inserted into the Sample Positioning System (SPS), we activate the Sample Targeting System (STS) by flipping into place a mirror flipper at the interferometer input, which allows a collimated beam from a red Helium-Neon (HeNe) laser to enter the interferometer along approximately the same path as the energy-entangled probe photons (as marked by iris diaphragms) and illuminate the sample. After using the SPS to optimize the sample's horizontal and vertical positions based on this visible indicator of the probe beam location, we adjust the sample tip and tilt to overlap back-reflected (off the sample) HeNe light with the forward-propagating beam. This ensures that the sample surface is approximately normal to the probe beam. Once the sample is positioned and aligned, the STS is deactivated and its mirror flipped out of the way. Note that the STS as described is not suitable for photosensitive samples; alternative sample positioning techniques may be used instead.

We then illuminate the sample with energy-entangled probe photons. As the presence of the sample changes the relative lengths of the two interferometer paths, we re-balance these lengths to maximize the interference visibility using the same procedure described in the **End-to-end system calibration protocol** section to maximize the interference visibility. Then, reference interference fringes (see the **Experimental displacement extraction** section in the main text) are obtained at the initial sample position by keeping the sample fixed and sweeping the interferometer through 4 μm of path-length difference (2-μm trombone displacement) and recording the two coincidence and two anti-coincidence fringes in 0.1 μm steps (0.05 μm trombone steps).

A search protocol then automatically recenters the coincident detection probability at $P_C \approx 0.5$, where $dP_C/d\tau$ is maximized. Depending on the amount of delay introduced by the sample, it is sometimes advantageous to shift the starting P_C slightly off-center to remain close to the high-sensitivity region of the fringe (e.g., a sample measurement where P_C ranges from 0.4 through 0.6 may return better results than one where P_C ranges from 0.5 through 0.7). While our current methods limit the total measurable displacement to half of a fringe (corresponding to

scanning P_C from 0 to 1), larger displacements can be measured by keeping track of the total number of fringes passed through ("fringe counting") as well as the position within a fringe.

Finally, we translate the sample transversely to the probe beam (horizontally) and record the counts from the four coincident detection channels as a function of the sample's transverse position. We then compare these recorded counts against the reference fringes via the procedure described in the **Experimental displacement extraction** section (main text) to extract the interferometer displacement as a function of sample position, which may be fitted to extract useful quantities (e.g., see the **Model for thin-film sample measurements** section).

Since the thin-film sample measurements performed as a part of this work involved scanning the probe beam across a sharp boundary between the uncoated and coated regions of a substrate (in either direction), the resulting data is similar to those obtained via the knife-edge scans described in the **Spatial mode overlap** section. Accordingly, we estimate the quantum probe transmission through the thin film by fitting the total coincident detection counts versus sample position data with a modified form of (S63), and take the fitting error as the uncertainty. Examples of such an analysis are shown in Figs. 5A (top panel) and S14.

When selecting samples for study, it is important to keep in mind that transmissive measurements with energy-entangled photons function best when probing samples featuring roughly comparable transmissions for the two frequencies involved.

Thin-film sample fabrication

Table S6 summarizes the specifications for the wafers used as substrates for our samples. Fig. S11 illustrates how our sample fabrication process yields strips of uncoated and coated regions with well-defined edges.

Model for thin-film sample measurements

With a probe beam diameter on the order of millimeters, data acquired during sample measurements will be a convolution of the probe and sample features. Additionally, a large probe diameter necessitates scanning over a distance on the order of millimeters to obtain a zero-thickness reference (uncoated region) and a relative thickness measurement (coated region). In scanning over this long of a distance, our measurement will be influenced by any effective curvature that may be present in the substrate (e.g., due to local variations in substrate thickness).

To extract the film thickness from our measurement given these considerations, we developed an analysis model. In our model, we treat our target feature as an infinitely sharp step in height atop a substrate with a linear wedge and quadratic curvature (Fig. S12A) and consider a Gaussian optical probe (Fig. S12B). The convolution of the two is shown in Fig. S12C, and has the following form (assuming an effective refractive index of 2 for the sample and substrate and 1 for the ambient atmosphere):

$$\sigma_x(y) = a + b(y - y_0) + c\left((y - y_0)^2 + \sigma^2\right) - \frac{a}{2}\operatorname{Erfc}\left(\frac{y - y_0}{\sigma\sqrt{2}}\right) + d. \tag{S73}$$

Here, σ_x is the displacement measured by our interferometer, y is the sample position (transverse to probe beam propagation), y_0 is the step edge position, a is the film thickness, b is the linear wedge, c is the quadratic curvature, σ is the standard deviation of the Gaussian transverse

intensity profile of the probe beam, Erfc is the complementary error function, and d is the vertical offset. By fitting the displacement data with (S73), we can obtain values for the film thickness as well as the linear and quadratic curvatures from the fit parameters a, b, and c.

Converting the fitted σ to $1/e^2$ diameter, we can check for general agreement with the physical probe beam diameter, which can be measured via knife-edge scans using a procedure largely identical to the one described in the **Spatial mode overlap** section. However, instead of using classical alignment lasers and power meters, we use single photons from our entanglement source, which are coupled into single-mode fiber at the interferometer output and detected with single-photon detectors. The resulting counts versus knife-edge displacement data (Fig. S13) therefore provides information regarding the spatial modes relevant to interference measurements. We also perform the knife-edge scan at only one longitudinal location. We observe a mean diameter of 1.21(4) mm over all eight scans (horizontal and vertical for both ports A and B for both 810 and 1550 nm). Based on this diameter, the 2.4(2) mm fitted $1/e^2$ diameter for the 5-nm test sample is reasonable. We note that this comparison is limited as the fitted waist depends on the actual steepness of the step edge. However, the fitted quantity of interest – the step height – is independent of the beam waist in this analysis.

Refractive index: Calibration sample

The effective thickness x_0 of our thin film sample (as measured by our interferometer) is related to the physical thickness x via the relation

$$x = \frac{x_0}{n_{film} - 1}, \tag{S74}$$

where n_{film} is the refractive index of the film material. The denominator being $n-1$ rather than n arises from the fact that the film is replacing the air that the optical probe would have otherwise propagated through. The *net* difference in path length introduced by the film when inserted in the interferometer (i.e., the effective thickness) is therefore $x_0 = xn_{film} - xn_{air}$. Taking $n_{air} = 1$ and rearranging, we recover (S74).

For best results, we utilize experimentally measured values of n_{film} for both the quantum and classical sample measurements. The uncertainty in the measured n_{film} can be combined with the fitting error for x_0 via error propagation to obtain the uncertainty in x. Obtaining such experimental values requires a calibration sample with a well-defined thickness. We designed a calibration sample by considering the nature of our test sample and its fabrication process.

In general, electron-beam physical vapor deposition can reliably produce films of the desired thickness for sufficiently thick films (e.g., tens of nanometers or greater). This accuracy is realized by calibrating the electron-beam machine by depositing a few hundred nanometers of film at a particular power. The deposition time is noted, and the film thickness is measured via X-ray reflectometry, which generally gives accurate results for films with >10-nm thickness. Combining the deposition time and thickness yields a deposition rate; the electron-beam machine uses this rate to time depositions at a particular power to achieve the desired film thickness. However, for the specific machine used to support this work, ramping up to the target power takes some time, during which time the deposition rate is not well-defined. For very thin films with thickness on the order of nanometers, like our test sample, the deposition occurs over the

ramp-up period. The resulting thickness is therefore less accurate, as evidenced by the fact that our test sample has a nominal thickness of 5 nm but a measured thickness of ~7 nm.

Accordingly, we fabricated a calibration sample with 50-nm thickness using identical materials and methodology as the test sample. We selected 50-nm thickness as it is a thickness that our electron-beam machine can reliably deliver. Spot measurements with an atomic force microscope confirmed a film thickness of ~50 nm. The next two sections describe how we used this calibration sample to obtain n_{film} for the quantum and classical probes.

Refractive index: Quantum probe

To obtain n_{film} for the quantum probe, we measured the effective thickness of the calibration sample in our interferometer using the same procedure as with the 5-nm sample (described in the main text). With a 50-nm film the quantum probe transmission is 1.6(2)%, yet going from the uncoated to the coated regions the quantum interference visibility decreases only moderately, from 88.1(5)% to 71(2)%. This moderate drop in visibility may be corrected for by measuring $V_{P_C}(y)$, the P_C fringe visibility at sample position *y*, for every value of *y* in the sample scan. Then, for each sample position, the visibilities of the four coincident detection reference fringes (measured at sample position $y=0$) are scaled by $V_{P_C}(y)/V_{P_C}(y=0)$.

These visibility-corrected reference fringes are then used to extract the interferometer displacement as a function of sample position via the procedure described in the **Experimental displacement extraction** section (main text). To simplify subsequent analysis steps, the displacement was extracted in units of phase, i.e., $k\sigma_z(y)$ where *k* is the *k*-vector of the quantum probe and $\sigma_x(y)$ is the interferometer displacement as a function of sample position. We fit the resulting phase data to a modified version of (S73):

$$k\sigma_x(y)=k\left(n_f a+n_s\left(b(y-y_0)+c\left((y-y_0)^2+\sigma^2\right)\right)-n_f\frac{a}{2}\mathrm{Erfc}\left(\frac{y-y_0}{\sigma\sqrt{2}}\right)+n_s d\right). \quad \text{(S75)}$$

In this version, the equation is multiplied by the quantum *k*-vector *k*, the terms relating to the film are multiplied by n_f, and the terms relating to the substrate are multiplied by n_s. Here, n_f and n_s are defined as

$$\begin{aligned} n_f &\equiv n_{film}-1 \\ n_s &\equiv n_{substrate}-1, \end{aligned} \quad \text{(S76)}$$

and are used to convert physical displacement to interferometer displacement (see (S74)). $n_{substrate}$ is the refractive index of sample substrate. For this fit, the parameter of interest is n_f, from which we can extract n_{film}. We fix the film thickness *a* to the calibrated value of 50 nm and assume nominal wavelengths for *k* such that

$$k\equiv k_{810}-k_{1550}\equiv\frac{2\pi}{810\text{ nm}}-\frac{2\pi}{1550\text{ nm}}. \quad \text{(S77)}$$

We assume nominal wavelengths because the measured wavelengths are close to these (Fig. S4). For this analysis, the substrate parameters (e.g., the linear and quadratic curvatures) are not relevant, so the exact value of $n_{substrate}$ is not critical. For informational purposes, we consider a three-term Sellmeier equation and Ref. (*28*) for the relevant constants for synthetic sapphire; the equation yields indices of 1.7462 and 1.7599 for 1550 and 810 nm, respectively. Since the index is similar for both wavelengths, for simplicity we treat the two indices as approximately equal and fix $n_{substrate}$ to 1.75.

With these parameters, the fit returns an n_{film} of 3.3(3) for the quantum probe. As shown in Fig. S14, the fit curve closely tracks the experimental results. As an "effective" index of refraction, n_{film} may be used in analysis tasks as if our interferometer utilizes a single "wavelength" (the fringe period).

Refractive index: Classical probe

To obtain n_{film} for the classical 1550-nm probe, we attempted to utilize the same procedure we used for the quantum probe (described above). However, the lossy nature of the film yielded poor measurement results, including a severe drop in the interference visibility from 96.0(2)% for the uncoated region to 16.6(1)% for the coated region; these measurement results proved to be too large for effective correction during analysis.

Instead, we utilized ellipsometry, a standard technique for characterizing the refractive index of thin films. The coated region of the 50-nm calibration sample was measured directly with an ellipsometer; data was taken at six angles of incidence (15°, 25°, 35°, 45°, 55°, and 65°) for 710 nm through 1650 nm in 10-nm steps, except for 1350 through 1430 nm (because of an absorption band for a fiber within the instrument). Fitting the resulting data and assuming a 50-nm film thickness yielded a n_{film} of 3.07(5) at 1550 nm; the given error is the fitting error from the ellipsometer data analysis. Our value is close to values reported in the literature, e.g., Ref. (*29*) reports a refractive index of 3.14 at 0.77 eV (1610 nm) for a vacuum-evaporated nickel thin film.

Independent measurements of test sample film thickness

We performed three independent thickness measurements for the test sample (5-nm nominal thickness) using three established nano-characterization techniques: atomic force microscopy, scanning-stylus profilometry, and 3D optical profilometry. The details of these measurements are discussed below.

1. **Atomic force microscopy:** We utilized an Asylum Research MFP-3D atomic force microscope (AFM). A portion of the test sample containing both coated and uncoated regions was cleaved from the main wafer to accommodate sample insertion into the microscope.

   Scans over the uncoated-coated boundary resulted in unreliable thickness measurements as film non-uniformity near the boundary complicated the identification of the reference (uncoated region) and measured (coated region) heights. Film non-uniformity was prominent in these measurements because the AFM features high lateral

and vertical resolutions; a relatively small field of view (90 μm by 90 μm maximum) prevented us from performing large scans that incorporate regions away from the boundary that exhibit better uniformity.

We instead targeted a coated region away from the edge, where the film was observed to be highly uniform. The AFM tip was then brought into contact with the film to mechanically scrape away the film and expose the substrate, creating a sharp boundary between highly uniform coated and uncoated regions (as shown in Fig. 5B). The final image is 1.01 μm wide and 0.59 μm tall. Tilt correction was applied to the image by defining the substrate (uncoated) portion of the image as flat.

Given the high uniformity of the coated and uncoated regions, we obtained the thickness and its uncertainty by extracting a cross-sectional profile and averaging the first and last 340 nm of the coated and uncoated regions, respectively. The mean height values were subtracted to obtain the film thickness, and the standard deviations of the heights were combined via error propagation to obtain the uncertainty value.

2. **Scanning-stylus profilometry:** We utilized a Hysitron TI-950 TriboIndenter nanoindenter in scanning-probe mode: the probe was scanned over the boundary at a constant contact force while tracking the probe height. The instrument accommodated the test sample wafer in its entirety; no cleaving was required. An uncoated-coated boundary region was selected for measurement. A 40-μm by 19-μm image of the sample surface was generated.

   Tilt correction was applied to the image by defining the substrate (uncoated) portion of the image as flat. Five adjacent, parallel, and non-overlapping cross-sectional profiles were generated from the corrected image (Fig. S15). The profiles are approximately orthogonal to the uncoated-coated boundary (see Fig. S16B for an illustration of similar profile placement and orientation with respect to the boundary, but from a different measurement). Each profile is the average of 20 scan lines (20 pixels, 3.125 μm total width). As these profiles are similar in form to those obtained with our interferometer, we quantitively extract the film thickness from each profile by using (S73) as a fitting function. The five fitted thickness values have a mean fit error of 1%, indicating excellent fits to the data. The mean of these five thicknesses is taken as the final thickness, with the standard deviation as the measurement uncertainty.

3. **3D optical profilometry:** We utilized a Keyence VK-X1000 3D laser scanning confocal microscope. The instrument accommodated the test sample wafer in its entirety; no cleaving was required. An uncoated-coated boundary region was selected for measurement. A 282-μm by 212-μm image of the sample was taken.

   Six adjacent, parallel, and non-overlapping cross-sectional profiles were generated from this image (Fig. S16A). The profiles are approximately orthogonal to the uncoated-coated boundary, as can be seen in Fig. S16B. Each profile is the average of 101 scan lines (0.276 μm line spacing). We note that this measurement is close to the limit of the vertical resolution of confocal optical profilometry, which resulted in significantly noisier data compared to the other classical techniques tested. Since fitting the noisy data to (S73) yielded poor fits, we instead performed a semi-qualitative analysis to determine the height difference across the uncoated-coated boundary. For each profile, the heights of the uncoated and coated regions were estimated by identifying the

maximum and minimum vertical points of the step in the sample. The film thickness is the difference of these two points. The mean of the six resulting heights is taken as the final thickness, with the standard deviation as the measurement uncertainty.

Alternative interference modes

Classical frequency beating

Instead of starting with a non-degenerate polarization-entangled state (Eq. 4) and converting it to energy entanglement via propagation through a polarizing beamsplitter, we can start with a non-entangled, non-degenerate two-photon state of the form

$$\left|\varphi_{SPDC}\right\rangle = \left|\Theta\right\rangle_{1550}\left|\Theta\right\rangle_{810}, \tag{S78}$$

where Θ refers to a polarization basis state involving an equal superposition of $\left|H\right\rangle$ and $\left|V\right\rangle$, e.g., those from the diagonal or circular bases. Such a state may be generated with our entanglement source by pumping only the crystal that contributes to the $\left|H\right\rangle_{1550}\left|H\right\rangle_{810}$ term of Eq. 4 and rotating the compensation and tomography waveplates to transform $\left|H\right\rangle_{1550}\left|H\right\rangle_{810}$ into $\left|\Theta\right\rangle_{1550}\left|\Theta\right\rangle_{810}$.

With the polarization state being an equal superposition of $\left|H\right\rangle$ and $\left|V\right\rangle$, the polarizing beamsplitter is effectively a 50:50 beamsplitter such that the entire interferometer functions as an ordinary dual-frequency Mach-Zehnder interferometer. Indeed, the classical interference measurements reported in this work were performed by reconfiguring our experiment in the manner described in the paragraph above and monitoring the counts on the 1550-nm detectors (ignoring the 810-nm detectors). When analyzing the classical data (e.g., extracting the interference visibility), we utilize the normalized probability of photon detection in output port *A* of the interferometer (obtained by dividing the counts from the 1550*A* detector by the sum of the counts from the 1550*A* and 1550*B* detectors).

However, if we monitor both wavelengths, we can observe classical frequency beating. To model this behavior, we start with the fact that for a photon with a frequency of ω, the probability that the photon exits the interferometer in a particular output port is given by

$$\begin{aligned} P_A &= \sin^2\left(\frac{\varphi}{2}\right) \\ P_B &= \cos^2\left(\frac{\varphi}{2}\right), \end{aligned} \tag{S79}$$

where the *A* and *B* subscripts refer to interferometer output ports *A* and *B*, and $\varphi = \omega\tau$ is the relative phase between the two interferometer paths resulting from a relative temporal delay of τ.

In the dual-frequency case, two photons of frequencies ω_1 and ω_2, respectively, propagate through the same interferometer. We are interested in four possible outcomes: both photons exiting in (1) port *A* or (2) port *B*, (3) the ω_1 photon in port *A* and the ω_2 photon in port

B, and (4) the ω_1 photon in port B and the ω_2 photon in port A. Using (S79) and keeping track of the frequency-dependent relative phase, we conclude that the probabilities for these four outcomes are given by

$$\begin{aligned} P_{AA} &= \sin^2\left(\frac{\varphi_1}{2}\right)\sin^2\left(\frac{\varphi_2}{2}\right) \\ P_{BB} &= \cos^2\left(\frac{\varphi_1}{2}\right)\cos^2\left(\frac{\varphi_2}{2}\right) \\ P_{AB} &= \sin^2\left(\frac{\varphi_1}{2}\right)\cos^2\left(\frac{\varphi_2}{2}\right) \\ P_{BA} &= \cos^2\left(\frac{\varphi_1}{2}\right)\sin^2\left(\frac{\varphi_2}{2}\right), \end{aligned} \tag{S80}$$

where the AA subscript indicates that the ω_1 and ω_2 photons, respectively, both exit in port A, and so on. It then follows that the coincidence probability is given by

$$\begin{aligned} P_C &= \frac{P_{AB}+P_{BA}}{P_{AA}+P_{AB}+P_{BA}+P_{BB}} \\ &= \frac{1}{2}\left(1-\cos\left(2\varphi_1\right)\cos\left(2\varphi_2\right)\right), \end{aligned} \tag{S81}$$

via substitution of the probabilities from (S80) and trigonometric simplification.

We experimentally observed classical frequency beating (Fig. S17). The coincident detection fringes were fitted to fitting functions based on the form of the probabilities given in (S80). The coincident probability fringes were fitted to a fitting function based on the form of (S81). The single-detection fringes were fitted to a sinusoidal function, being standard classical single-photon interference fringes. We observe excellent agreement between the experimental data and the fits.

Quantum sum-frequency beating

While we are primarily interested in the two-photon interference effect arising from the energy-entangled state

$$|\psi\rangle = \frac{1}{\sqrt{2}}\left(|\omega_1\rangle_a|\omega_2\rangle_b + |\omega_2\rangle_a|\omega_1\rangle_b\right), \tag{S82}$$

a similar effect can be explored for the state in which both colors always travel the same path in the interferometer,

$$|\psi_{SF}\rangle = \frac{1}{\sqrt{2}}\left(|\omega_1\rangle_a|\omega_2\rangle_a + |\omega_2\rangle_b|\omega_1\rangle_b\right). \tag{S83}$$

Experimentally, in our system this would correspond to replacing the polarization-entangled input state with

$$\frac{1}{\sqrt{2}}\left(|H\rangle_{\omega_1}|H\rangle_{\omega_2}+|V\rangle_{\omega_1}|V\rangle_{\omega_2}\right). \tag{S84}$$

When the state $|\psi_{SF}\rangle$ travels through the interferometer, both photons will acquire a frequency-dependent-relative phase,

$$|\psi_{SF}\rangle\rightarrow\frac{1}{\sqrt{2}}\left(|\omega_1\rangle_a|\omega_2\rangle_a+e^{-i(\omega_1+\omega_2)\tau}|\omega_2\rangle_b|\omega_1\rangle_b\right). \tag{S85}$$

After the combining 50:50 beamsplitter,

$$\begin{aligned}|\psi_{SF}\rangle\rightarrow&\frac{1}{\sqrt{8}}\Big(\left(|\omega_1\rangle_a+i\,|\,\omega_1\rangle_b\right)\left(|\omega_2\rangle_a+i\,|\,\omega_2\rangle_b\right)+\\&+e^{-i(\omega_1+\omega_2)\tau}\left(|\omega_1\rangle_b+i|\omega_1\rangle_a\right)\left(|\omega_2\rangle_b+i|\omega_2\rangle_a\right)\Big)\\=&\frac{1}{\sqrt{8}}\Big(\left(1-e^{-i(\omega_1+\omega_2)\tau}\right)\left(|\omega_1\rangle_a|\omega_2\rangle_a-|\omega_1\rangle_b|\omega_2\rangle_b\right)+\\&+i\left(1+e^{-i(\omega_1+\omega_2)\tau}\right)\left(|\omega_1\rangle_a|\omega_2\rangle_b+|\omega_1\rangle_b|\omega_2\rangle_a\right)\Big),\end{aligned} \tag{S86}$$

leading to coincidence probability

$$P_C=\frac{1}{2}\left(1+\cos\left((\omega_1+\omega_2)\tau\right)\right). \tag{S87}$$

This fringe beats at the sum-frequency of the two photons, or, in the case of a photon pair generated via spontaneous parametric down-conversion, the pump frequency $\omega_p=\omega_1+\omega_2$.

Metrological measurements utilizing this sum-frequency beating effect can provide higher resolution per photon pair, with a Cramér-Rao bound of

$$\sigma_{\tau,CR,SF}\geq\frac{1}{\sqrt{N}}\frac{1}{\sqrt{(\omega_1+\omega_2)^2+4\sigma^2}}. \tag{S88}$$

However, since this interference requires both photons to travel the same path in the interferometer, it is susceptible to loss similar to classical interference. In Fig. S18, we demonstrate sum-frequency beating in our experimental system, measuring the interference pattern and resulting Fisher information. The coincidence probability P_C oscillates sinusoidally with a fitted period of 535.85(3) nm, close to the fitted 531.9120(5)-nm center wavelength of the entanglement source pump (Fig. S4).

Figures

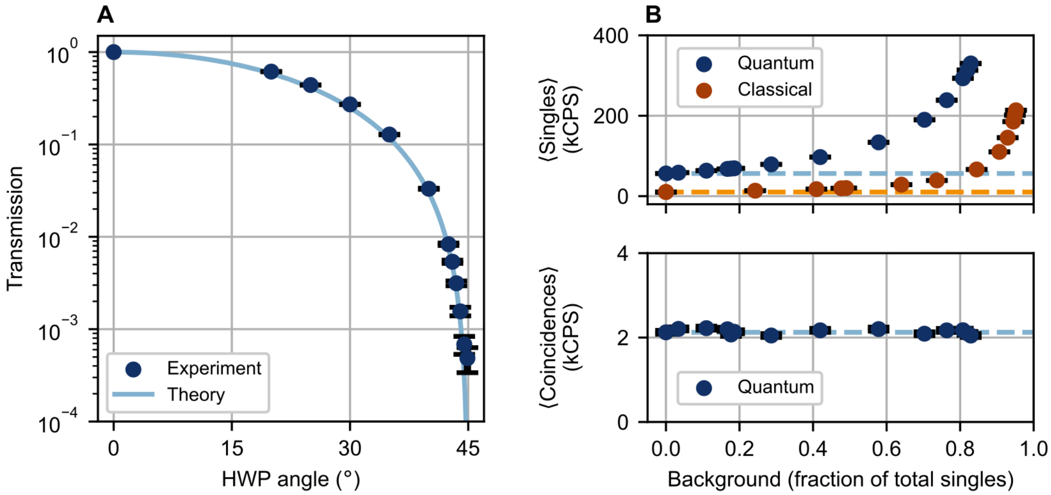

Fig. S1. Simulated optical loss and background. (A) We implement a tunable optical loss in mode *b* of the interferometer with a half-wave plate (HWP) followed by a polarizing beamsplitter (PBS). The observed PBS transmission for the quantum probe as a function of the HWP angle is in excellent agreement with the theoretical curve for an ideal HWP-PBS system obeying Malus' law (solid curve); the measured transmission does not reach zero when the angle approaches 45° because of a finite extinction ratio arising from HWP and PBS imperfections. **(B)** The mean total count rates for single detections (top) and coincident detections (bottom) as a function of simulated optical background, quantified as the fraction of total singles. The dashed lines indicate the zero-background baseline. Unlike the single-detection rate, which increases as background increases, the coincident detection rate is effectively independent of the background because of the tight coincident detection window used.

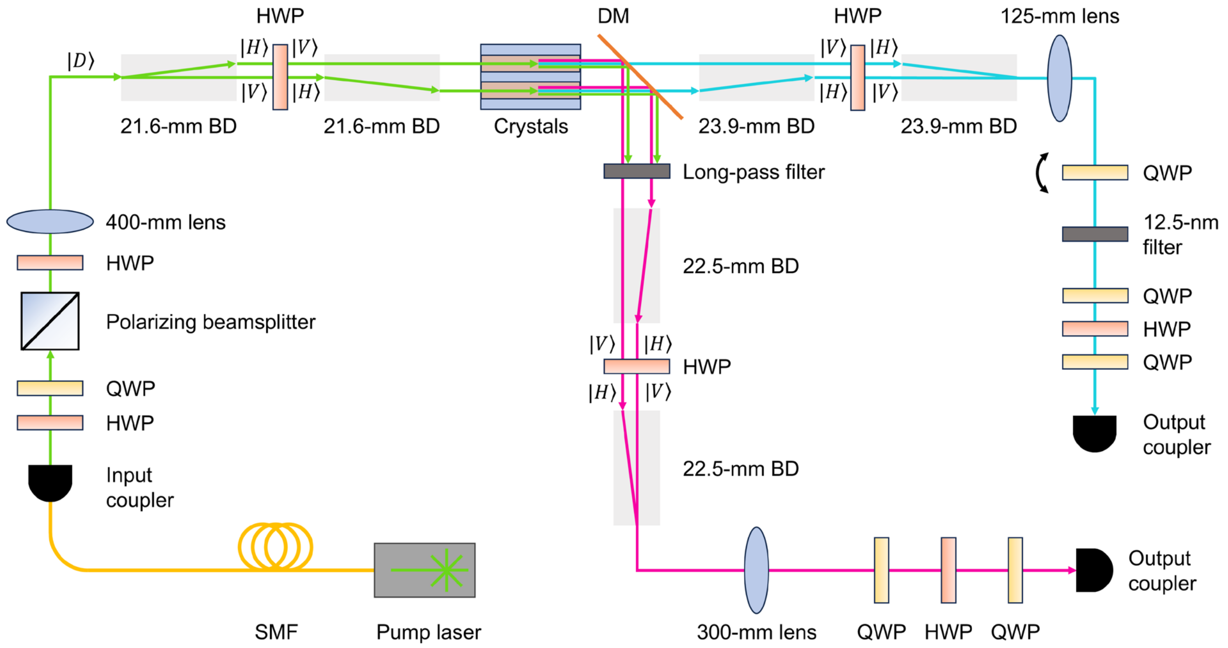

Fig. S2. Schematic of non-degenerate polarization entanglement source. A 532-nm (green) continuous-wave (CW) laser is polarization-filtered and focused into a pair of beam displacers (BDs), creating a polarization superposition of paths. Each beam is used to pump a MgO:PPLN down-conversion crystal, creating photon pairs at 810 nm (magenta) and 1550 nm (cyan) via Type-0 spontaneous parametric down-conversion (SPDC). A dichroic mirror (DM) separates the two wavelengths, after which the processes are recombined using a second pair of beam displacers in each arm, leading to a non-degenerate polarization-entangled state. Residual pump light is removed by interference filters, and the photons are coupled into single-mode fibers (SMFs). A trio of waveplates – two quarter-wave plates (QWP) and a half-wave plate (HWP) – built into the source is used to pre-compensate for any polarization transformations accrued in the fibers.

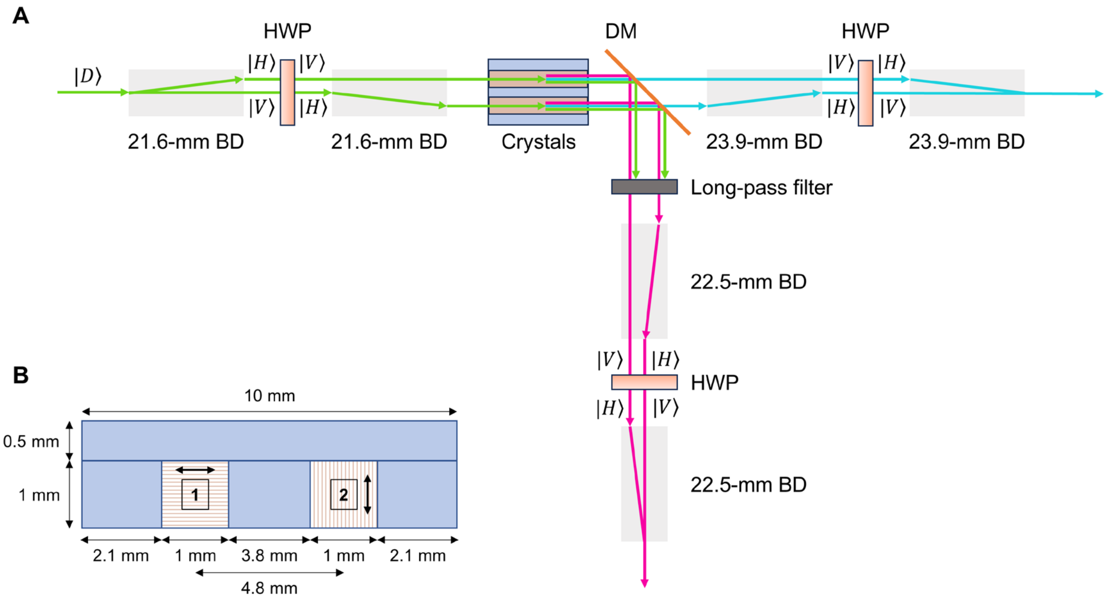

Fig. S3. Simplified schematic of non-degenerate polarization entanglement source. (A) Beam displacer interferometer, in which a diagonally polarized photon at 532 nm (green) is split via two beam displacers into a superposition of paths separated laterally by 4.8 millimeters. After down-conversion, the paths are recombined with a second set of beam displacers. **(B)** SPDC crystal mount, with two 1-mm x 1-mm x 20-mm MgO:PPLN crystals separated by 3.8-mm LN spacer and rotated 90° with respect to one another about the optical axis. This ensures that the center of each crystal aligns with one of the source interferometer beams.

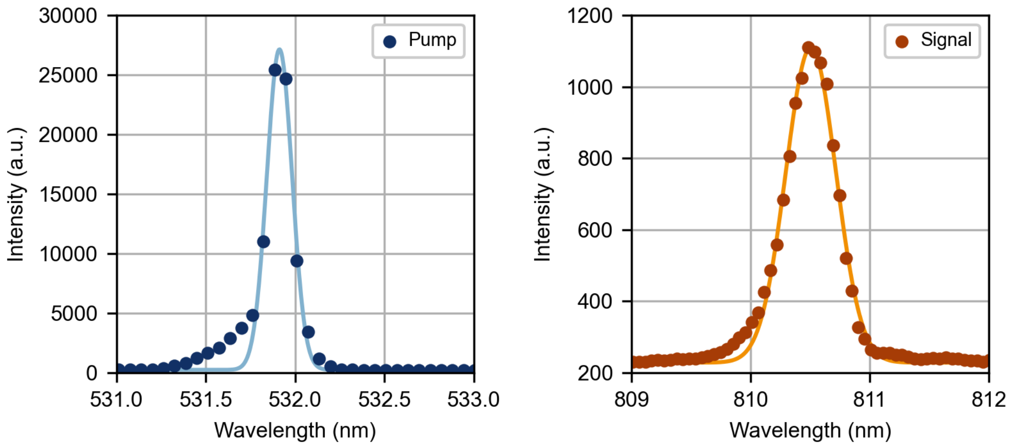

Fig. S4. Entanglement source pump and signal spectra. Gaussian fits to the pump (left) and signal (right) spectra yield center wavelengths of 531.9120(5) nm and 810.504(1) nm, close to the nominal wavelengths of 532 and 810 nm, respectively. Energy conservation implies an idler wavelength of 1547.484(5) nm, which is close to the nominal wavelength of 1550 nm. The given uncertainties are based on fit errors.

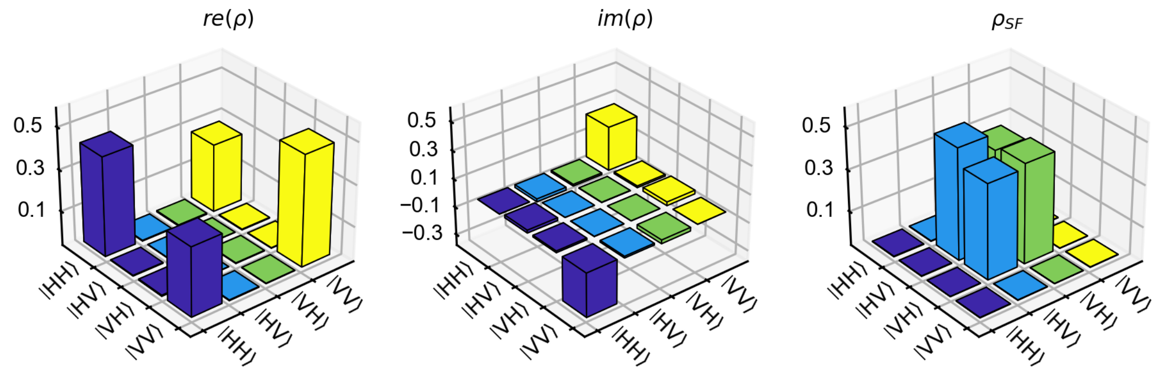

Fig. S5. Polarization-entangled state density matrix. The mean real (left) and imaginary (center) components of the density matrix ρ recovered during the state tomography step of the end-to-end system calibration protocol (Step 3). Identical to Fig. 1B, ρ_{SF} (right) illustrates how the unitary transformations realized via wave plates may be used to transform the state generated by the entanglement source (Eq. 4) and directly measured via state tomography into a given maximally entangled state, here chosen to be the nominal state required to generate energy entanglement ((S69)). The bit-flip step in the calibration protocol (Step 4) performs the unitary transformation to recover the singlet fraction matrix, up to a relative phase.

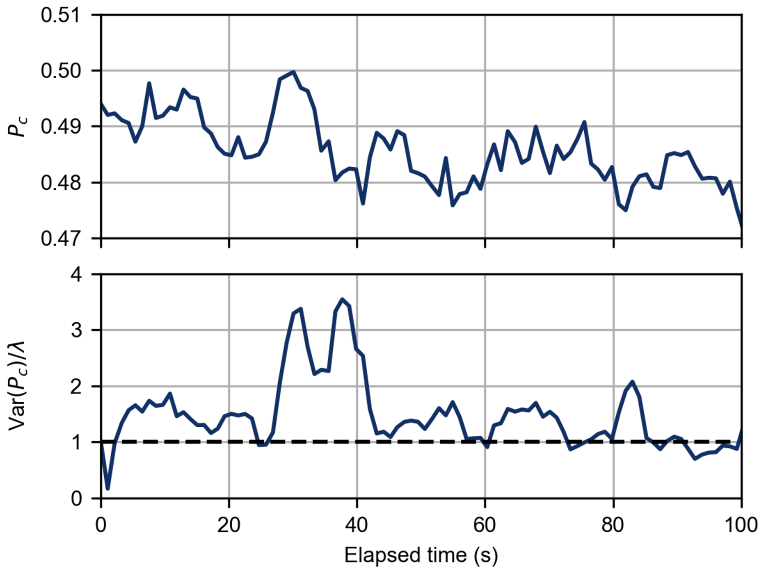

Fig. S6. Interferometer drift. Top panel: Time trace showing a typical drift in the interferometer phase, leading to a change in P_C, over 100 seconds. Bottom panel: The normalized noise (defined as the ratio of variance in P_C over Poissonian variance λ) for a 10-s rolling window. The black dashed line shows the expected trace $\left(\mathrm{Var}\left(P_C\right)/\lambda = 1\right)$ absent interferometer drift and other noise sources.

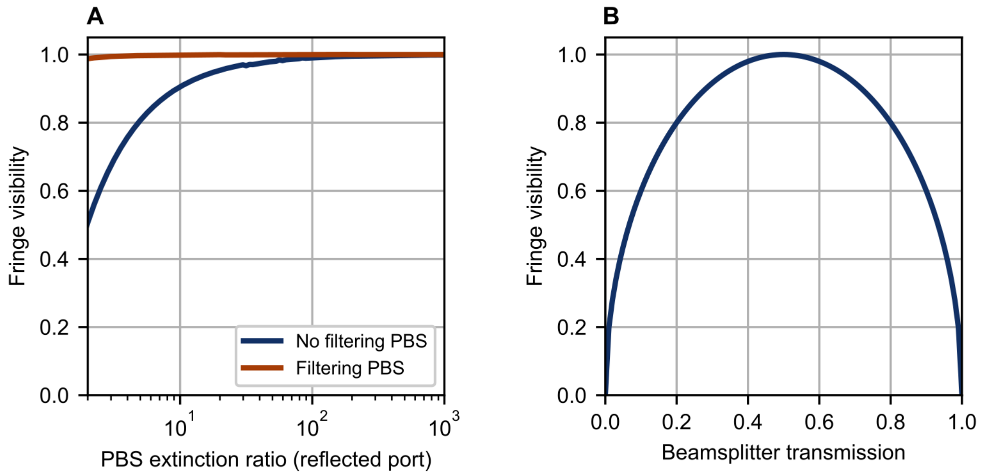

Fig. S7. Effects of system imperfections on fringe visibility. (A) The reduction in interference visibility resulting from imperfect polarizing beamsplitter (PBS) extinction ratio (ER). The transmission-port ER is fixed at 10,000, while the reflected-port ER is varied. Two scenarios are considered, one in which only a single PBS is used (blue), and one in which a second PBS is placed in the reflected arm of the interferometer to achieve additional polarization filtering (red). **(B)** The reduction in interference visibility resulting from imperfect nonpolarizing beamsplitter (NPBS) splitting ratio. Here the transmission coefficient T of the NPBS is varied for one wavelength in the system, with the reflection coefficient $R = 1 - T$ adjusted correspondingly. For the other wavelength, $T = R = 0.5$ is assumed.

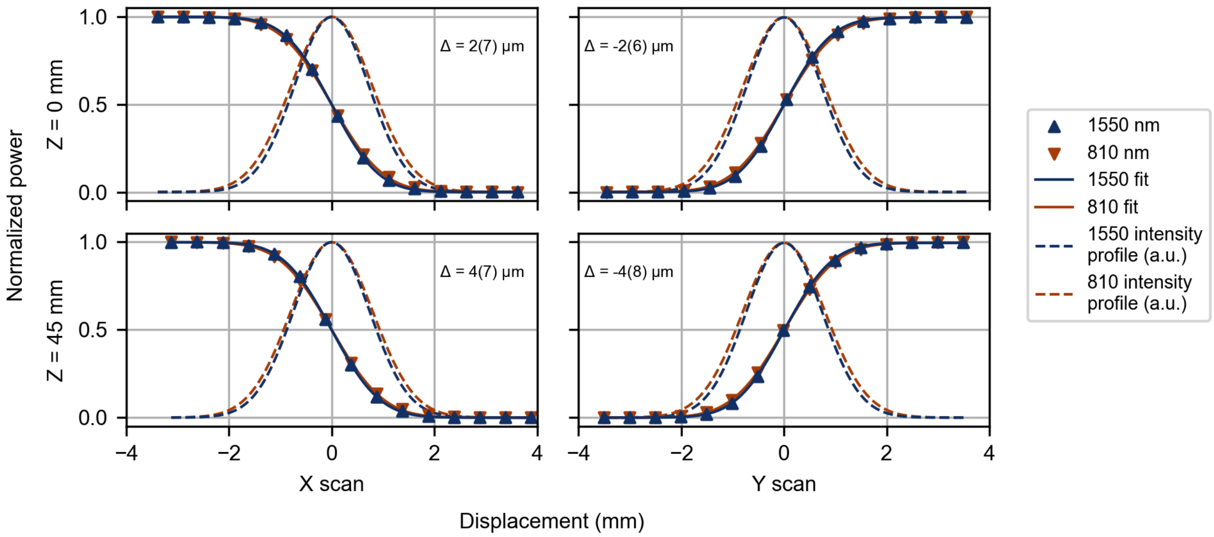

Fig. S8. Spatial overlap of the 1550-nm and 810-nm modes. We measure the spatial overlap of the 1550-nm and 810-nm modes at the input of our interferometer by performing knife-edge scans and fitting the curves to (S63) to obtain the beam centroid δ_0. The relative offsets of the 1550-nm and 810-nm centroids $\Delta \equiv \delta_0^{810} - \delta_0^{1550}$ are close to zero at two points along the multiplexed beam (separated longitudinally by 45 mm), indicating excellent spatial mode overlap and beam parallelism. The uncertainty in Δ is estimated via error propagation. To illustrate the overlap in the intensity profiles for both beams, we also plot the normalized magnitude (in arbitrary units) of the first derivative of the fitted curves.

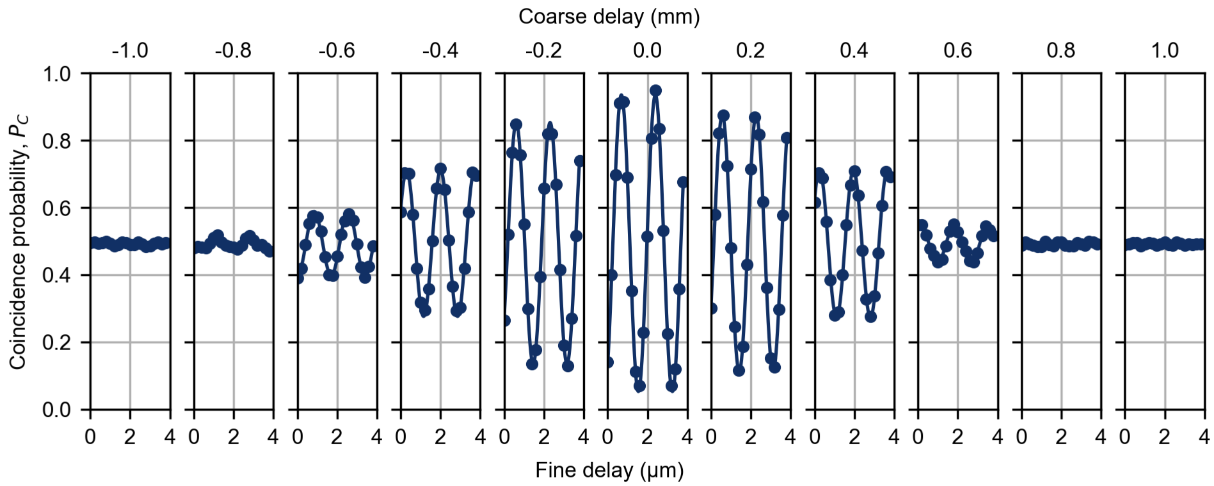

Fig. S9. Interference fringe envelope. A selection of individual fringe scans used to calculate the visibilities shown in Fig. 1D. The lower *x*-axis labels indicate the fine relative delay (piezoelectric nano-positioning stage) and the upper labels indicate the coarse relative delay (DC servo actuator) introduced by the optical trombone. A fine fringe scan was performed at each coarse delay position. The delays are in terms of optical path length. The solid curves are fits to the data.

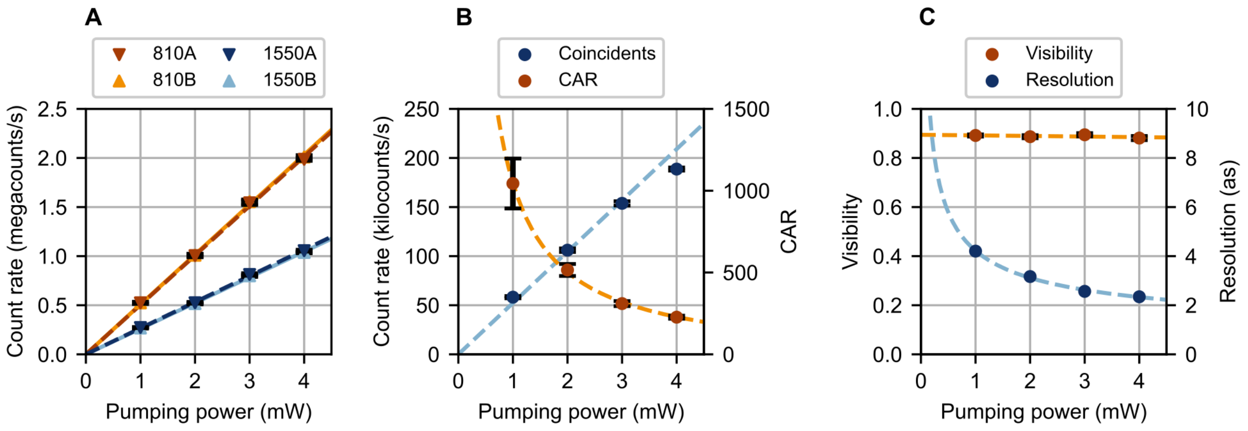

Fig. S10. System performance as a function of entanglement source pumping power. (A) The count rates on individual detectors scale linearly with the pump power, at least for count rates up to ~2 megacounts per second. This behavior is expected as the detector deadtime τ_{dt} is ≤20 ns for the 810-nm detectors and ≤30 ns for the 1550-nm detectors (vendor-supplied values); the nominal time tagger deadtime is ~2 ns. The corresponding detector saturation rate $1/\tau_{dt}$ is therefore at least ~50 and ~33 megacounts per second for the 810-nm and 1550-nm detectors, respectively. Since the *A* and *B* detectors are balanced for each wavelength as a part of the end-to-end system calibration protocol, their count rates appear almost identical. Solid and dashed lines are linear fits with the *y*-intercept fixed at zero. The fitted slopes for the 810*A* and 810*B* detectors are 503,753(5,031) and 508,167(4,450) counts per second per mW, respectively. The corresponding values for the 1550-nm detectors are 266,211(2,099) and 261,650(2,059) counts per second per mW, respectively. **(B)** The total coincident detection rate also scales linearly with the pump power but begins to saturate above 3 mW of pumping power. The dashed line is a linear fit with the *y*-intercept fixed at zero; the fitted slope is 52,270(1,297) counts per second per mW. The coincidence to accidental ratio (CAR) scales inversely with the pumping power and exhibits excellent agreement with the fit (dashed line). **(C)** The interference visibility is largely independent of the pumping power; a linear fit yields a slope of -0.2(3)% per mW and a *y*-intercept of 89.5(8)% (dashed line). The resolution scales as square root of the pump power and closely tracks the fit (dashed line).

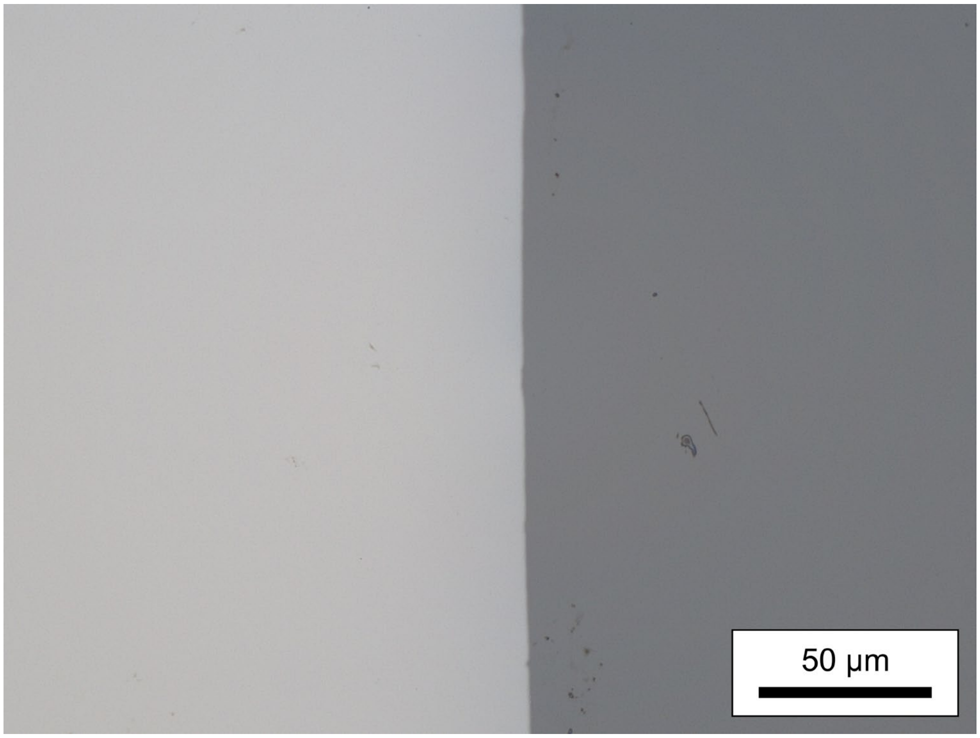

Fig. S11. Optical image of nickel thin film sample (5-nm thickness). The left side is the coated region, and the right is uncoated (bare substrate). Image taken with a Keyence VK-X1000 3D laser scanning confocal microscope.

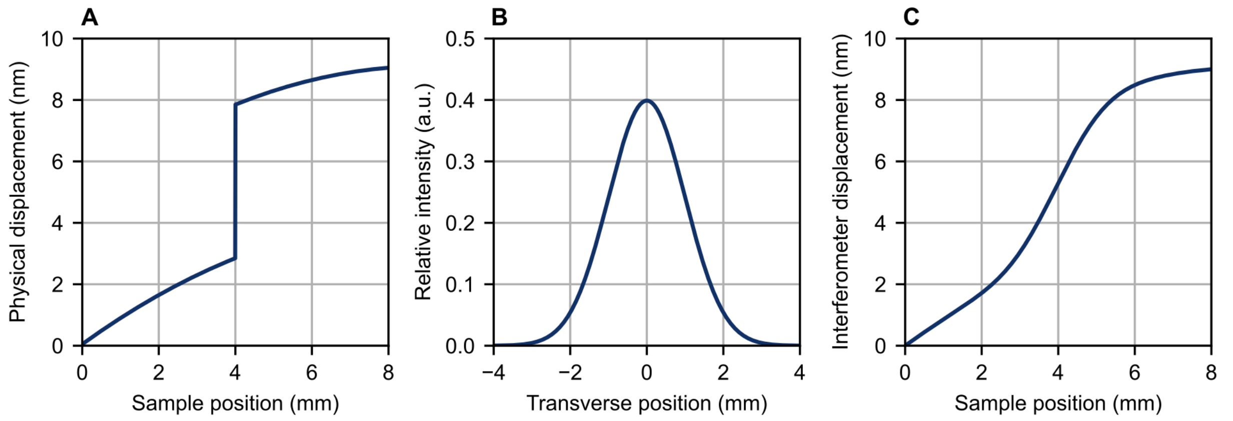

Fig. S12. Illustration of model for thin-film measurements. (A) Hypothetical sample consisting of a 5-nm thick film atop a substrate with a linear curvature of 0.5 nm/mm and a quadratic curvature of -0.05 nm/mm^2. The edge of the uncoated and coated regions is located at sample position 4 mm. **(B)** Hypothetical Gaussian transverse intensity distribution for the probe beam, with a full width at half maximum of $2\sqrt{2\ln 2}$ mm. **(C)** Hypothetical measurement of the sample shown in (A) using the probe beam geometry shown in (B). The resulting displacement versus position data is a convolution of (A) and (B). For this illustration, an effective refractive index of 2 is assumed for the film and substrate of the sample, and 1 for the ambient atmosphere.

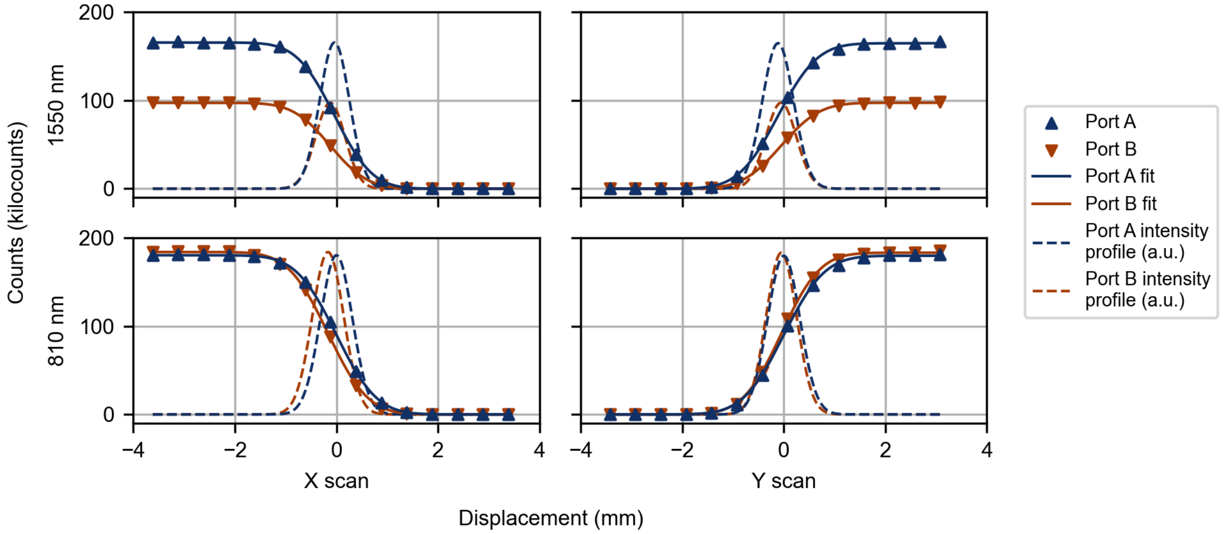

Fig. S13. Probe beam characterization. We characterize the spatial mode of the probe beam for both wavelengths by performing knife-edge scans and fitting the curves to (S63). To illustrate the intensity profiles for both beams, we also plot the normalized magnitude (in arbitrary units) of the first derivative of the fitted curves.

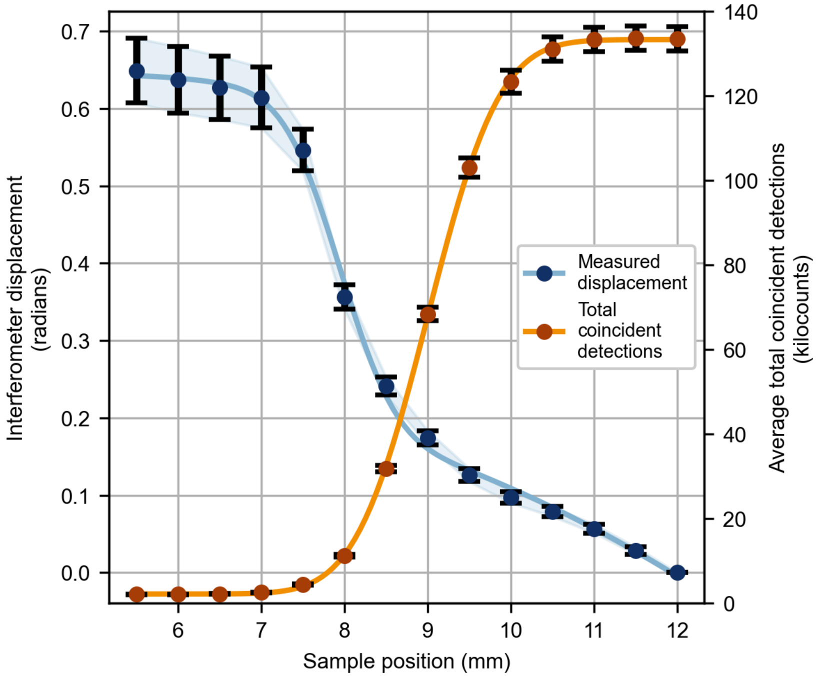

Fig. S14. Quantum interferometer calibration measurement. The interferometer displacement (in units of radians) and the average total coincident detections as a function of sample position for the calibration sample (50-nm thickness). The error bars show the standard deviation of 100 trials. For each trial, the scan started at position 12 mm, with an integration time of 1 second per point. The solid curves are fits to the data. A quantum probe transmission of 1.6(2)% is observed for the coated region (relative to the uncoated region), and fixing the film thickness at 50 nm yields an n_{film} of 3.3(3).

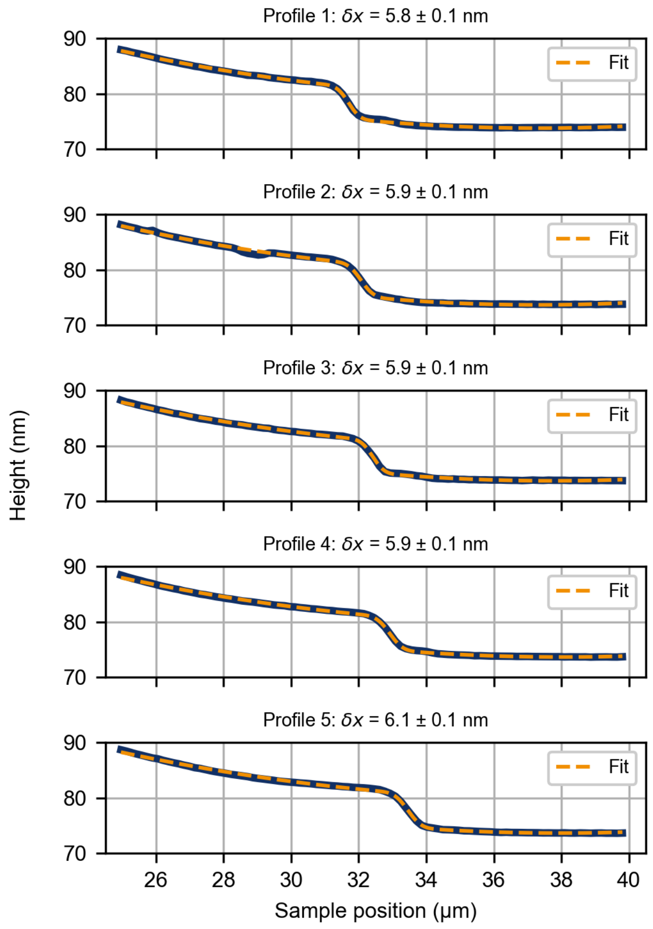

Fig. S15. Test sample thickness measurements via scanning-stylus profilometry. Measured height as a function of sample position for five adjacent, parallel, and non-overlapping cross-sectional profiles (solid curves). A change in height across the uncoated-coated boundary is clearly visible. The film thickness δx is extracted from fits to each profile (dashed curves).

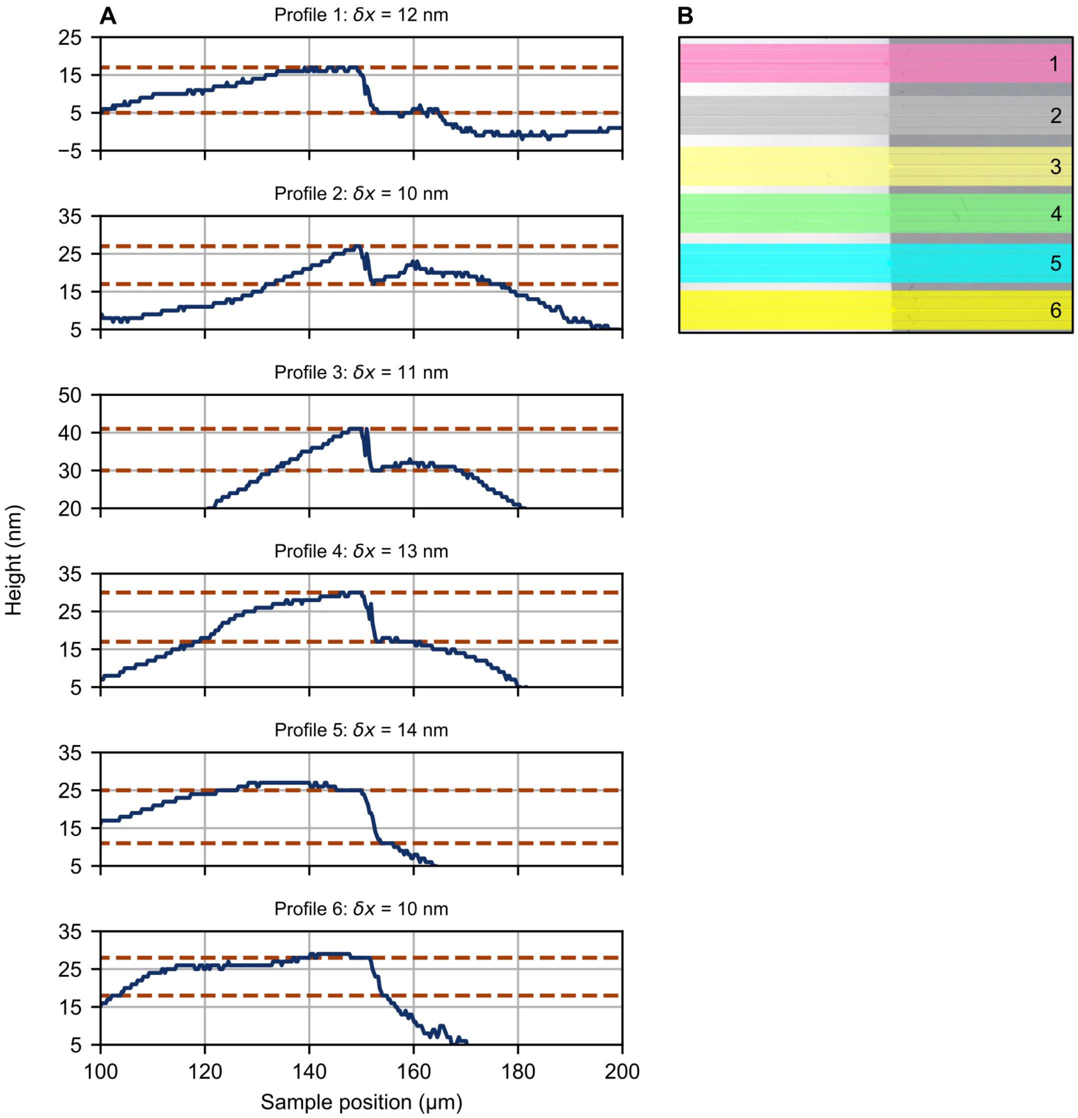

Fig. S16. Test sample thickness measurements via 3D optical profilometry. (A) Measured height as a function of sample position for six adjacent, parallel, and non-overlapping cross-sectional profiles (solid blue). A change in height across the uncoated-coated boundary is clearly visible. The dashed red lines indicate the heights used to calculate the film thickness δx. **(B)** Illustration of the regions from which the cross-sectional profiles were generated (superimposed on Fig. S11). The vertical width of each color band indicates the region where horizontal scan lines were averaged in generating a profile. The region numbering follows the profile numbering in (A).

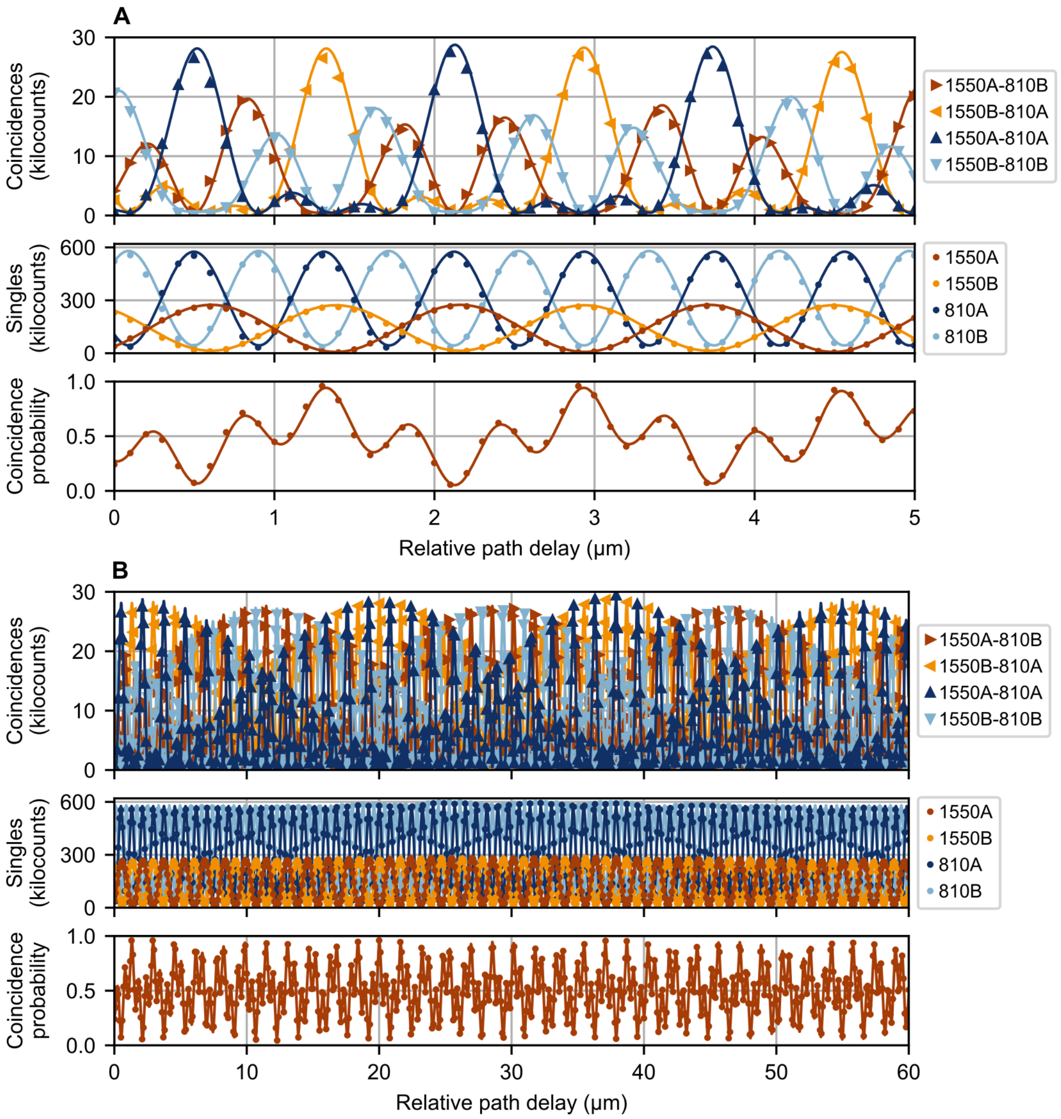

Fig. S17. Classical frequency beating. A zoomed-in section of a 60-μm scan is shown in **(A)** while the full scan is shown in **(B)**. Solid curves are fits; see text. Note the envelope beating with the coincidences fringes in (B). The four coincident detection fits have fitted photon wavelengths of 1554.32(4) nm and 812.96(4) nm for 1550*A*-810*B*, 1554.22(4) nm and 812.95(4) nm for 1550*B*-810*A*, 1554.27(4) nm and 812.98(4) nm for 1550*A*-810*A*, and 1554.25(4) nm and 812.95(4) nm for 1550*B*-810*B*. Similarly, the four single-detection fits return wavelengths of 1554.76(4) nm, 1554.75(4) nm, 812.98(1) nm, and 812.99(1) nm for 1550*A*, 1550*B*, 810*A*, and 810*B*, respectively. The coincidence probability fit returns photon wavelengths of 1554.67(5) and 812.90(2) nm. These fitted wavelengths are close to the actual photon wavelengths (Fig. S4) of 810.504(1) nm (measured) and 1547.484(5) (inferred via energy conservation). The fitted visibilities for the 1550*A*, 1550*B*, 810*A*, and 810*B* fringes are 95.0(2)%, 90.4(2)%, 85.9(2)%, and 86.0(2)%, respectively.

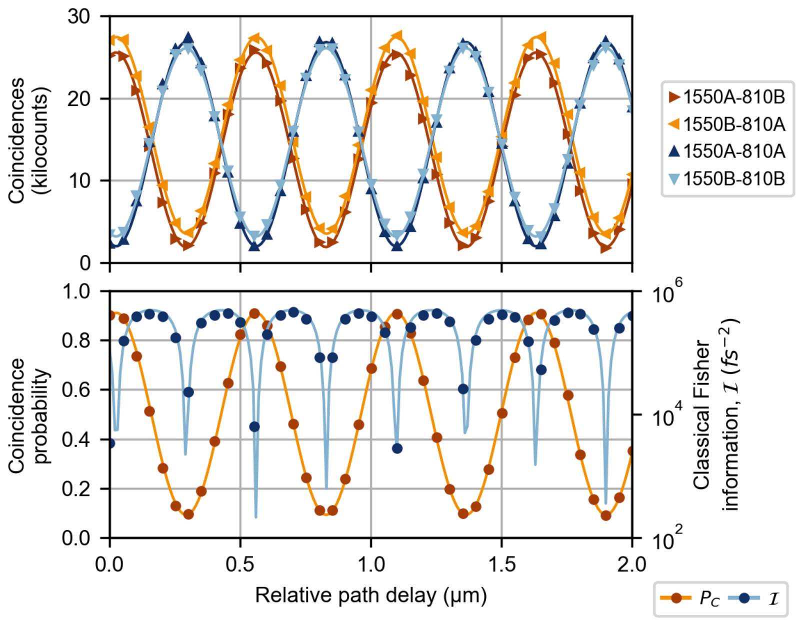

Fig. S18. Quantum sum-frequency beating. A 2-μm snippet of a 10-μm scan is shown; the fits (solid curves) utilize the full scan. As the photons' relative time of arrival τ is scanned by adjusting the relative path delay between modes a and b, P_C oscillates sinusoidally with a fitted period of 535.85(3) nm, close to the 531.9120(5) nm center wavelength of the entanglement source pump (Fig. S4). The fringe visibility is 81.6(2)%. Fits to the four coincident detection fringes yield visibilities of 86.3(3)%, 77.3(2)%, 86.3(3)%, and 78.3(2)% for 1550A-810B, 1550B-810A, 1550A-810A, and 1550B-810B, respectively. In contrast, fits to the four individual-detector fringes yield visibilities of 0.8(1)%, 0.5(1)%, 0.9(1)%, and 1.3(1)% for 1550A, 1550B, 810A, and 810B, respectively. With 1 mW of pumping power for the entanglement source and an integration time of 1 second, we observe a mean of 58,200(600) total coincident detections per measurement. The corresponding resolution extracted from the Fisher information is 0.43 nm (1.43 as), which represents an 82% saturation of the Cramér–Rao bound, assuming we have (S83) as our probe state. For 10,000 coincident detections, the expected experimental resolution is 3.5 as, which contrasts with 10.2 as for the difference-frequency beating (the main effect considered in this work), despite the latter achieving an 88% saturation of the Cramér–Rao bound. This enhancement occurs because of the shorter fringe period.

Tables

Detector channel	*Design wavelength*	*Detector absolute efficiency, Vendor measured*	*Fiber link transmission, measured*	*Detector FWHM timing jitter, Vendor measured*	*Time-tagger channel FWHM timing jitter, nominal*
1550*A*	1590 nm	89(2)% (1570 nm)	~100% (1550 nm)	51 ps	14 ps
1550*B*	1590 nm	87(2)% (1570 nm)	~100% (1550 nm)	48 ps	14 ps
810*A*	777 nm	101(4)% (778 nm)	~91% (810 nm)	41 ps	14 ps
810*B*	777 nm	100(4)% (778 nm)	~97% (810 nm)	54 ps	14 ps

Table S1. Detector specifications. The detector absolute efficiency is measured from the optical input at the detector front panel and includes front-panel insertion loss. Optimal incident polarization is assumed. The given approximate fiber link transmission values are intended to illustrate the absence of relatively large sources of loss between the fiber couplers and the detectors; a precise characterization is beyond the scope of this work. These values do not include the fiber-coupling losses in the detection module (see Table S3) but include fiber mating loss at the input to the fiber link. Also not included are small additional losses (insertion and transmission) from a short patch fiber connecting the fiber link to the detector front panel; their precise values are unknown but are estimated to be <10%.

Optical Path			*Transmission*
Fiber Launch	*Free-Space Path*	*Fiber Coupler*	
1550	Transmitted	1550*A*	~16%
1550	Reflected	1550*A*	~20%
1550	Transmitted	1550*B*	~9%
1550	Reflected	1550*B*	~35%
		TOTAL	~80%
810	Transmitted	810*A*	~19%
810	Reflected	810*A*	~21%
810	Transmitted	810*B*	~19%
810	Reflected	810*B*	~21%
		TOTAL	~79%

Table S2. Interferometer module free-space transmission. Transmission is defined as the quotient of the power immediately exiting the input fiber launch and the power incident on the output fiber coupler (see the interferometer and detection modules in Fig. 1A). Transmission through the transmitted and reflected paths of the interferometer is measured separately by blocking the transmitted and reflected paths as appropriate. Because of system geometry, the 1550*A*, 1550*B*, and 810*A* measurements include loss from a single reflection off a mirror. However, since the dielectric mirrors used have high reflectivity, their contribution to loss can be assumed to be negligible. The power split between the transmitted and reflected paths depends on the polarization of the incident light. The approximate values given are intended to illustrate the relatively high transmission of the interferometer; a precise characterization is beyond the scope of this work.

Detection Fiber	*Coupling efficiency*		
	Transmitted path	*Reflected path*	*Average*
1550*A*	~87%	~90%	~88.5%
1550*B*	~89%	~89%	~89%
810*A*	~92%	~92%	~92%
810*B*	~97%	~95%	~96%

Table S3. Detection module fiber-coupling efficiencies. Coupling efficiency is defined as the quotient of the power incident on the fiber coupler and the power exiting the other end of the fiber. Note that the exiting end has no anti-reflection coating, which contributes ~4% of loss to the measured efficiency. All measurements were made using classical alignment lasers and power meters at the suitable wavelength. Because of system geometry, the 1550*A*, 1550*B*, and 810*A* measurements include loss from a single reflection off a mirror. However, since these dielectric mirrors have high reflectivity, their contributions to loss can be assumed to be negligible. The approximate values given are intended to illustrate the relatively high and balanced coupling efficiencies realized; a precise characterization is beyond the scope of this work.

Coincident detection channel	*Estimated FWHM, timing jitter*	*Observed FWHM, detector correlation*
1550*A*-810*A*	68 ps	70.4(0.2) ps
1550*A*-810*B*	77 ps	80.2(0.4) ps
1550*B*-810*A*	66 ps	69.4(0.2) ps
1550*B*-810*B*	75 ps	79.5(0.4) ps

Table S4. Coincident detection jitter. By summing (in quadrature) the vendor-supplied full width at half maximum (FWHM) jitter for the two detector and time-tagger channels for each coincident detection channel, we obtain an estimate for the combined jitter for each channel. These values are comparable to the FWHM of the correlation peak for each detector pair, which was obtained by fitting the correlation peak with a Gaussian function. The given FWHM error is the fitting error.

Basis state	*Quarter-wave plate #1*	*Half-wave plate*	*Quarter-wave plate #2*
$\|H\rangle$	0°	0°	0°
$\|V\rangle$	0°	45°	0°
$\|D\rangle$	45°	22.5°	0°
$\|A\rangle$	45°	-22.5°	0°
$\|L\rangle$	45°	0°	0°
$\|R\rangle$	45°	45°	0°

Table S5. State tomography angles. Each state projector corresponds to a set of angles for the three waveplates (for each wavelength) in the source module. The shown angles are relative to the waveplates' "zero" angles (i.e., corresponding to the waveplates' fast or slow axes) obtained during calibration.

Metric	*Specification*
Roughness	<0.3 nm
Warp	≤15 µm
Bow	≤10 µm
TTV	<3 µm
LTV	≤1.5 µm

Table S6. Key sapphire wafer specifications. Vendor supplied values. TTV: Total thickness variation. LTV: Local thickness variation (5 × 5 mm).

REFERENCES AND NOTES

1. LIGO Scientific Collaboration and Virgo Collaboration, Observation of gravitational waves from a binary black hole merger. *Phys. Rev. Lett.* **116**, 061102 (2016).

2. J. D. Monnier, Optical interferometry in astronomy. *Rep. Prog. Phys.* **66**, 789–857 (2003).

3. D. Huang, E. A. Swanson, C. P. Lin, J. S. Schuman, W. G. Stinson, W. Chang, M. R. Hee, T. Flotte, K. Gregory, C. A. Puliafito, J. G. Fujimoto, Optical coherence tomography. *Science* **254**, 1178–1181 (1991).

4. C. K. Hong, Z. Y. Ou, L. Mandel, Measurement of subpicosecond time intervals between two photons by interference. *Phys. Rev. Lett.* **59**, 2044–2046 (1987).

5. A. F. Abouraddy, M. B. Nasr, B. E. A. Saleh, A. V. Sergienko, M. C. Teich, Quantum-optical coherence tomography with dispersion cancellation. *Phys. Rev. A* **65**, 053817 (2002).

6. M. B. Nasr, B. E. A. Saleh, A. V. Sergienko, M. C. Teich, Demonstration of dispersion-canceled quantum-optical coherence tomography. *Phys. Rev. Lett.* **91**, 083601 (2003).

7. B. Ndagano, H. Defienne, D. Branford, Y. D. Shah, A. Lyons, N. Westerberg, E. M. Gauger, D. Faccio, Quantum microscopy based on Hong–Ou–Mandel interference. *Nat. Photon.* **16**, 384–389 (2022).

8. T. B. Bahder, W. M. Golding, Clock synchronization based on second-order quantum coherence of entangled photons. *AIP Conf. Proc.* **734**, 395–398 (2004).

9. M. Xie, H. Zhang, Z. Lin, G.-L. Long, Implementation of a twin-beam state-based clock synchronization system with dispersion-free HOM feedback. *Opt. Express* **29**, 28607–28618 (2021).

10. A. Lyons, G. C. Knee, E. Bolduc, T. Roger, J. Leach, E. M. Gauger, D. Faccio, Attosecond-resolution Hong-Ou-Mandel interferometry. *Sci. Adv.* **4**, eaap9416 (2018).

11. M. B. Nasr, O. Minaeva, G. N. Goltsman, A. V. Sergienko, B. E. A. Saleh, M. C. Teich, Submicron axial resolution in an ultrabroadband two-photon interferometer using superconducting single-photon detectors. *Opt. Express* **16**, 15104–15108 (2008).

12. M. Okano, H. H. Lim, R. Okamoto, N. Nishizawa, S. Kurimura, S. Takeuchi, 0.54 μm resolution two-photon interference with dispersion cancellation for quantum optical coherence tomography. *Sci. Rep.* **5**, 18042 (2016).

13. S. Singh, V. Kumar, V. Sharma, D. Faccio, G. K. Samanta, Near-video frame rate quantum sensing using Hong–Ou–Mandel interferometry. *Adv. Quantum Technol.* **6**, 2300177 (2023).

14. Y. Chen, M. Fink, F. Steinlechner, J. P. Torres, R. Ursin, Hong-Ou-Mandel interferometry on a biphoton beat note. *npj Quantum Inf.* **5**, 43 (2019).

15. Z. Y. Ou, L. Mandel, Observation of spatial quantum beating with separated photodetectors. *Phys. Rev. Lett.* **61**, 54–57 (1988).

16. J. G. Rarity, P. R. Tapster, Two-color photons and nonlocality in fourth-order interference. *Phys. Rev. A* **41**, 5139–5146 (1990).

17. C. W. Helstrom, Quantum detection and estimation theory. *J. Stat. Phys.* **1**, 231–252 (1969).

18. A. Fujiwara, H. Nagaoka, Quantum Fisher metric and estimation for pure state models. *Phys. Lett. A* **201**, 119–124 (1995).

19. S. Ramelow, L. Ratschbacher, A. Fedrizzi, N. K. Langford, A. Zeilinger, Discrete tunable color entanglement. *Phys. Rev. Lett.* **103**, 253601 (2009).

20. P. G. Evans, R. S. Bennink, W. P. Grice, T. S. Humble, J. Schaake, Bright source of spectrally uncorrelated polarization-entangled photons with nearly single-mode emission. *Phys. Rev. Lett.* **105**, 253601 (2010).

21. C. Torre, A. McMillan, J. Monroy-Ruz, J. C. F. Matthews, Sub-μm axial precision depth imaging with entangled two-color Hong-Ou-Mandel microscopy. *Phys. Rev. A* **108**, 023726 (2023).

22. M. B. Nasr, S. Carrasco, B. E. A. Saleh, A. V. Sergienko, M. C. Teich, J. P. Torres, L. Torner, D. S. Hum, M. M. Fejer, Ultrabroadband biphotons generated via chirped quasi-phase-matched optical parametric down-conversion. *Phys. Rev. Lett.* **100**, 183601 (2008).

23. H. Lee, P. Kok, J. P. Dowling, A quantum Rosetta stone for interferometry. *J. Mod. Opt.* **49**, 2325–2338 (2002).

24. T. B. Pittman, D. V. Strekalov, A. Migdall, M. H. Rubin, A. V. Sergienko, Y. H. Shih, Can two-photon interference be considered the interference of two photons? *Phys. Rev. Lett.* **77**, 1917–1920 (1996).

25. A. M. Brańczyk, Hong-Ou-Mandel Interference. arXiv:1711.00080 (2017).

26. D. N. Klyshko, Use of two-photon light for absolute calibration of photoelectric detectors. *Sov. J. Quantum Electron.* **10**, 1112–1117 (1980).

27. J. B. Altepeter, E. R. Jeffrey, P. G. Kwiat, "Photonic State Tomography" in *Advances In Atomic, Molecular, and Optical Physics*, P. R. Berman, C. C. Lin, Eds. (Academic Press, 2005), vol. **52**, pp. 105–159.

28. I. H. Malitson, Refraction and dispersion of synthetic sapphire. *J. Opt. Soc. Am.* **52**, 1377–1379 (1962).

29. P. B. Johnson, R. W. Christy, Optical constants of transition metals: Ti, V, Cr, Mn, Fe, Co, Ni, and Pd. *Phys. Rev. B* **9**, 5056–5070 (1974).
